# Simultaneous complementary photoswitching of hemithioindigo tweezers for dynamic guest relocalization

**DOI:** 10.1038/s41467-018-03912-7

**Published:** 2018-04-13

**Authors:** Sandra Wiedbrauk, Thomas Bartelmann, Stefan Thumser, Peter Mayer, Henry Dube

**Affiliations:** Department of Chemistry, Ludwig-Maximilians-Universität München, Butenandtstrasse 5-13, München, 81377 Germany

## Abstract

Remote control of complex molecular behavior and function is one key problem in modern chemistry. Using light signaling for this purpose has many advantages, however the integration of different photo processes into a wholesome yet complex system is highly challenging. Here we report an alternative approach to increase complexity of light control-simultaneous complementary photoswitching-in which spectral overlap is used as an advantage to drastically reduce the signaling needed for controlling multipart supramolecular assemblies. Two photoswitchable molecular tweezers respond to the same light signals with opposite changes in their binding affinities. In this way the configuration of two host tweezers and ultimately the dynamic relocation of a guest molecule can be trigged by only one signal reversibly in the same solution. This approach should provide a powerful tool for the construction of sophisticated, integrated, and multi-responsive smart molecular systems in any application driven field of chemistry.

## Abstract (web summary)

Controlling complex photoresponsive systems while minimizing light input is highly challenging. Here, the authors report two photoswitchable molecular tweezers responding to the same light signals with opposite changes in their binding affinities towards a guest molecule allowing for its “light-economic” relocation.

## Introduction

Gaining photocontrol over molecular processes is a field of tremendous current interest as it allows to directly manipulate matter at the molecular scale with high precision and spatio-temporal resolution. Intriguing properties and functions have been realized with photoswitches ranging from light control of supramolecular recognition^1–5^ and molecular machines^6–12^, to biological processes^13–19^, smart materials^20–24^, nano systems^25–27^, or soft matter^28,29^, as well as light responsive catalysis^30^ - where even control over the stereochemical outcome is possible^31^.

In most applications one light responsive moiety is employed as relay-component that translates light signals into molecular changes resulting in either one-time responsiveness as in the case of photolabile groups or reversible changes as in the case of photoswitches. To escalate the complexity of the light signaling the combination of different light responsive elements into the same system is a logical next step and many current efforts have now moved in this direction^2,28,32–35^.

The main focus of these efforts has been selective and independent addressability of the different light responsive moieties to ascertain maximum control of individual processes. Such orthogonal light control is easier to establish in one-time responsive systems such as photolabile protecting groups^36–38^ and has been achieved for photoswitches only recently in a satisfying manner^39–42^. Another very promising approach combines light with other types of signals such as electro chemistry^43^ or addition of reagents^44–48^. Highly selective multi-component photoswitching has been achieved by combining different types of photoswitches^2^ and even within the same class of chromophores^34,49^.

A different solution to the problem of controlling multiple molecular processes with external signals has just very recently been conceptualized by the group of David Leigh^50^. Therein it was proposed to affect multiple events by the same signal to drastically reduce the overall triggering needed. We were independently working on a similar method using exclusively light as signal to control molecular tweezers systems^1,51–59^ based on the hemithioindigo (HTI) chromophore^60–67^.

Herein we present the result of our efforts, an approach that we call simultaneous complementary photoswitching. We use the same wavelength of light to affect two independent photoswitching events in the same solution, which are nonetheless functionally coupled leading to a wholesome supramolecular system. This approach opens up interesting possibilities for the generation of programmed complex molecular behavior as it enables one external signal to affect multiple coupled components in the system - each in a unique and predictable way. As a showcase example we present two photoresponsive molecular tweezers **1** and **2**. Similar to the helical molecular receptor that was established earlier in our group tweezers **1** and **2** bind electron deficient aromatic guests via polar aromatic interactions in a 1:1 stoichiometry. However, different to the earlier system, we have achieved significantly higher binding constants in the high affinity conformations and can now establish both, high, as well as low binding affinities towards an external guest molecule by visible light signals. A mixture of both tweezers **1** and **2** in solution enables the dynamic relocation of a guest molecule via simultaneous complementary photoswitching using only one light signal.

## Results

### Molecular HTI tweezers

**Fig. 1:**
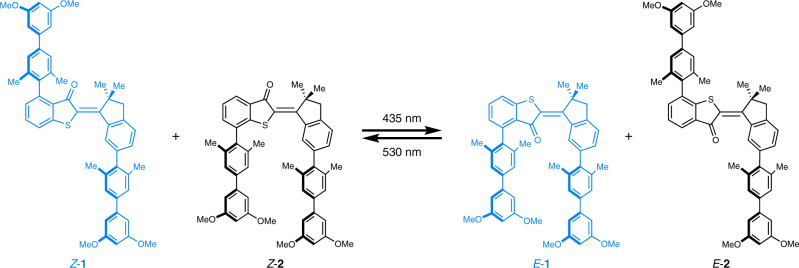
Simultaneous complementary photoswitching of HTI tweezers. Two simultaneous processes are encountered at each wavelength of irradiation: at 435 nm tweezers **1** transform into their high affinity form (*E*-**1**) while tweezers **2** isomerize into their low affinity form (*E*-**2**). At 530 nm the processes are reversed and *Z*-**1** and *Z*-**2** are restored

As can be seen from Fig. 1 photoresponsive tweezers **1** and **2** are constructed in a complementary fashion. Tweezers **1** possess two electron rich biphenyl arms on either side of the photoisomerizable double bond, which are directed opposite of each other in the thermodynamically stable *Z* isomeric state. The two arms therefore cannot be used for recognition of electron poor aromatic guests in a sandwich-type stacking mode resulting in an expected low affinity of the *Z* isomer. However, *Z* to *E* photoisomerization with blue light (435 nm) establishes close proximity of the two arms in the *E* configuration and allows for polar aromatic stacking interactions with electron deficient guest molecules. In the *E* configuration an aromatic guest is therefore expected to efficiently bind to tweezers **1**. The reverse *E* to *Z* photoisomerization can be achieved effectively with green light (530 nm) restoring the non-binding state of tweezers **1**. Tweezers **2** were designed to express the exact complementary behavior by attaching the two electron rich biphenyl units in opposite fashion. In the *Z* conformation of **2** the biphenyl arms are pointing towards the same side and strong guest binding should be observed. After *Z* to *E* photoisomerization the binding of an electron poor aromatic guest is disrupted in *E*-**2**-similarly to *Z*-**1**. As the overall electronic setup of both tweezers **1** and **2** is similar and the different substitution pattern do not perturb their absorption properties significantly *Z* to *E* and *E* to *Z* photoisomerization can each be affected by the same wavelengths (Fig. 1). In a mixture of **1** and **2** it should therefore be possible to couple the two opposite binding affinity changes of the two separate tweezers by the same light signaling event. As a result only one signal would control two interlinked events at the same time resulting in the overall defined relocalization of a guest molecule.

### Synthesis

**Fig. 2:**
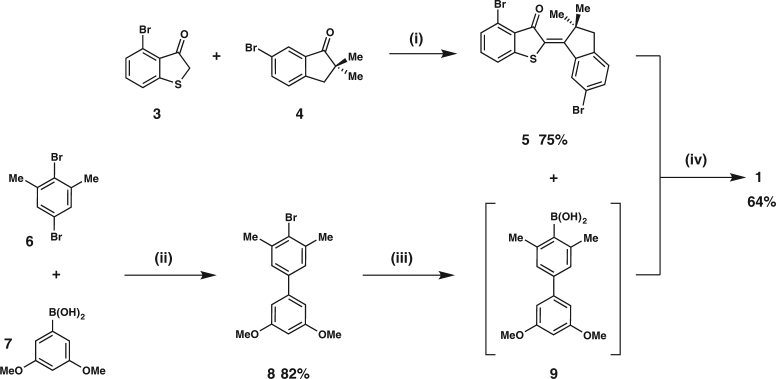
Synthesis of tweezers **1** via a convergent route. (i) BCl_3_, THF, 0 °C, 20 min. (ii) K_2_CO_3_, Pd(PPh_3_)_4_, 1,2-dimethoxyethane, H_2_O, 84 °C, 13 h. (iii) First *sec*-BuLi, THF, −78 °C, 30 min, then B(OMe)_3_, –78 °C, 15 min, then 23 °C, 30 min, then HCl acidic workup. (iv) sSPhos Pd G2, K_3_PO_4_, toluene, EtOH, H_2_O, 80 °C, 18 h

The convergent synthesis of tweezers **1** is shown in Fig. 2 exemplarily, while synthesis of tweezers **2** is described in the Supplementary Methods. The synthesis starts with a condensation reaction between the known brominated thiophenone **3** and brominated indanone **4** in the presence of BCl_3_ as Lewis acid. Formation of the twofold brominated HTI **5** poceeds in a good yield of 75%. A Suzuki-cross coupling reaction between dibrominated xylene **6** and boronic acid **7** gives the corresponding brominated biphenyl **8** in 82%. Successive bromine-lithium exchange, transmetallation with B(OMe)_3_, and acidic hydrolysis gave boronic acid **9**, which was used without further purification in the next step. A final twofold palladium catalyzed Suzuki-cross coupling reaction between biphenyl boronic acid **9** and HTI **5** yielded tweezers **1** in 64%.

### Conformational analysis

The conformations of both tweezers **1** and **2** have been investigated in CDCl_3_ solution for each isomeric form (*Z* and *E* configuration) using NMR spectroscopy. The general features are described below, the detailed discussion is given in Supplementary Fig. 1, Supplementary Table 15, and Supplementary Note 1.

In general the conformation of **1** and **2** is restricted to rotation around the single bonds of the two biphenyl units. The central HTI unit is rigid and allows only one geometric arrangement of the binding biphenyl units for each isomeric form. In order to preorganize the tweezers in their high-affinity-or ON-states (*E*-**1** and *Z*-**2**) for the intended aromatic stacking interactions we have introduced methyl groups in the *ortho*-positions of the attached biphenyl units. Because of steric repulsion the biphenyl moieties cannot reside in a conjugated coplanar arrangement with the HTI core structure. Instead they are twisted out of HTI conjugation and align in a parallel fashion to each other. Their distance is controlled by the particular substitution pattern and based on quantum chemical calculations is expected to be close to 7 Å. This is the ideal distance for effective polar aromatic interactions with a bound guest molecule. Binding a planar aromatic guest does therefore not require an energetically unfavorable rotation of the biphenyl units out of conjugation and should proceed effectively. In their low affinity-or OFF-states (*Z*-**1** and *E*-**2**) both tweezers project their biphenyl units in opposite directions and no chelating effect should be present, which is expected to reduce the binding affinity significantly.

### Switching properties

**Fig. 3:**
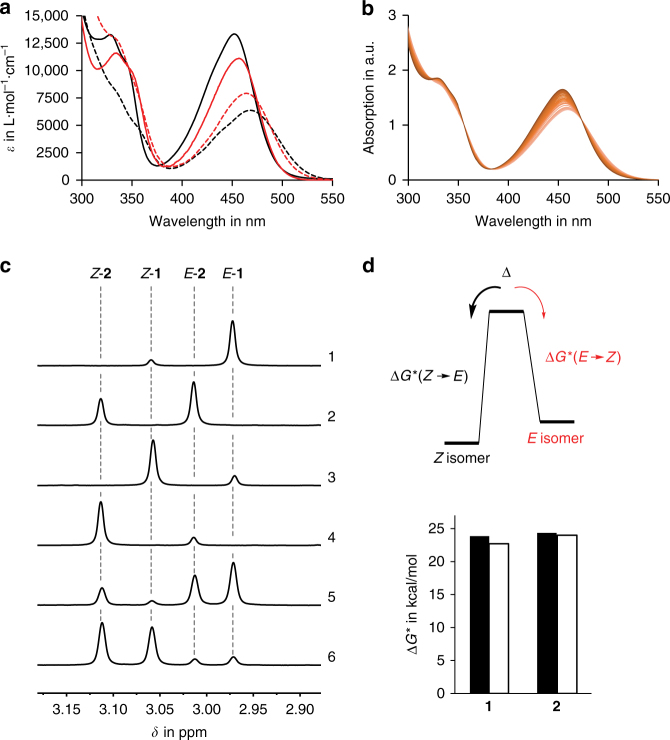
Switching behavior of HTI tweezers **1** and **2**. **a** Molar extinction coefficients of **1** (black) and **2** (red) in CHCl_3_. The *Z* isomers are shown in solid lines and *E* isomers in dashed lines. **b** Reversible absorption changes observed during irradiation of a 1:1 mixture of **1** and **2** in CHCl_3_ with 435 nm and 530 nm light. **c** Indicative section (signals of the methylene groups) of the ^1^H NMR spectra of **1** and **2** in CDCl_3_ solution at 27 °C after reaching the pss at two different wavelengths. 1. 86% *E*-**1** are obtained at 435 nm. 2. 63% *E*-**2** are obtained at 435 nm. 3. 80% *Z*-**1** are obtained at 530 nm. 4. 84% *Z*-**2** are obtained at 530 nm. 5. Essentially the same pss composition as for the individual tweezers is obtained for a 1:1 mixture of **1** and **2** at 435 nm. 6. Essentially the same pss composition as for the individual tweezers is obtained for a 1:1 mixture of **1** and **2** at 530 nm. **d** Free activation enthalpies for the thermal *E* to *Z* (solid bars) and *Z* to *E* (hollow bars) equilibrations of **1** and **2** in CDCl_3_ in the dark. Energy values range from 22.7 to 24.4 kcal mol^−1^

Both tweezers **1** and **2** exhibit very similar extinctions in their respective isomeric states (Fig. 3a and Supplementary Figs. 2-3) and therefore were expected to provide similar photoisomerization capacities. The photoswitching behavior of both HTIs **1** and **2** were investigated in CDCl_3_ solution and monitored by ^1^H NMR spectroscopy (Fig. 3c, Supplementary Fig. 4 and Supplementary Table 1). Irradiation was continued at different wavelengths until no further changes in the isomer composition were observed i.e., the photostationary state (pss) was reached. For both **1** and **2** the highest isomer ratios were obtained at the same wavelengths. At 435 nm the *Z* to *E* photoisomerization proceeds most efficiently resulting in high yields of the respective *E* isomers (86% of *E*-**1** and 63% of *E*-**2**). At 530 nm the opposite *E* to *Z* photoisomerization proceeds most effectively resulting in high yields of the respective *Z* isomers (80% of *Z*-**1** and 84% of *Z*-**2**). All other wavelengths tested led to lower isomer yields in each case. The photoisomerization experiments were repeated at −30 °C to exclude possible interference of the thermal *E* to *Z* isomerizations and gave very similar results (80% of *Z*-**1** and 82% of *Z*-**2**, see Supplementary Fig. 5). In a mixed solution of **1** and **2** in a 1:1 ratio slightly improved isomer compositions were obtained at 435 nm (88% of *E*-**1** and 64% of *E*-**2**) and 530 nm (82% of *Z*-**1** and 85% of *Z*-**2**) for both HTI tweezers, which demonstrates the simultaneous complementary photoswitching behavior (Fig. 3b, c, Supplementary Fig. 6, and Supplementary Table 2).

The thermal stability of the metastable *E* isomeric states were monitored in CDCl_3_ solutions in the dark (see Supplementary Figs. 7-8 and Supplementary Notes 2). Both tweezers do not convert completely to the *E* isomers thermally but rather an equilibrium state enriched in the *Z* isomers is reached. A first order kinetic analysis revealed the corresponding free energies of activation for the thermal *E* to *Z* isomerizations, as well as the *Z* to *E* isomerizations in each case (see Fig. 3d). These energies are in the 22.7 to 24.4 kcal mol^−1^ range, which results in conveniently high thermal stability of individual isomers and precludes interference of the dark reaction with the light signaling especially at lower temperatures.

### Binding of electron deficient aromatic guests

The binding constants and stoichiometries of photoresponsive molecular tweezers **1** and **2** were assessed by ^1^H NMR titration experiments^68,69^ in CDCl_3_ solution using 9-(dicyanomethylene)-2,4,7-trinitrofluorene (DTF, **10**) as electron poor aromatic guest. For the open tweezers forms *Z*-**1** and *E*-**2** no signal shifts were observed in the presence of the guest showing a complete absence of binding. Job plot analysis for the binding of **10** by the closed tweezer forms *E*-**1** and *Z*-**2** directly proved a 1:1 stoichiometry in each case (see Supplementary Fig. 9 for details). The high affinity form *E*-**1** binds **10** with a very high affinity of *K* = 12,000 L mol^−1^ in a 1:1 stoichiometry at −20 °C (Supplementary Figs. 10 and 11). The ^1^H NMR titrations were performed at this lower temperature to omit signal broadening occurring at 25 °C. Astonishingly, this observed binding affinity reaches the affinity strengths of much more rigidified and thus preorganized molecular tweezers^53,70^ in a host system with considerable degrees of freedom. The strongest signal shifts during binding are observed for the protons of the electron rich side arms, intended as recognition elements. As signals of both side arms are affected to similar extend in the presence of **10** a sandwich-type stacking arrangement of the aromatic units is the most plausible binding mode for the 1:1 stoichiometry found. The exclusive upfield shifts observed for the tweezers biphenyl arms strongly support this conclusion. Likewise the proton signals of the guest molecule (**10**) show exclusive and severe upfield shifts reflecting the ring current effects of the aromatic host surrounding (Supplementary Table 3). As different protons of **10** experience upfield shifts to varying degrees the mode of binding can be inferred. In the most plausible binding geometry the two neighboring protons at the mono-nitrated ring of **10** are bisecting the carbonyl group of *E*-**1**. This arrangement leads to severe upfield shifts of these signals (see below). Since polar aromatic interactions are not per se directional contributions of other binding modes are nevertheless likely.

For tweezers **2** in its high affinity *Z* form a binding constant of 2,300 L mol^−1^ for a 1:1 binding stoichiometry at −20 °C was found by the titration experiment (Supplementary Figs. 12 and 13 and Supplementary Table 4). As there are only polar aromatic interactions at play in **2** and these interactions are weakened by the polar solvent CDCl_3_ the binding constant found in this case is still remarkably strong.

It should be mentioned at this point that the observed binding for both high affinity forms *E*-**1** and *Z*-**2** are dynamic on the NMR time scale (signal shifts are observed upon binding not distinct signals for host, guest, and host-guest complex) and therefore fast exchange of guest molecules occurs in solution.

### Theoretical description

To better understand the observed molecular recognition we provide a theoretical description of the binding geometries at the B3LYP/6-311 G(d,p) level of theory for **1** and **2** in the open and the closed tweezers forms, as well as for the guest molecule DTF (**10**). For the complexes *E*-**1**^.^**10** and *Z*-**2**^.^**10** seven to ten different orientations of the guest **10** inside each *E*-**1** and *Z*-**2** were used as starting geometries obtained from a prior assembly search using the MM3* force field method with a Monte Carlo Multiple Minimum (MCMM) search algorithm and an energy threshold of 8 kJ mol^−1^. Further optimization of the obtained *E*-**1**^.^**10** and *Z*-**2**^.^**10** complexes was done on the B3LYP-GD3BJ/6-311 G(d,p) level of theory to account for significant contributions of dispersive interactions during binding. DFT optimization led to convergence of some structures resulting in four to five different binding geometries. We then calculated the corresponding ^1^H NMR shifts by averaging over the different binding geometries and found them generally in good agreement with the observed experimental chemical shifts. For details of the calculations see Supplementary Figs. 14-22. Supplementary Tables 5-14, Supplementary Data 1, Supplementary Methods, and Supplementary Note 3.

**Fig. 4:**
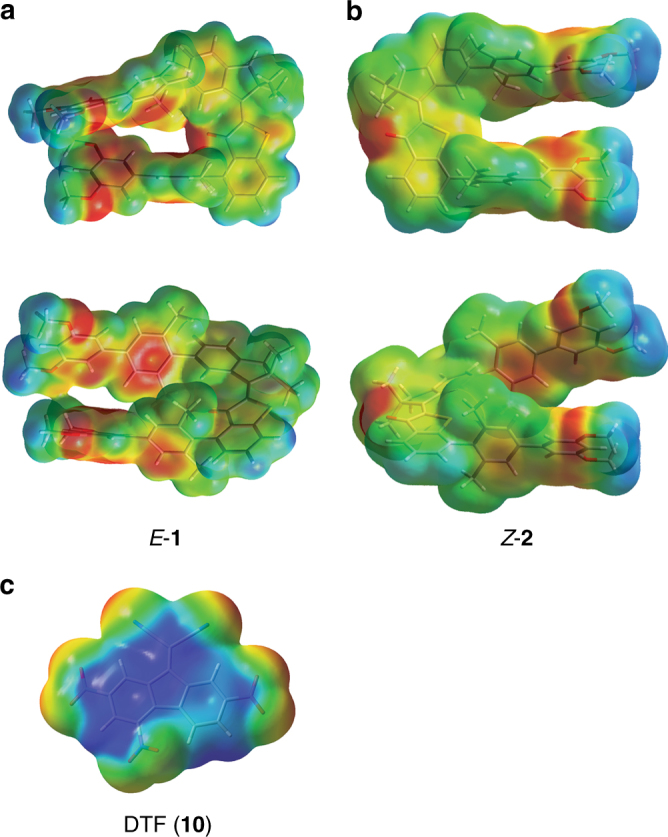
Molecular structures and ESPs of tweezers **1** and **2** and DTF. Calculations were performed at the B3LYP/6-311 G(d,p) level of theory. Areas highlighted in red signify high electron density and areas highlighted in blue signify low electron density. **a** Two different views of *E*-**1**. **b** Two different views of *Z*-**2**. **c** DTF guest (**10**)

**Fig. 5:**
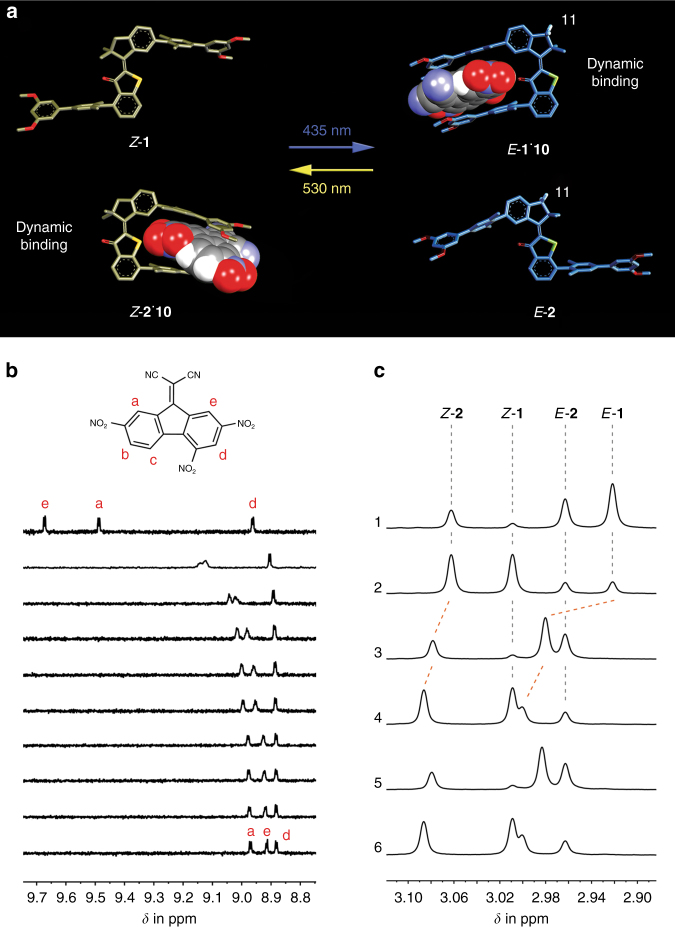
Simultaneous photoswitching controls dynamic guest relocalization. **a** The molecular structures of tweezers **1** and **2** in different isomeric states, as well as their supramolecular complexes *E*-**1**^.^**10** and *Z*-**2**^.^**10** were obtained from quantum chemical modeling at the B3LYP/6-311 G(d,p) or B3LYP-GD3BJ/6-311 G(d,p) level of theory, respectively. **b** Selected region of the ^1^H NMR (400 MHz, 253 K, CDCl_3_) spectra acquired during titration of **10** with *E*-**1**. Chemical shifts of the indicative protons of **10**, which change during the titration are assigned to the molecular structure of **10**. **c** Selected region of the ^1^H NMR (400 MHz, 293 K, CDCl_3_) spectra acquired during simultaneous complementary photoswitching and dynamic guest relocation. The isolated signals of proton 11 (marked in the molecular structures of *E*-**1**^.^**10** and *E*-**2** in Fig. 5a) are shown. All different molecular species are assigned. 1: Pss of the 1:1 mixture of **1** and **2** at 435 nm. 2: Pss of the 1:1 mixture of **1** and **2** at 530 nm. 3: Pss of the 1:1 mixture of **1** and **2** at 435 nm in the presence of 0.6 equiv. of **10**. 4: Pss of the 1:1 mixture of **1** and **2** at 530 nm in the presence of 0.6 equiv. of **10**. 5 and 6: Repetition of the dynamic guest relocation experiments at 435 nm and 530 nm

In Fig. 4 the optimized geometries for tweezers *E*-**1** and *Z*-**2**, as well as for the guest **10** are shown together with the corresponding electrostatic potentials (ESPs). Although dispersive interactions are also likely to be important in the free tweezers, the D3BJ geometry description of **1** and **2** was found to provide less agreement with the experimental ^1^H NMR shifts (see Supplementary Tables 5 and 6 and Supplementary Note 3). The optimized geometries for the complexes *E*-**1**^.^**10** and *Z*-**2**^.^**10** are shown in Fig. 5a. As can be seen from Fig. 4 the tweezers conformations appear to be highly preorganized and ideally suited to accommodate an electron deficient guest molecule. Because of the preorganizing geometry restrictions an open cavity is formed even prior to guest insertion, which should strongly facilitate the expected binding. Significant electron density is observed at the ends of both biphenyl arms in each case matching the electron deficiency observed for guest **10**. For *E*-**1** an additional electron rich region is found for the carbonyl moiety pointing inwards into the binding site. Comparison of the theoretical and experimental ^1^H NMR shifts shows that most probably a mixture of methoxy-group rotational isomers are present for *E*-**1** and *Z*-**2** in solution (see Supplementary Data 1 for all calculated geometries), the averaged theoretical values of the two energetically lowest structures gave good agreement with experimental chemical shifts.

To assess the binding complexes we have optimized 7 different arrangements of the guest molecule **10** inside each of the binding cavities of *E*-**1** and *Z*-**2** and found one clear global minimum in each case, as well as additional minima with low energies (see Fig. 5a for the global minimum complex structures and Supplementary Figs. 14-22, as well as Supplementary Tables 7-14 for the full analysis). In all *E*-**1**^.^**10** minima found the guest molecule is bound in a parallel stacked sandwich-type fashion between the electron rich tweezers arms (Supplementary Figs. 14-17). The global minimum is found for an arrangement in which the two neighboring protons in **10** (protons b and c in Fig. 5b) point directly at the electron rich carbonyl moiety of *E*-**1** in a CH-O hydrogen bond type interaction. This relative arrangement of host *E*-**1** to guest should lead to strong upfield shifts for guest signals e > c > a while protons b and d should only marginally be affected (Supplementary Tables 8 and 9). The comparison with experimental data (signals of **10** at a concentration of 0.26 mmol L^−1^ in the complex *E*-**1**^.^**10** at 4.2-fold excess of *E*-**1**) confirms this theoretical prediction very well (see Supplementary Table 8). In the experiment (see also Supplementary Tables 8 and 9) the strongest shift is observed for proton e (0.76 p.p.m.), closely followed by c, and then a. Proton b shows a less pronounced upfield shift while proton d is almost not shifted (0.08 p.p.m.). The agreement between experimental and theoretical ^1^H NMR spectra is improved significantly when chemical shifts of all calculated *E*-**1**^.^**10** minima structures are averaged (see Supplementary Tables 7-9).

A similar analysis was conducted for the host-guest complex of tweezers *Z*-**2** and **10** showing a somewhat different binding mode as global minimum (see Fig. 5a for the global minimum complex structure and Supplementary Figs. 18-22, as well as Supplementary Tables 11-14 for the full analysis). In this case the HTI chromophore is twisted out of planarity so that both biphenyl arms are not eclipsing each other anymore. However, a sandwich-type stacking arrangement is still found in which proton d is now pointing towards the tweezers sulfur atom. This predicted structure of the complex *Z*-**2**^.^**10** is found in good agreement with the experimental findings-especially with the chemical shift changes observed upon binding (see Supplementary Tables 11-14 for a comparison of experimental and theoretical values). Also in this case averaging of theoretically obtained ^1^H NMR chemical shifts leads to a significant improvement in the agreement of theory and experiment, especially when the guest signals are considered.

### Simultaneous complementary photoswitching powering dynamic guest relocation

In the following we demonstrate simultaneous complementary photoswitching that leads to a dynamic relocation of the guest molecules **10** from their binding equilibrium with tweezers *Z*-**2** to a new binding equilibrium with tweezers *E*-**1** and back (Fig. 5, Supplementary Fig. 23, and Supplementary Note 4). A 1:1 mixture of both tweezers was prepared first and irradiated with 435 nm and 530 nm until the respective pss was reached. Afterwards 0.6 equiv. of the guest molecule **10** were added and the solution was again irradiated with the two wavelengths until the respective pss was reached. We used again the ^1^H NMR signal of the tweezers proton 11 (see Fig. 5a for assignment of proton 11 to the structures), which appears in a non-overlapping region of the spectrum and is significantly different for each isomer of tweezers **1** and **2** (Fig. 5c), to monitor the effects of irradiation on the guest location. This signal shifts significantly downfield upon binding in *Z*-**2** and *E*-**1** but is not at all changed for the non-binding isomers *E*-**2** and *Z*-**1**.

At 435 nm irradiation two species with high affinity to the guest molecule are present in solution: 1.4 equiv. of *E*-**1** (i.e., 88% *E*-**1** in the pss of the **1** and **2** mixture) and 0.6 equiv. of *Z*-**2** (64% *E*-2 in the pss of the **1** and **2** mixture) as compared to 1.0 equiv. of guest **10**. Since *E*-**1** is predominant and also possesses a significantly higher affinity as compared to *Z*-**2** the guest molecule **10** is primarily bound by tweezers *E*-**1** at this wavelength. A very strong downfield shift of the proton 11 signal of tweezers *E*-**1** provide direct evidence for this binding assessment (Fig. 5c, spectrum 3).

At 530 nm the relative ratio of high affinity species is reversed: 0.3 equiv. of *E*-**1** (82% *Z*-**1** in the pss of the **1** and **2** mixture) and 1.4 equiv. of *Z*-**2** (85% *Z*-**2** in the pss of the **1** and **2** mixture) as compared to 1.0 equiv. of guest **10**. Although *E*-**1** possesses a significantly higher binding affinity as compared to *Z*-**2** it could at most prevent 0.3 equiv. of guest **10** from the dynamic relocation at this wavelength. Therefore, at least 70% of the guest molecules **10** are effectively relocated and now preferably bound in tweezers *Z*-**2** as can also be seen by the increased downfield shift of their proton 11 signal (Fig. 5c spectrum 4 vs. spectrum 3). A second relocation cycle gave exactly the same result proving full reversibility of the process.

## Discussion

In this work we present a new type of photoresponsive molecular tweezers whose strong affinity for electron deficient aromatic guest molecules can be controlled reversibly by visible (blue and green) light irradiation. Pairing two such tweezers with complementary molecular setup in the same solution we have demonstrated the feasibility of reversible simultanous complementary photoswitching. In this way a single light signal orchestrates four supramolecularly connected processes (opening of the first tweezers, loss of their guest binding equilibrium, closing of the second tweezers, and establishment of their new binding equilibrium with the guest) at the same time in solution. The realization of this concept allowed us to control a complex supramolecular relocation behavior by drastically reduced outside signaling. We believe that such functional coupling of different responsive elements, which react to the same outside signals but in distinct individual ways provides a highly interesting possibility for generating complex behavior at the molecular scale. If molecular systems are set up in distinct ways to react differently to the same signals, the level of control is only limited by the number of functions that can be implemented in these systems. Here we show such complementary molecular setup with two distinct molecules but the number of binding sites could in principle be increased per molecule, different types of molecular recognition can be invoked, and further functionalities such as catalytic moieties etc. are employable. Paired with e.g., orthogonal photoswitching this approach could develop into a powerful means to precisely influence multicomponent molecular and supramolecular systems and for the generation of complex triggered behavior and functions in the future.

## Methods

### General

The synthesis and spectroscopic characterization of molecular tweezers **1** and **2** and synthetic intermediates is given in the Supplementary Methods. The conformational analysis of different isomeric forms of **1** and **2** in solution is provided in Supplementary Note 1. The quantitative analysis of the thermal isomerization behavior in the dark is given in Supplementary Note 2. A comprehensive theoretical description of the molecular geometries of **1** and **2**, as well as their host-guest complexes with **10** is presented in Supplementary Note 3. An analysis of the photocontrolled dynamic guest relocation is given in the Supplementary Note 4.

### Extinction coefficients of *Z* and *E* isomers

The Ultraviolet/vis absorption spectra of pure *E* and *Z* isomers of **1** and **2** were obtained by subtraction of a *E*/*Z*-mixture spectrum with known composition (previously determined by integration from ^1^H NMR spectroscopy) from a second *E*/*Z*-mixture spectrum with different but also known isomer composition and a subsequent multiplication with an upkeep factor. Weighting is done by multiplying the first *E*/*Z*-mixture spectrum with the *Z* (or *E*) isomer percentages of the second *E*/*Z*-mixture and vice versa. The obtained absorption spectrum of the respective pure isomer is multiplied by a compensation factor to match the absorption values of the isosbestic points. Extinction coefficients were determined by charging a 50 mL volumetric flask with a defined mass (2.287 mg for **1** and 2.707 mg for **2**) of the chromophore (weighted on a *Sartorius Cubis*® MSE2.7 S balance) and CHCl_3_ (spectroscopic grade) to obtain a solution with defined concentration. From the known positions of the isosbestic points and the previously determined Ultraviolet/vis spectra of the pure isomers, the extinction coefficients could be determined using the Lambert-Beer law.

### Photoisomerization of 1 and 2 under continuous irradiation

To obtain the isomer composition in the different photostationary states (pss) of **1** and **2**, 5 mM solutions (CDCl_3_) of each tweezers were prepared in two NMR tubes. The solutions were irradiated at 435 nm for *Z*/*E* isomerization and at 530 nm for *E*/*Z* isomerization. ^1^H NMR spectra were recorded after defined time intervals. For *Z*/*E* isomerization the new signals of the *E* isomer were integrated and compared to the signals of remaining *Z* isomer to calculate the percentage of *E* isomer after irradiation. To obtain the isomer composition in the pss irradiation was continued until no further change in the composition of the two isomers was observed. The *E*/*Z* photoisomerization was conducted in a similar way. In this case high *Z* isomer yields are obtained despite only low extinctions of the *E* isomers at 530 nm, which is most likely the result of the absence of *Z* isomer absorption at this wavelength and a reasonable quantum yield for this photoreaction. It should also be noted that the LEDs used for irradiation are not completely monochrome and probably tail out into regions of higher absorption of the *E* isomers.

### Photoisomerization of 1 and 2 at −30 °C

Since the *E*/*Z* photoisomerizations of **1** and **2** proceed in a similar time regime as their thermal induced *E*/*Z* isomerisation at 25 °C irradiation of the *E* enriched solutions was repeated at −30 °C. A sample of each tweezers **1** and **2**, with a concentration of 0.26 mM, was prepared in a NMR tube. Each sample was irradiated continuously with a 530 nm LED at −30 °C. ^1^H NMR spectra were recorded in 30 min time intervals and *E*/*Z* ratios were determined by integration of indicative signals. As *E*-**1** and *E*-**2** possess a thermal half life of *τ*_1/2_(*E*-**1**) = 414 d and τ_1/2_(*E*-**2**) = 16.7 a at −30 °C, a thermal process within the 2 h of irradiation could be excluded. A stable isomer ratio in the pss could be established after 2 h in each case (**1**: 82% *Z* and 18% *E* isomer; **2**: 80% *Z* and 20% *E* isomer), which closely resembles the pss composition at 25 °C.

### Photostationary states of a mixture of 1 and 2

For the determination of the isomer composition in the pss of both tweezers together in solution, a 1:1 mixture of **1** and **2** in CDCl_3_ (conc. = 2.2 mm for each tweezers) was prepared in a NMR tube. The solution was irradiated at 435 nm for *Z*/*E* isomerization or 530 nm for *E*/*Z* isomerization. ^1^H NMR spectra were recorded after defined time intervals. To obtain the isomer composition in the pss irradiation was continued until no further change in the composition of the isomers was observed. The *E*/*Z* photoisomerization was conducted in a similar way.

### Binding properties of *E*-1

Two stock solutions were prepared for titration experiments. A 0.26 mm stock solution A of **10** in CDCl_3_ was prepared. A defined volume of this solution was transferred to another vial containing solid **1**, resulting in a mixed solution of **1** (5 mm) and **10** (0.26 mm) in CDCl_3_. This stock solution B contained tweezers **1** in an isomeric mixture. To obtain a maximum amount of the binding *E* isomer the solution was irradiation at 435 nm. 71% of the binding *E* isomer was obtained (note that the *E* isomer content in the pss is lowered at the higher 5 mM concentration), leading to a final concentration of 3.5 mm for *E*-**1** and 0.26 mm for **10** in stock solution B. Two NMR tubes were loaded with 700 μL of either stock solution A or B. Each NMR tube was titrated with the respective other stock solution in steps of 75 μL additions. After each addition the solutions were carefully mixed and a ^1^H NMR spectrum was recorded (253 K) to determine the chemical shift changes of indicative protons of **10** (see Supplementary Table 3 and Supplementary Figure 10). A total of 4 × 75 μL were added to each NMR tube.

### Binding properties of *Z*-2

Two stock solutions were prepared for titration experiments. A 0.25 mM stock solution A of **10** in CDCl_3_ was prepared. A defined volume of this solution was transferred to another vial containing solid **2**, resulting in a mixed solution of **2** (5 mm) and **10** (0.25 mm) in CDCl_3_. This stock solution B contained tweezers **2** in an isomeric mixture. To obtain a maximum amount of the binding *Z* isomer the solution was irradiation at 530 nm. 52% of the binding *Z* isomer was obtained, leading to a final concentration of 2.6 mm for *Z*-**2** and 0.25 mm for **10** in stock solution B. Two NMR tubes were loaded with 700 μL of either stock solution A or B. Each NMR tube was titrated with the respective other stock solution in steps of 75 μL additions. After each addition the solutions were carefully mixed and a ^1^H NMR spectrum was recorded (253 K) to determine the chemical shift changes of indicative protons of **10** (see Supplementary Table 4 and Supplementary Fig. 12). A total of 5 × 75 μL were added to each NMR tube.

### Data availability

All data that support the findings of this study are available from the corresponding author upon reasonable request. The X-ray crystallographic coordinates for the structure of *E*-**5** reported in this study have been deposited at the Cambridge Crystallographic Data Centre (CCDC), under CCDC number 1578150. These data can be obtained free of charge from the Cambridge Crystallographic Data Centre via www.ccdc.cam.ac.uk/data_request/cif.

## Electronic supplementary material

Supplementary Information

Description of Additional Supplementary Files

Supplementary Data 1

Supplementary Movie 1
