# Supplementary material for: Simultaneous complementary photoswitching of hemithioindigo tweezers for dynamic guest relocalization
Source: Nat Commun. 2018 Apr 13;9:1456. doi: 10.1038/s41467-018-03912-7 (PMC5899155; doi:10.1038/s41467-018-03912-7)
Supplement: Supplementary file 1 — Supplementary Information [file 41467_2018_3912_MOESM1_ESM.pdf]

Supplementary Information

Simultaneous Complementary Photoswitching of Hemithioindigo Tweezers for Dynamic Guest Relocalization

S.Wiedbrauk et. al

Supplementary Figures

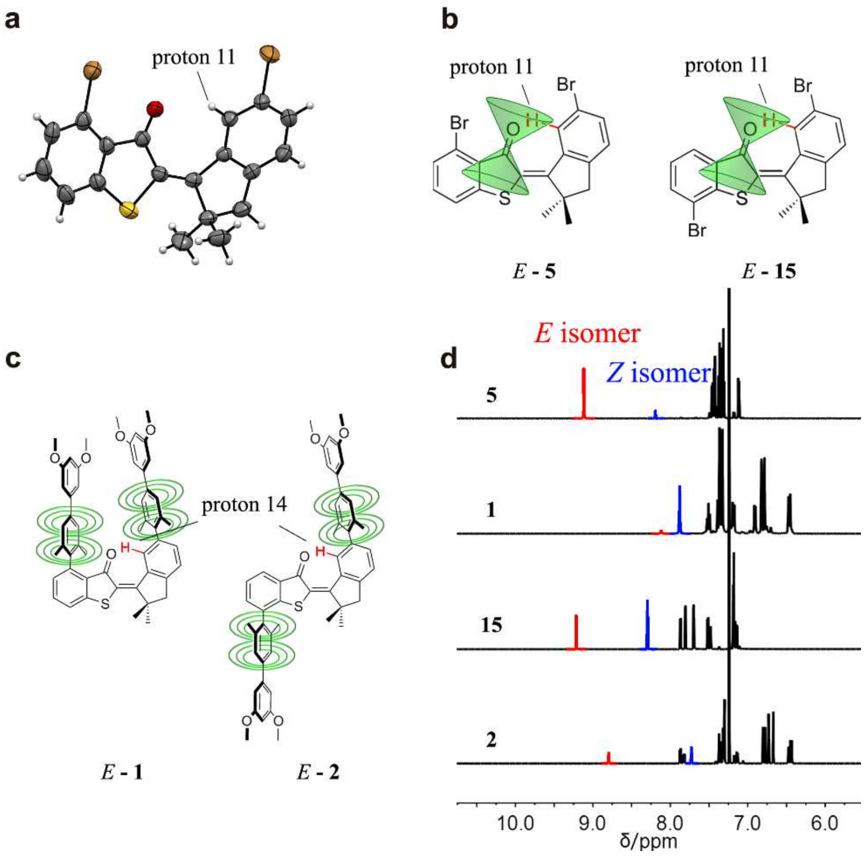

Supplementary Figure 1 | Conformational analysis of tweezers 1 and 2. Signal assignments to the different isomeric forms were made by comparison with precursor HTIs **5** (for tweezers **1**) and **15** (for tweezers **2**). a) Structure of **5** (*E* isomer) in the crystalline state with proton 11 assigned. b) Magnetic anisotropic effect of the carbonyl group.[1] Signals of protons within the cone appear strongly downfield shifted. c) In tweezers **1** and **2** the combined magnetic anisotropic/ring current effects of the carbonyl group and the aromatic biphenyl arms affect the chemical shift of the corresponding proton 14 in the ^{1}H NMR spectrum. In *E*-**1** the opposite effects (downfield shifting carbonyl group and upfield shifting biphenyl ring currents) are counterbalanced most strongly. d) Aromatic part of the ^{1}H NMR (**1**: 400 MHz, 295 K; **2**, **5**: 600 MHz, 300 K; **15**: 800 MHz, 300 K; $CDCl_3$) spectra of **5**, **1**, **15**, and **2**, which are present as *Z* and *E* isomer mixtures. The assigned signals of indicative protons 11 or 14 for different isomers (*Z* in blue and *E* in red) are highlighted.

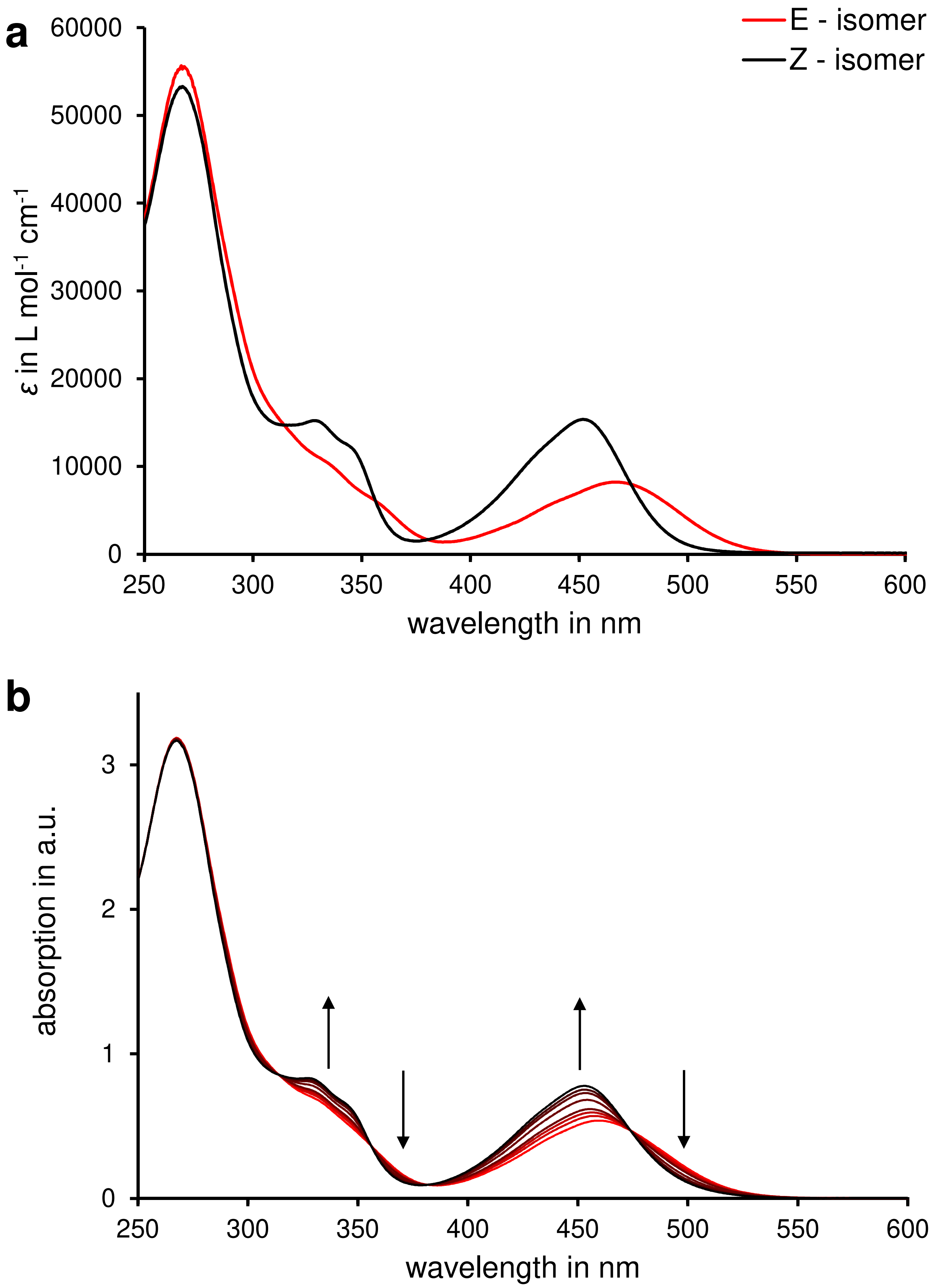

Supplementary Figure 2 | Extinction and photoswitching of 1. a) Extinction coefficients of **1** in $CHCl_3$ (*Z* isomer black, *E* isomer red). b) Absorption changes during *E*/*Z* photoisomerization. Increasing and decreasing absorptions are indicated by black arrows. Distinct isosbestic points are observed at 316 nm, 357 nm, 381 nm, and 475 nm.

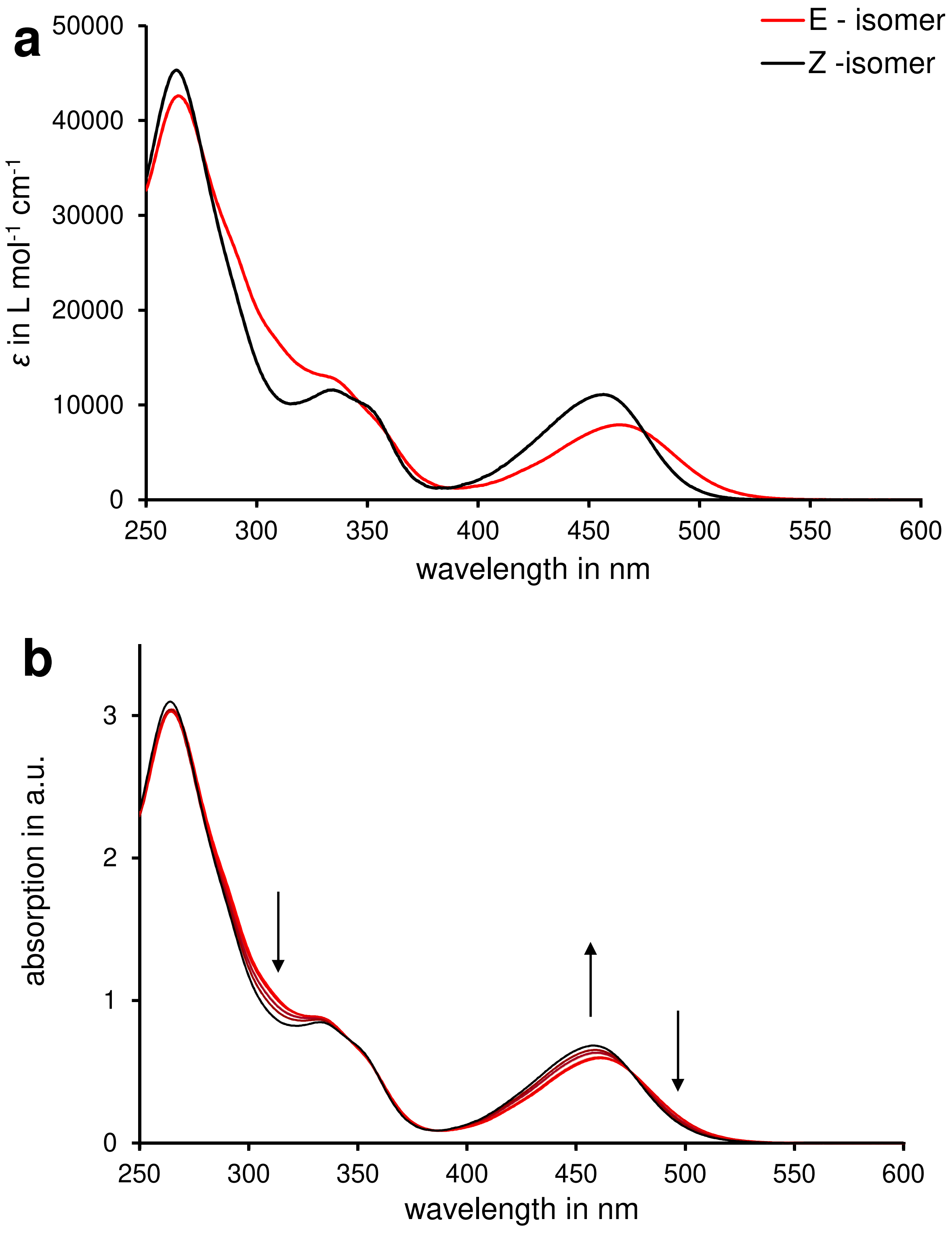

Supplementary Figure 3 | Extinction and photoswitching of 2. a) Extinction coefficients of **2** in $CHCl_3$ (*Z* isomer black, *E* isomer red). b) Absorption changes during *E*/*Z* photoisomerization. Increasing and decreasing absorptions are indicated by black arrows. Distinct isosbestic points are observed at 276 nm, 347 nm, 360 nm, 386 nm, and 476 nm.

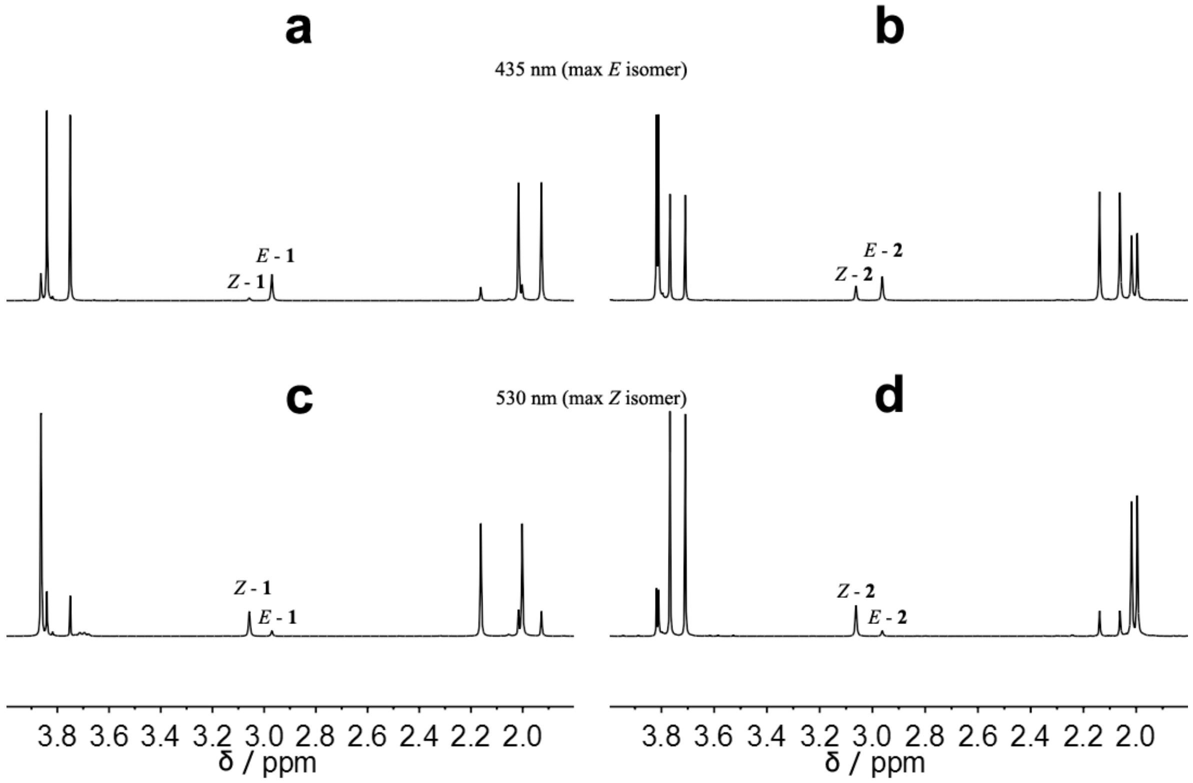

Supplementary Figure 4 | Isomer composition of 1 and 2 in the pss. ^{1}H NMR (400 MHz, 293 K, $CDCl_3$) spectra for the determination of the isomer composition in the pss at different wavelengths of irradiation. Indicative signals for each isomer of tweezers **1** and **2** are specified. a) Pss of **1** at 435 nm. b) Pss of **2** at 435 nm. c) Pss of **1** at 530 nm. d) Pss of **2** at 530 nm.

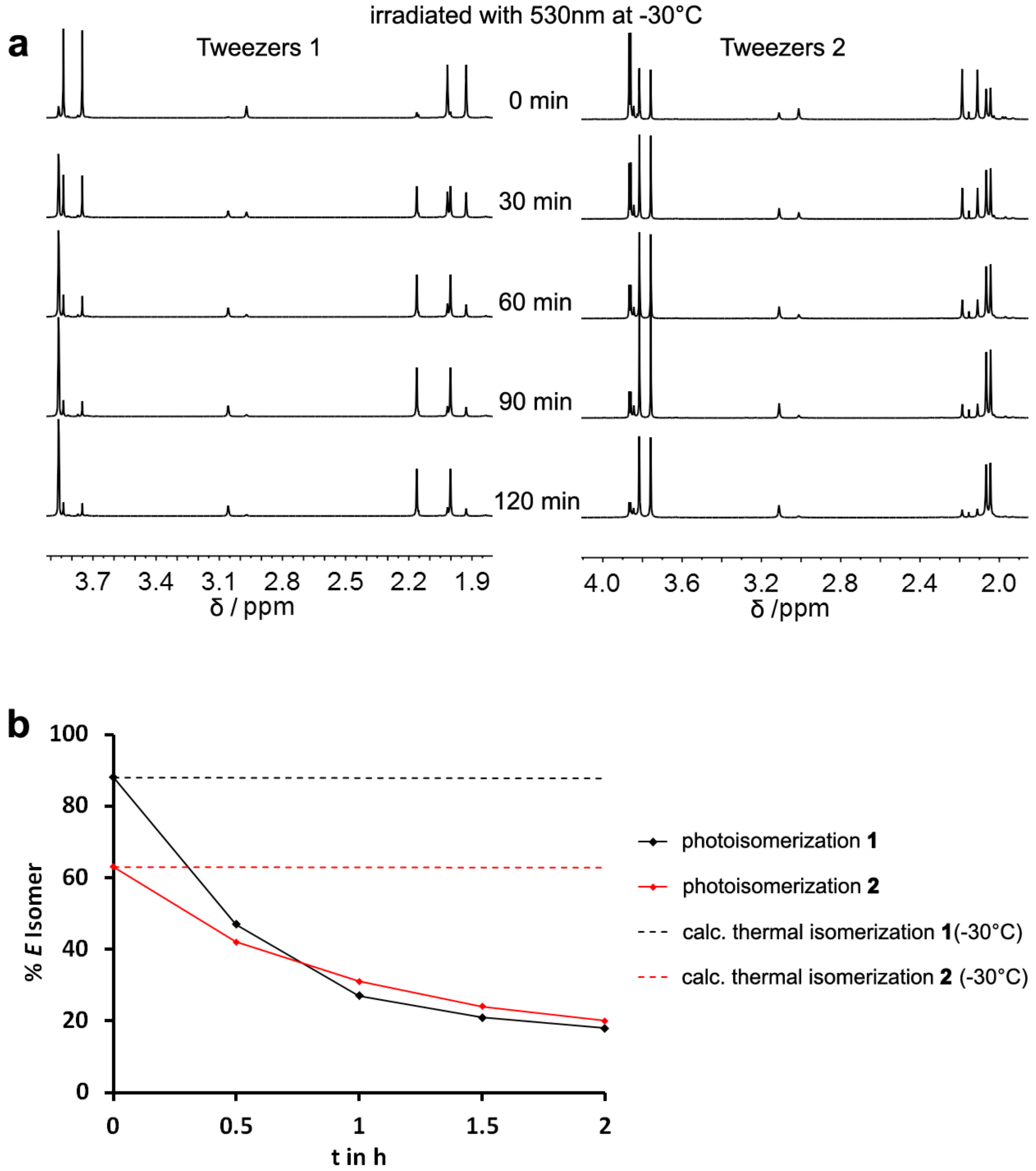

Supplementary Figure 5 | *E*/*Z* photoisomerization of 1 and 2 at -30°C. a) ^{1}H NMR spectra (400 MHz, 293 K, $CDCl_3$) of **1** and **2** after continuous irradiation with a 530 nm LED recorded in time intervals of 30 min. b) Decrease of the *E* isomers of **1** (black) and **2** (red) over time at continuous irradiation with a 530 nm LED at -30°C. Data points were obtained from integration of indicative ^{1}H NMR signals. Dashed lines indicate the calculated thermal decrease of *E*-**1** and *E*-**2** at -30 °C.

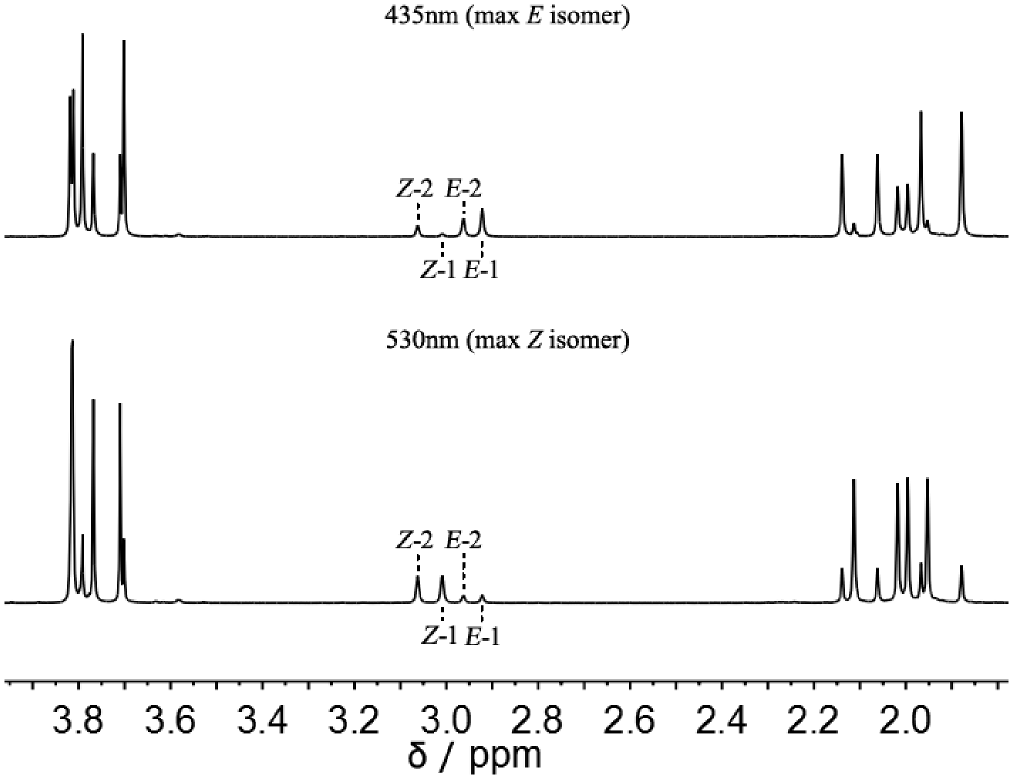

Supplementary Figure 6 | Isomer composition of a mixture of 1 and 2 in the pss. ^{1}H NMR (400 MHz, 293 K, $CDCl_3$) spectra for the determination of the isomer composition in the pss for a 1:1 mixture of **1** and **2**. Isomer composition in the pss at 435 nm (top) and at 530 nm (bottom).

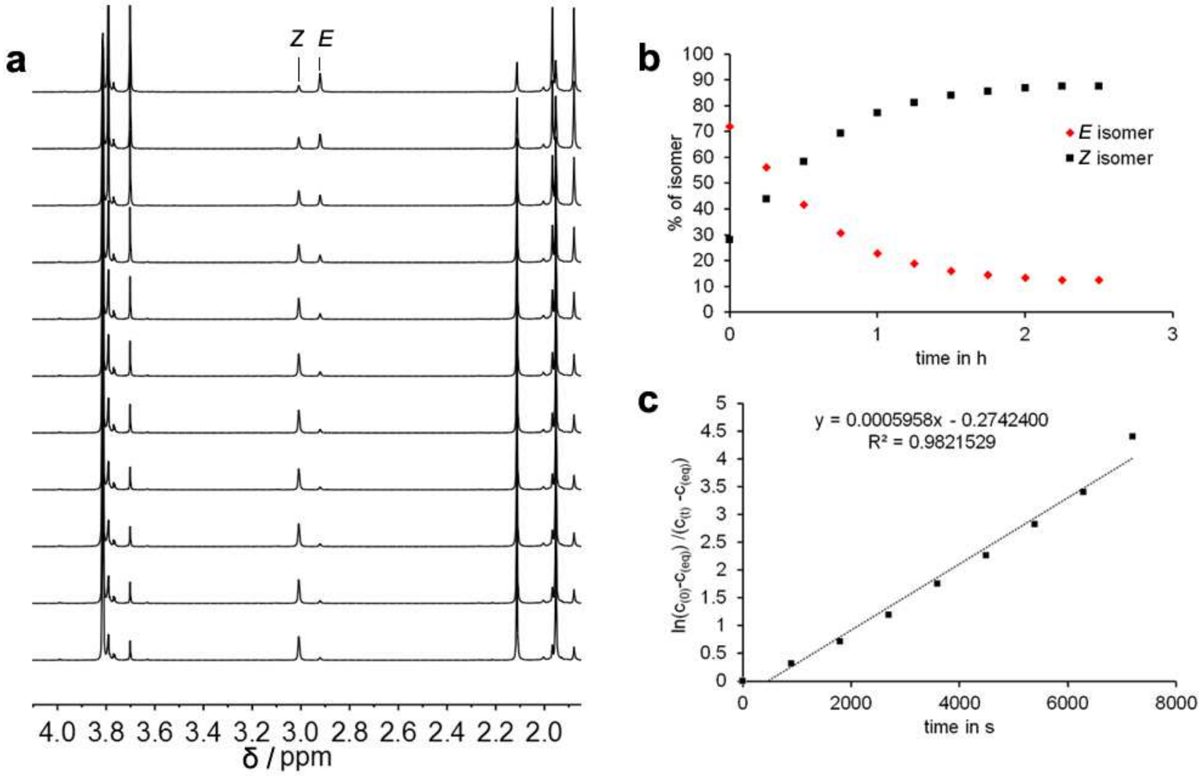

Supplementary Figure 7 | Thermal *E*/*Z* isomerization of 1 in the dark. At elevated temperatures the *E* isomer of molecular tweezers **1** is interconverted into the *Z* isomer. a) ^{1}H NMR (400 MHz, 293 K, $CDCl_3$) spectra acquired during thermal conversion of *E*-**1** to *Z*-**1** in the dark at 35 °C. b) Decrease of the *E* isomer and increase of the *Z* isomer over time in the dark. Data points were obtained from integration of indicative ^{1}H NMR signals. c) A first order kinetic analysis taking into account the dynamic equilibrium gives a linear relationship. Its slope *m* can be translated into the rate constant $k(E{\rightarrow}Z)$ of the thermal *E*/*Z* isomerization according to eq. 2 (Supplementary Notes 2).

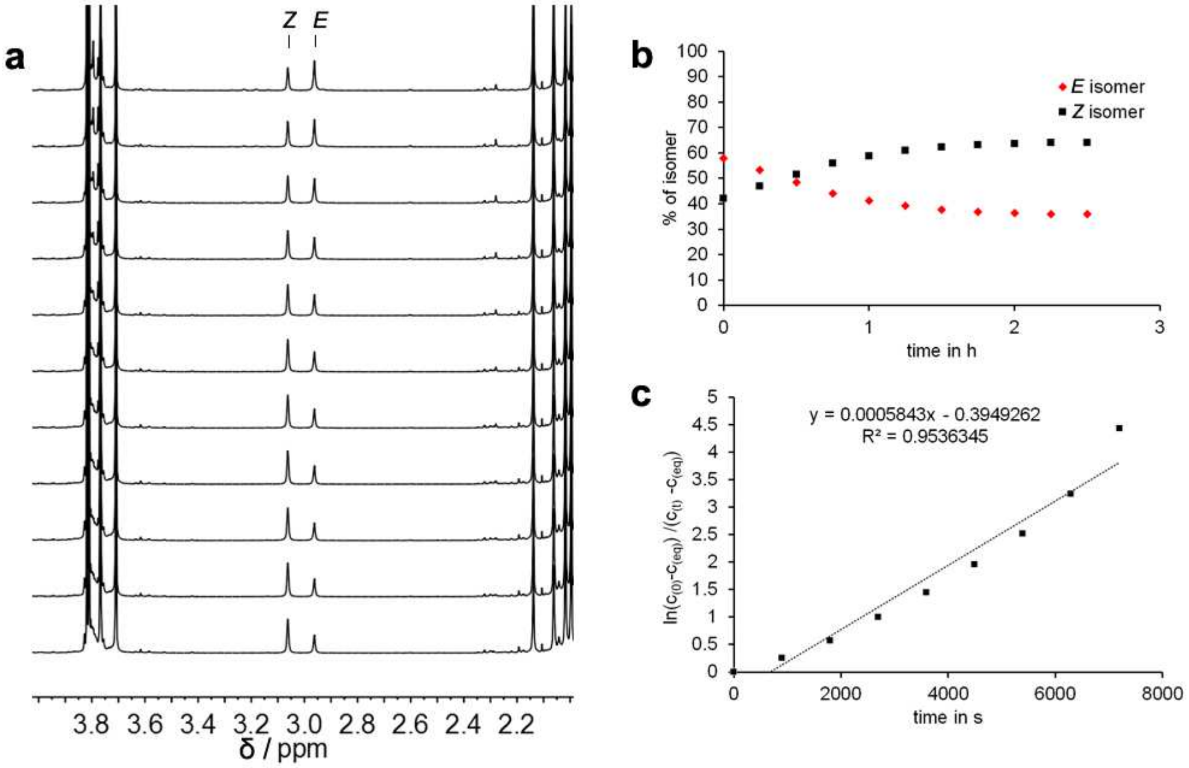

Supplementary Figure 8 | Thermal *E*/*Z* isomerization of 2 in the dark. At elevated temperatures the *E* isomer of molecular tweezers **2** is interconverted into the *Z* isomer. a) ^{1}H NMR (400 MHz, 293 K, $CDCl_3$) spectra acquired during thermal conversion of *E*-**2** to *Z*-**2** in the dark at 50 °C. b) Decrease of the *E* isomer and increase of the *Z* isomer over time in the dark. Data points were obtained from integration of indicative ^{1}H NMR signals. c) A first order kinetic analysis taking into account the dynamic equilibrium gives a linear relationship. Its slope *m* can be translated into the rate constant *k*(*E*→*Z*) of the thermal *E*/ *Z* isomerization according to eq. 2 (Supplementary Notes 2).

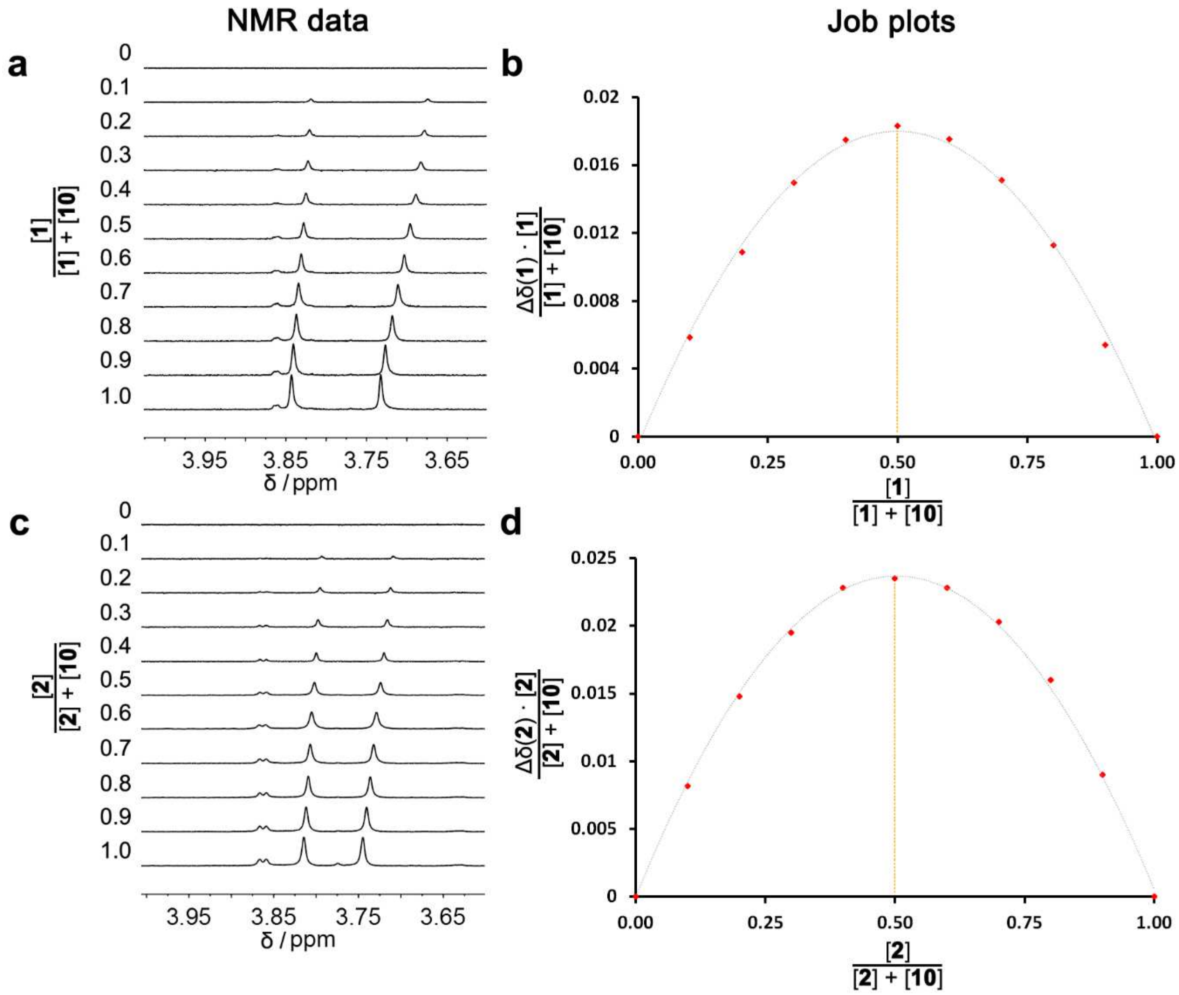

Supplementary Figure 9 | Determination of binding stoichiometries of *E*-1 and *Z*-2 with 10. a) Selected region of ^{1}H NMR spectra (400 MHz, 253 K, $CDCl_3$) showing the methoxy group signals of **1** at varying ratios (from 0 to 1 in 0.1 steps) of *E*-**1**:**10** at a constant total concentration of 0.26 mM. b) Job plot analysis of the binding of *E*-**1** with **10**. The maximum at 0.5 confirms a 1:1 stoichiometry. c) Selected region of spectra ^{1}H NMR (400 MHz, 253 K, $CDCl_3$) showing the methoxy group signals of **2** at varying ratios (from 0 to 1 in 0.1 steps) of *Z*-**2**:**10** at a constant total concentration of 0.26 mM. d) Job plot analysis of the binding of *Z*-**2** with **10**. The maximum at 0.5 confirms a 1:1 stoichiometry.

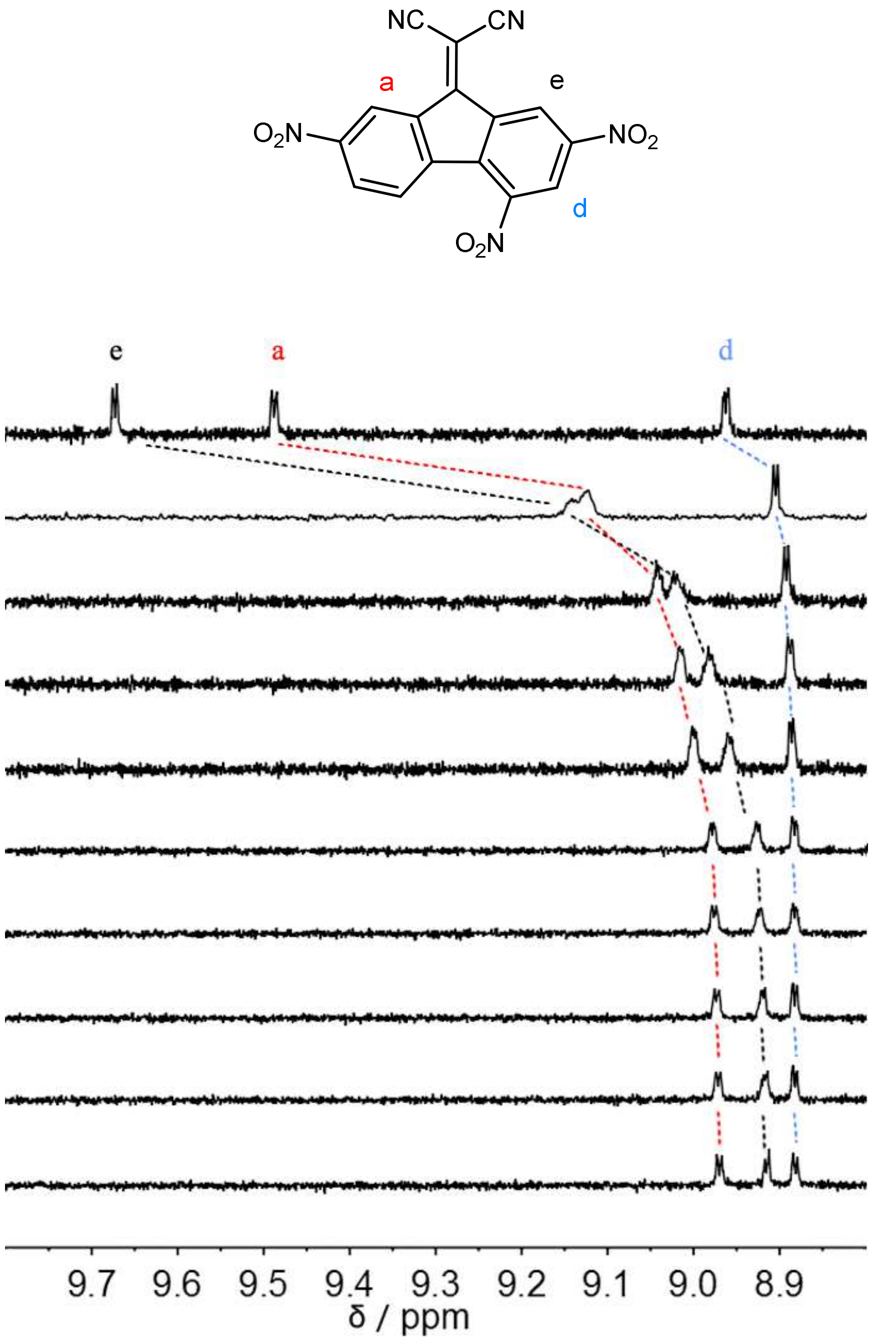

Supplementary Figure 10 | Titration of 10 with *E*-1. Selected region of the ^1H NMR (400 MHz, 253 K, $CDCl_3$) spectra acquired during titration of **10** with *E*-**1**. Chemical shifts of the indicative protons of **10**, which change during the titration, are assigned to the molecular structure of **10** and are color coded.

Details

Time to fit	0.2131 s
SSR	4.8556e-5
Fitted datapoints	30
Fitted params	4

Parameters

Parameter (bounds)	Optimised	Error	Initial
K (0 → ∞)	12420.75 M^{-1}	± 1.2437 %	100.00 M^{-1}

Back Next

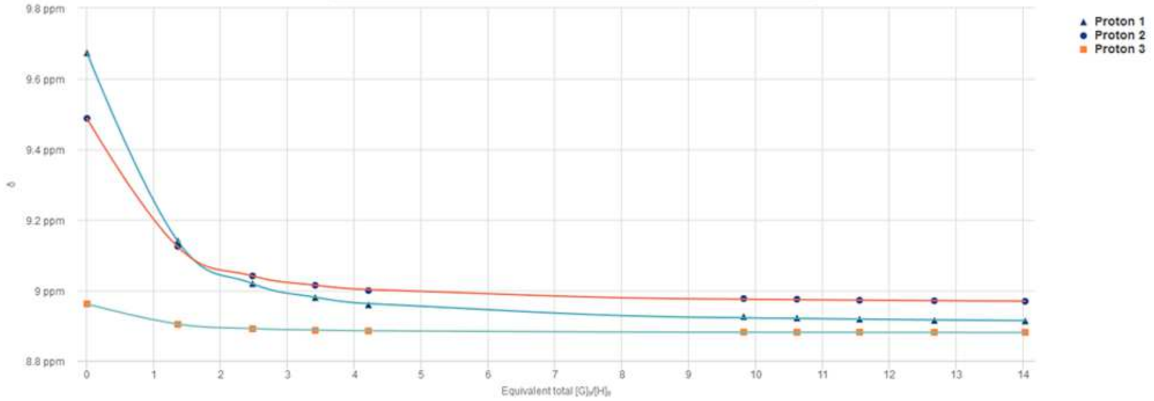

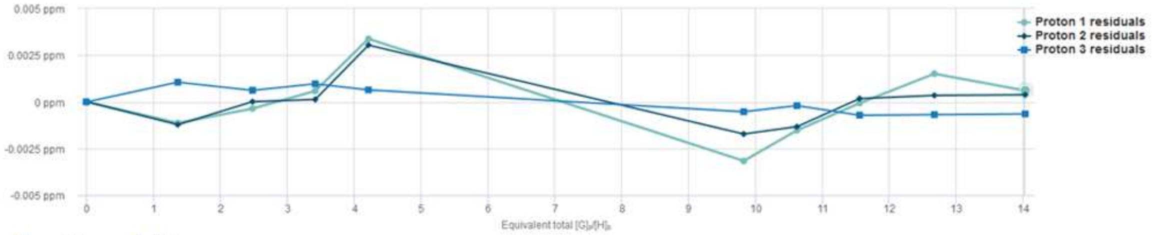

Quality of fit

Fit	RMS	Covariance
Proton 1	1.6788e-3	5.7069e-5
Proton 2	1.2571e-3	6.8653e-5
Proton 3	6.7587e-4	8.1421e-4
**Total**	**1.2722e-3**	**2.0348e-5**

Coefficients

Fit	H	HG
Proton 1	9.6735	8.8978
Proton 2	9.4879	8.9584
Proton 3	8.9626	8.8797

Supplementary Figure 11 | Determination of the binding constant for complex *E*-1·10. Fitting of the titration data of **10** with *E*-**1** as host in a 1:1 stoichiometry. Other stoichiometries showed negative errors (2:1) and/or were not reasonable structurally based on binding geometry analysis (1:2). Screenshot of the BindFit webpage[2].

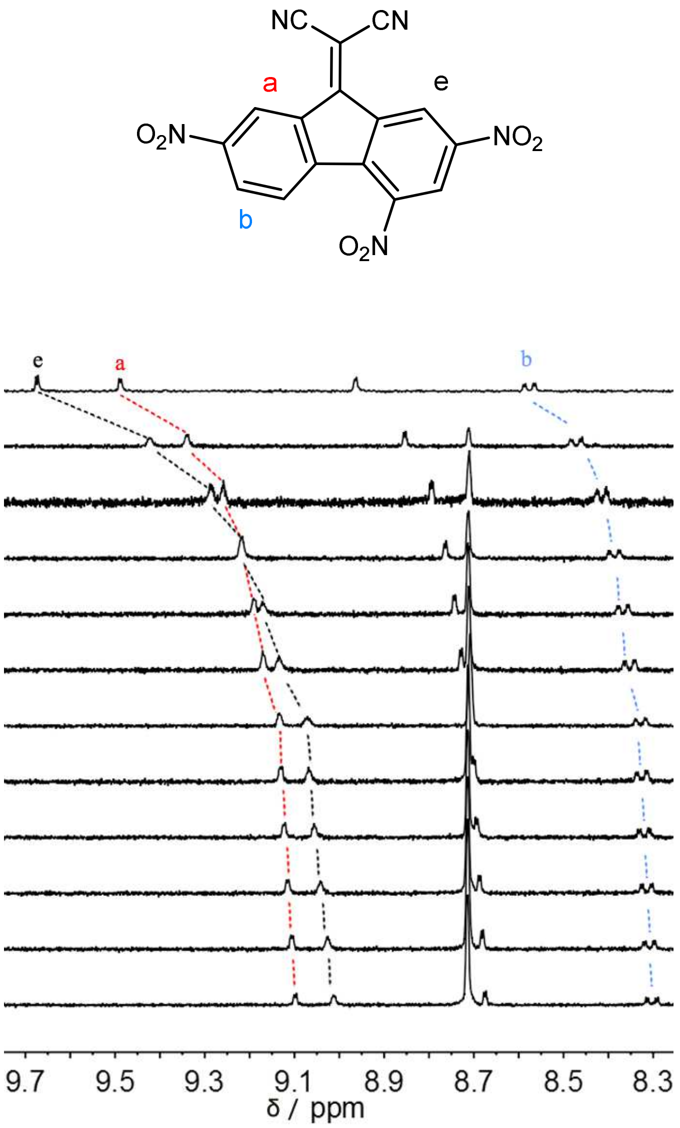

Supplementary Figure 12 | Titration of 10 with *Z*-2. Selected region of the ^{1}H NMR (400 MHz, 253 K, $CDCl_3$) spectra acquired during titration of **10** with *Z*-**2**. Chemical shifts of the indicative protons of **10**, which change during the titration, are assigned to the molecular structure of **10** and are color coded.

Details

Time to fit	0.2193 s
SSR	1.4150e-3
Fitted datapoints	36
Fitted params	4

Parameters

Parameter (bounds)	Optimised	Error	Initial
K (0 → ∞)	2376.70 M^{-1}	± 2.5207 %	100.00 M^{-1}

Back Next

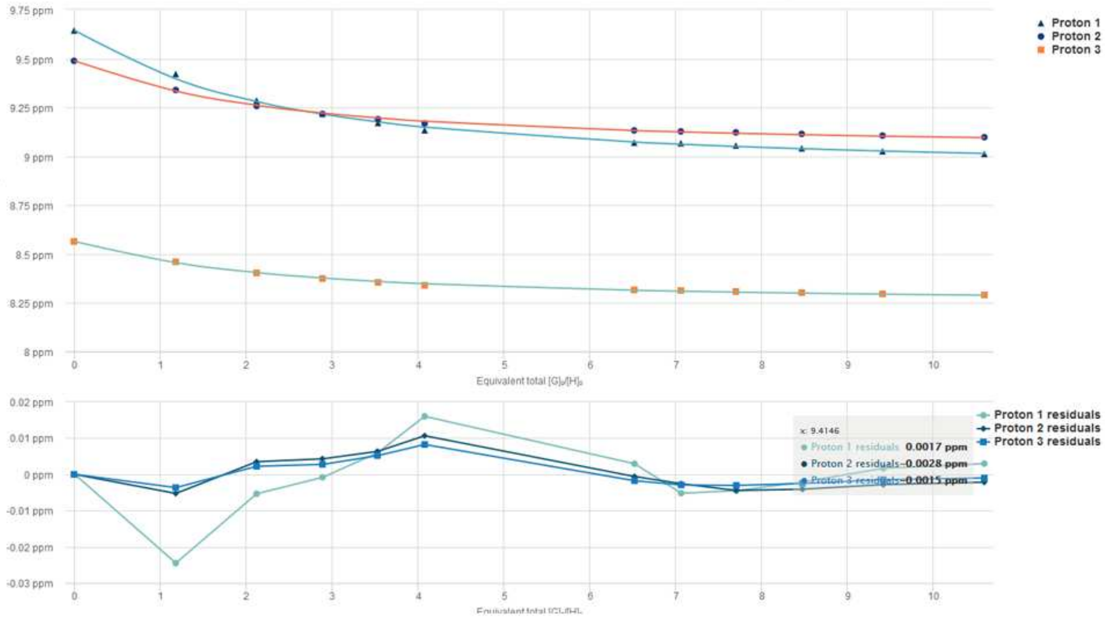

Quality of fit

Fit	RMS	Covariance
Proton 1	9.1048e-3	2.4574e-3
Proton 2	4.7318e-3	1.7962e-3
Proton 3	3.5540e-3	2.0721e-3
**Total**	**6.2695e-3**	**1.3518e-3**

Coefficients

Fit	H	HG
Proton 1	9.6440	8.9079
Proton 2	9.4886	9.0291
Proton 3	8.5651	8.2437

Supplementary Figure 13 | Determination of the binding constant for complex *Z*-2·10. Fitting of the titration data of **10** with *Z*-**2** as host in a 1:1 stoichiometry. Other stoichiometries (1:2 and 2:1) resulted in much higher errors of the fit and were therefore dismissed as implausible. Screenshot of the BindFit webpage[2].

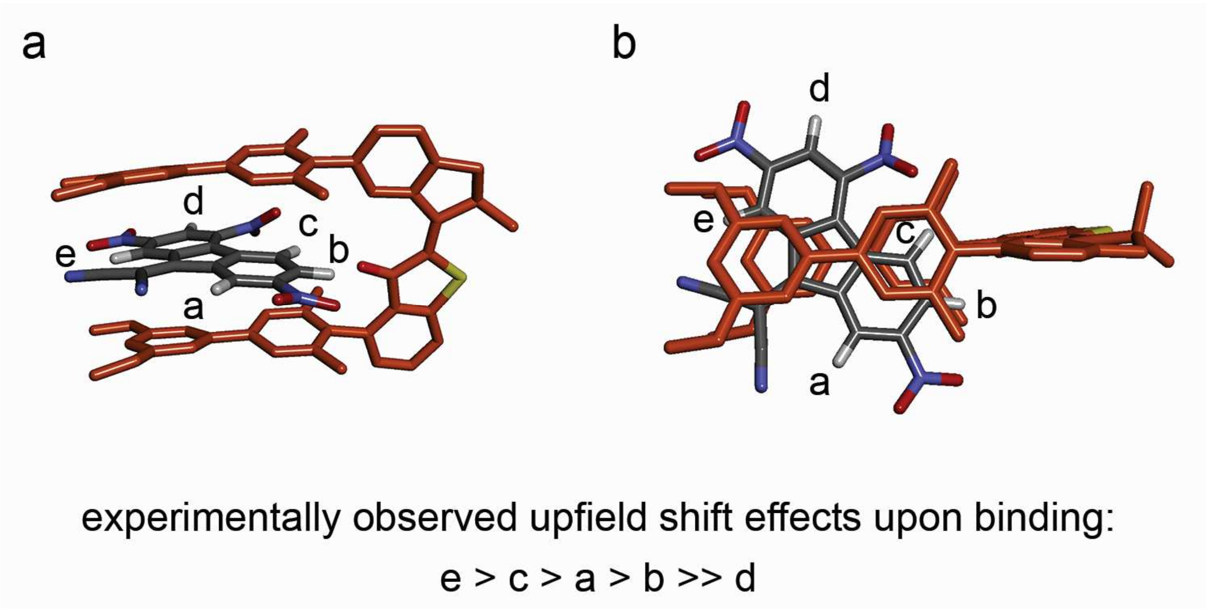

Supplementary Figure 14 | Molecular structure of the host-guest complex A (*E*-1·10). See also the corresponding data of complex **A** (lowest DFT energy) in Supplementary Table 7. Calculations were performed at the B3LYP-GD3BJ/6-311G(d,p) level of theory. a) Side view. b) Top view. The guests proton signals a-d are indicated. Experimentally observed relative upfield-shift effects on the guest-proton signals are given.

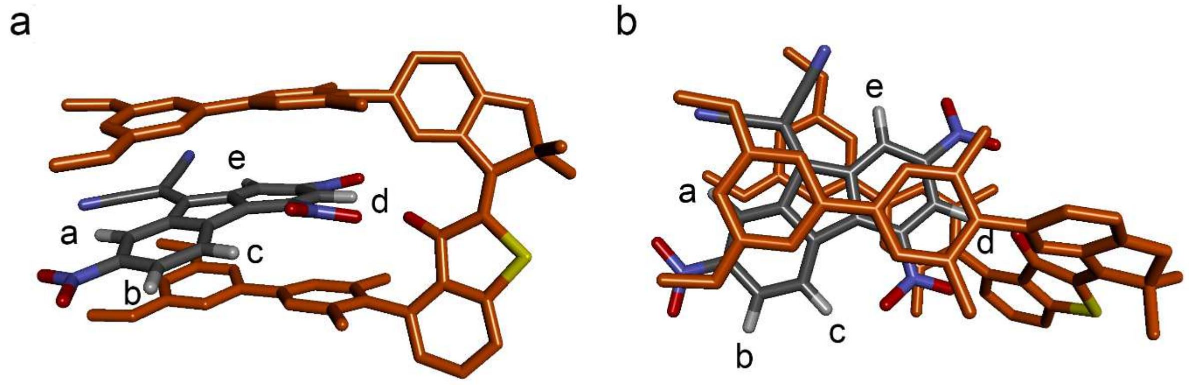

Supplementary Figure 15 | Molecular structure of the host-guest complex B (*E*-1·10). See also the corresponding data of complex **B** (second lowest DFT energy) in Supplementary Table 7. Calculations were performed at the B3LYP-GD3BJ/6-311G(d,p) level of theory. a) Side view. b) Top view. The guests proton signals a-d are indicated.

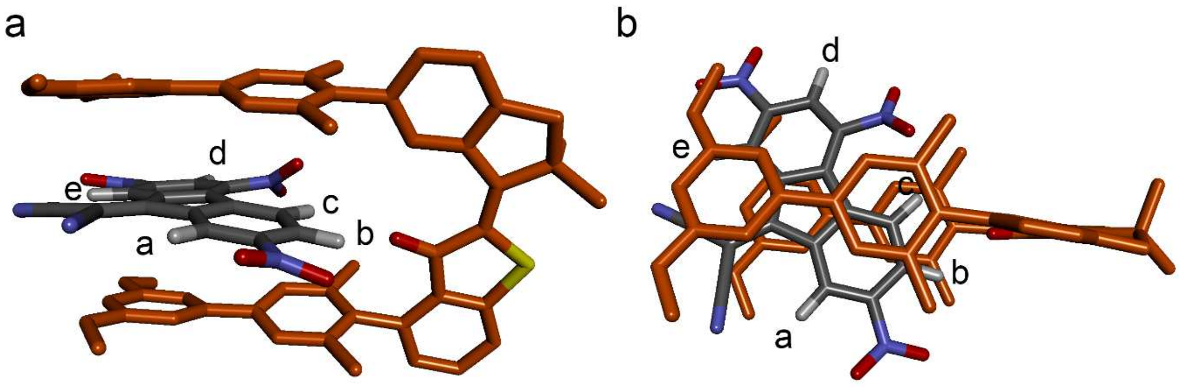

Supplementary Figure 16 | Molecular structure of the host-guest complex C (*E*-1·10). See also the corresponding data of complex **C** (third lowest DFT energy, all methoxy groups pointing backwards) in Supplementary Table 7. Calculations were performed at the B3LYP-GD3BJ/6-311G(d,p) level of theory. a) Side view. b) Top view. The guests proton signals a-d are indicated. Experimentally observed relative upfield-shift effects on the guest-proton signals are given.

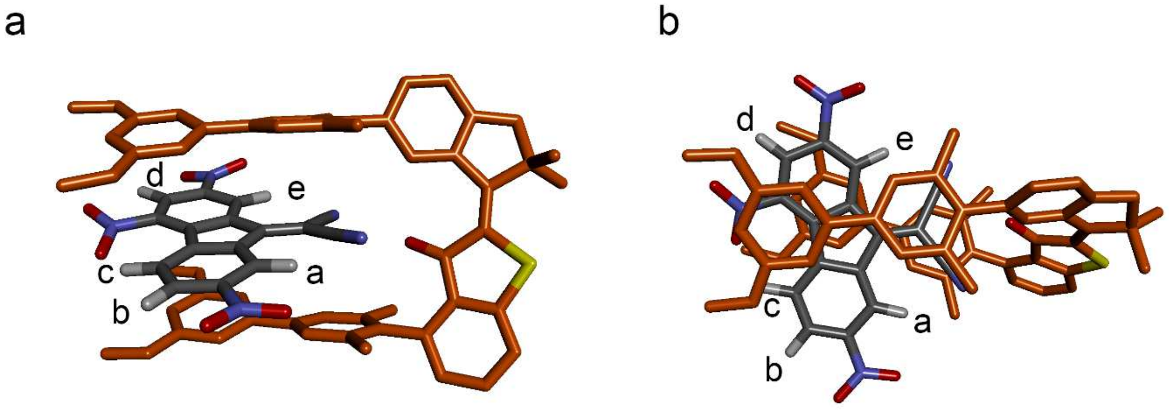

Supplementary Figure 17 | Molecular structure of the host-guest complex D (*E*-1·10). See also the corresponding data of complex **D** (fourth lowest DFT energy) in Supplementary Table 7. Calculations were performed at the B3LYP-GD3BJ/6-311G(d,p) level of theory. a) Side view. b) Top view. The guests proton signals a-d are indicated.

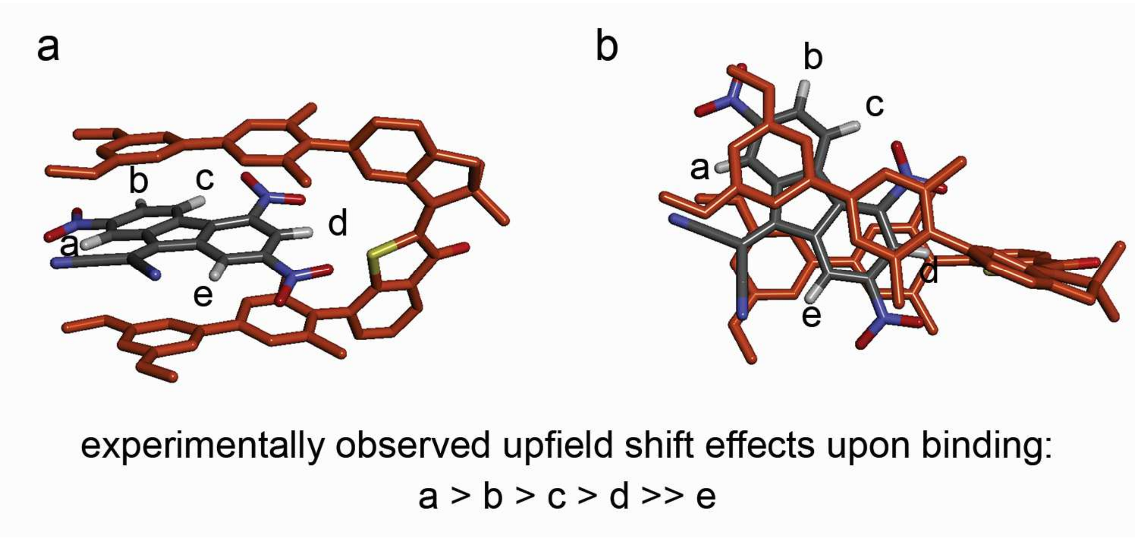

Supplementary Figure 18 | Molecular structure of the host-guest complex E (*Z*-2·10). See also the corresponding data of complex **E** (lowest DFT energy) in Supplementary Table 11. Calculations were performed at the B3LYP-GD3BJ/6-311G(d,p) level of theory. a) Side view. b) Top view. The guests proton signals a-d are indicated. Experimentally observed relative upfield-shift effects on the guest-proton signals are given.

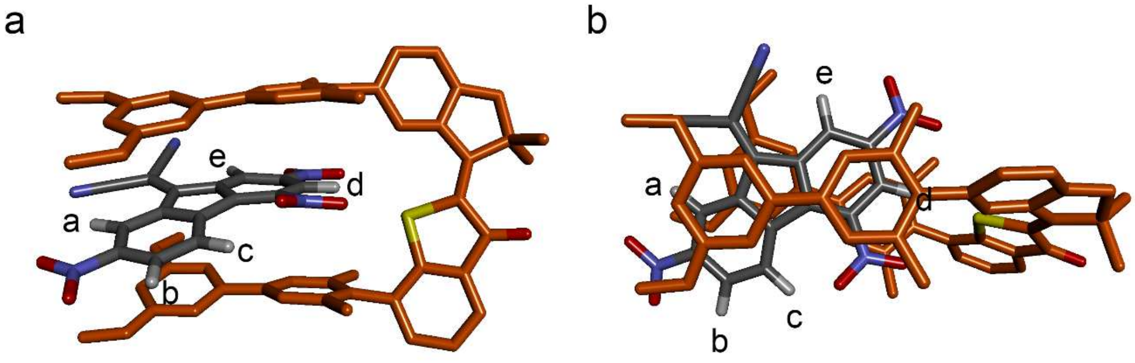

Supplementary Figure 19 | Molecular structure of the host-guest complex F (*Z*-2·10). See also the corresponding data of complex **F** (second lowest DFT energy) in Supplementary Table 11. Calculations were performed at the B3LYP-GD3BJ/6-311G(d,p) level of theory. a) Side view. b) Top view. The guests proton signals a-d are indicated.

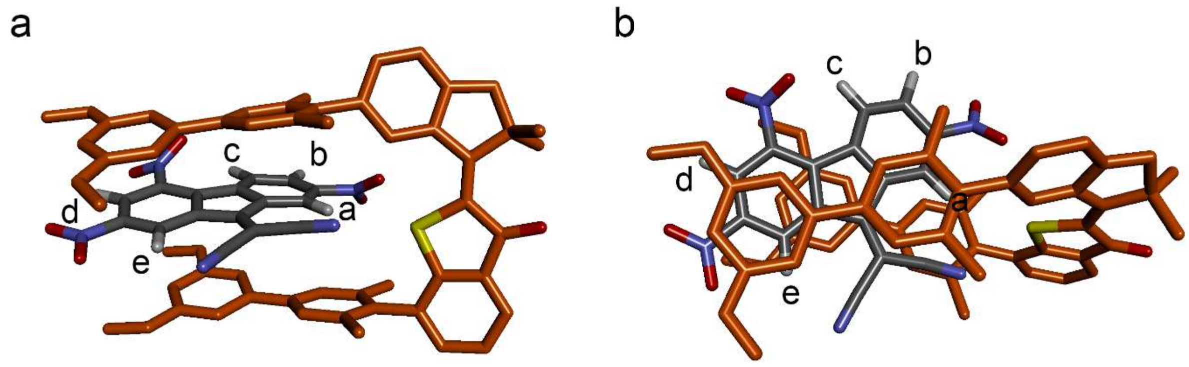

Supplementary Figure 20 | Molecular structure of the host-guest complex G (*Z*-2·10). See also the corresponding data of complex **G** (third lowest DFT energy) in Supplementary Table 11. Calculations were performed at the B3LYP-GD3BJ/6-311G(d,p) level of theory. a) Side view. b) Top view. The guests proton signals a-d are indicated.

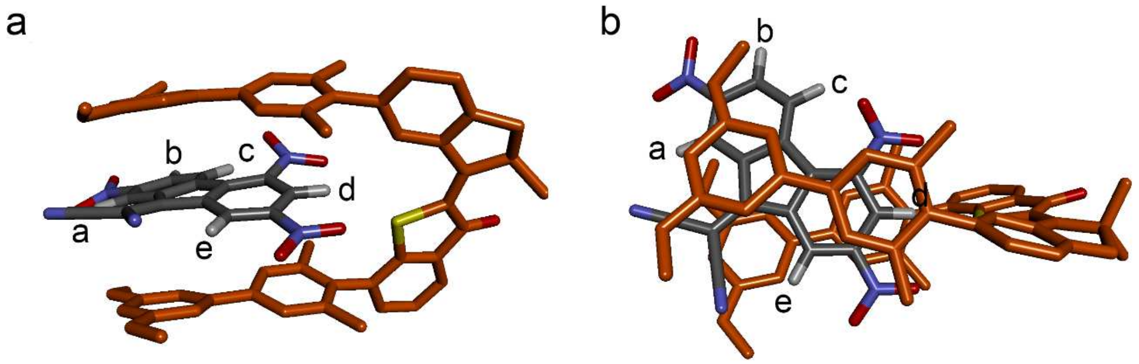

Supplementary Figure 21 | Molecular structure of the host-guest complex H (*Z*-2·10). See also the corresponding data of complex **H** (fourth lowest DFT energy, all methoxy groups pointing backwards) in Supplementary Table 11. Calculations were performed at the B3LYP-GD3BJ/6-311G(d,p) level of theory. a) Side view. b) Top view. The guests proton signals a-d are indicated.

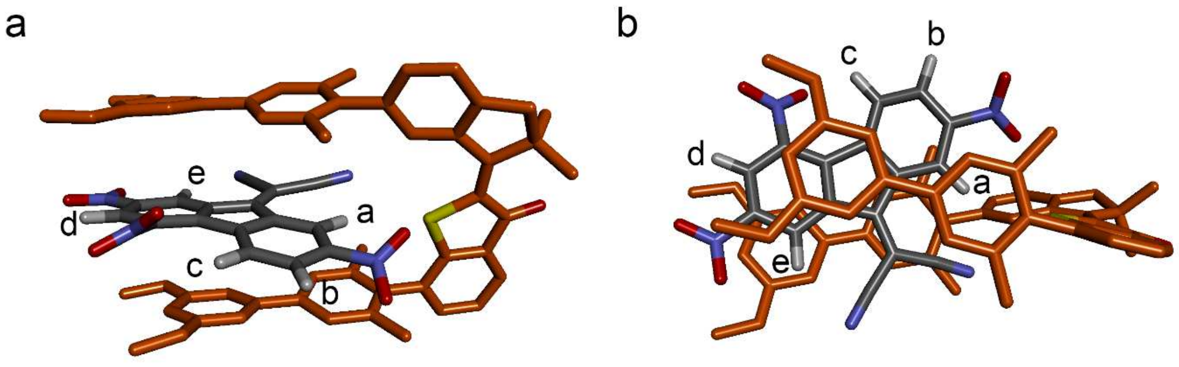

Supplementary Figure 22 | Molecular structure of the host-guest complex I (*Z*-2·10). See also the corresponding data of complex **I** (fifth lowest DFT energy) in Supplementary Table 11. Calculations were performed at the B3LYP-GD3BJ/6-311G(d,p) level of theory. a) Side view. b) Top view. The guests proton signals a-d are indicated.

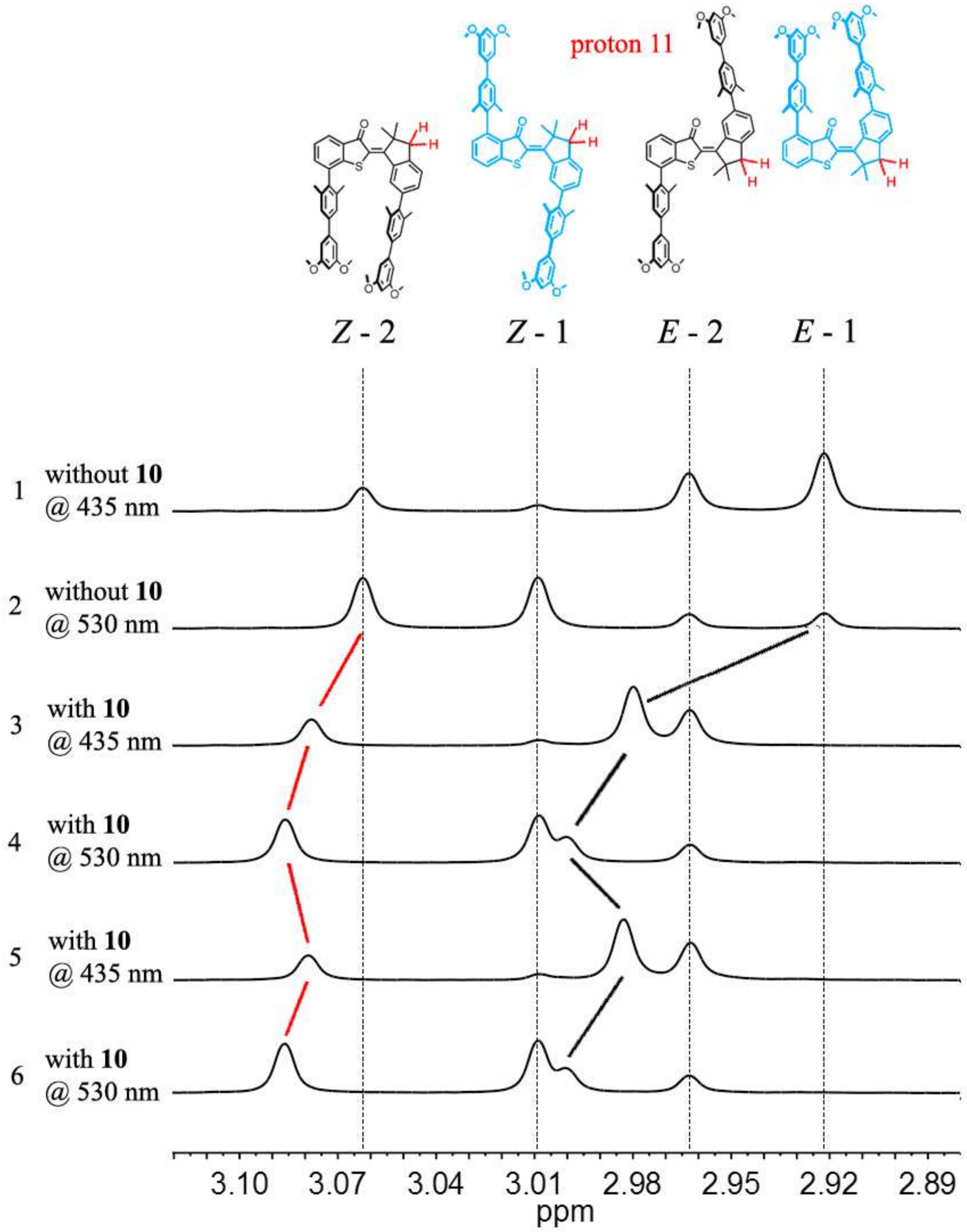

Supplementary Figure 23 | Simultaneous complementary photoswitching of 1 and 2 and dynamic guest relocation. Selected region of the ^{1}H NMR (400 MHz, 293 K, $CDCl_3$) spectra acquired during the process. The isolated signals of proton 11 (indicated in the structures on top) are shown. The different molecular species are assigned. 1: Pss of the 1:1 mixture of **1** and **2** at 435 nm. 2: Pss of the 1:1 mixture of **1** and **2** at 530 nm. 3: Pss of the 1:1 mixture of **1** and **2** at 435 nm in the presence of 0.6 equiv. of **10**. 4: Pss of the 1:1 mixture of **1** and **2** at 530 nm in the presence of 0.6 equiv. of **10**. 5 and 6: Repetition of the guest relocation experiments at 435 nm and 530 nm.

Supplementary Tables

Tweezers	% *E* Isomer (435 nm)	% *Z* Isomer (530 nm)
**1**	86 (15min)	80 (120 min)
**2**	63 (60 min)	84 (135 min)

Supplementary Table 1 | Individual isomer yields obtained in the pss. Isomer yields are reported for *Z*/*E* and *E*/*Z* photoisomerization at different wavelengths of irradiation for tweezers **1** and **2**. 5 mM solutions of the respective tweezers were irradiated in $CDCl_3$ at 25 °C. Duration times of the irradiation are given in brackets.

Tweezers	% *E* isomer (435 nm)	% *Z* isomer (530 nm)
**1**	88	82
**2**	64	85

Supplementary Table 2 | Isomer yields obtained in the pss of a 1:1 mixture of 1 and 2. Experiments were conducted in $CDCl_3$ solution (conc. = 2.2 mM for each tweezers) after 60 min of irradiation.

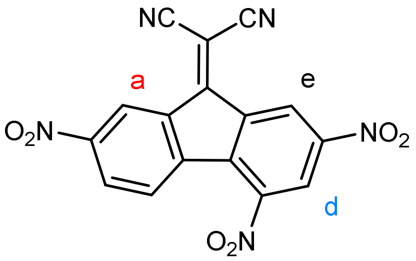

[**10**] in mol L^{-1}	[*E*-**1**] in mol L^{-1}	δH$_a$ in ppm	δH$_d$ in ppm	δH$_e$ in ppm
2.64E-04	0.00E+00	9.4879	8.9626	9.6735
2.64E-04	3.58E-04	9.1253	8.90455	9.1416
2.64E-04	6.53E-04	9.0418	8.8921	9.0203
2.64E-04	9.00E-04	9.0155	8.88765	8.9810
2.64E-04	1.11E-03	9.0003	8.88605	8.9602
2.64E-04	2.59E-03	8.9778	8.88295	8.9268
2.64E-04	2.80E-03	8.9760	8.8824	8.9231
2.64E-04	3.05E-03	8.9731	8.8827	8.9196
2.64E-04	3.34E-03	8.97155	8.88245	8.9160
2.64E-04	3.70E-03	8.97015	8.8822	8.9149

Supplementary Table 3 | ^{1}H chemical shift changes of 10 during titration with *E*-1. Changes of indicative proton signals of **10** were observed by ^{1}H NMR spectroscopy (400 MHz, 253 K, $CDCl_3$) during titration with *E*-**1**. The concentration of **10** was kept constant, the concentration of *E*-**1** is given for each titration step.

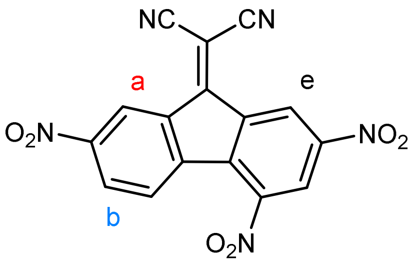

[**10**] in mol L^{-1}	[**2**] in mol L^{-1}	δH_a in ppm	δH_b in ppm	δH_e in ppm
2.52E-04	0.00E+00	9.48865	8.56515	9.64395
2.52E-04	2.97E-04	9.3399	8.4611	9.4218
2.52E-04	5.34E-04	9.2585	8.4044	9.2863
2.52E-04	7.28E-04	9.2177	8.3759	9.2177
2.52E-04	8.90E-04	9.1908	8.3561	9.1711
2.52E-04	1.03E-03	9.1697	8.3413	9.1342
2.52E-04	1.64E-03	9.1332	8.3179	9.0708
2.52E-04	1.78E-03	9.1283	8.31425	9.0679
2.52E-04	1.94E-03	9.1231	8.30945	9.0559
2.52E-04	2.13E-03	9.1155	8.30385	9.0419
2.52E-04	2.37E-03	9.1068	8.2976	9.0262
2.52E-04	2.67E-03	9.0985	8.2918	9.0127

Supplementary Table 4 | ^{1}H chemical shift changes of 10 during titration with *Z*-2. Changes of indicative proton signals of **10** were observed by ^{1}H NMR spectroscopy (400 MHz, 253 K, $CDCl_3$) during titration with *Z*-**2**. The concentration of **10** was kept constant, the concentration of *Z*-**2** is given for each titration step.

proton number	experimental chemical shift δ in ppm	theoretical (B3LYP) chemical shift δ in ppm	theoretical (B3LYP-GD3BJ) chemical shift δ in ppm
2	--	7.02	7.10
3	--	7.68	7.69
4	--	7.49	7.50
11/11‘	2.97	3.00	2.93
14	8.12	8.75	7.99
16	--	7.36	7.16
17	--	7.43	7.37
20/22	7.17	7.34	6.92
25/29	6.70	6.73	6.63
27	6.45	6.37	6.55
32/34	7.20	7.36	7.12
37/41	6.75	6.76	6.66
39	6.43	6.39	6.00
42/43	1.93	1.91	1.90
44/45	3.75	3.88	3.58
46/47	2.02	2.12	1.87
48/49	3.84	3.82	3.95
50/51	1.51	1.54	1.46
MSD	--	-0.09	0.09
MAD	--	0.11	0.12
STD	--	0.18	0.14

Supplementary Table 5 | Comparison of experimental and calculated ^{1}H shifts of *E*-1. Calculations were performed at the B3LYP level of theory with and without GD3BJ. For the numbering of the protons see Supplementary Table 10.

proton number	experimental chemical shift δ in ppm	theoretical (B3LYP) chemical shift δ in ppm	theoretical (B3LYP-GD3BJ) chemical shift δ in ppm
1	--	8.26	8.17
2	--	7.50	7.54
3	--	7.45	7.61
11/11‘	3.11	3.15	3.16
14	7.72	8.07	8.10
16	7.13	7.22	7.39
17	--	7.59	7.45
20/22	7.24	7.32	7.06
27	6.45	6.37	6.56
32/34	7.30	7.47	7.49
25/29	6.67	6.71	6.54
37/41	6.73	6.71	6.82
39	6.43	6.39	6.65
42/43	2.07	2.05	2.03
44/45	3.76	3.79	3.90
46/47	2.04	2.00	2.02
48/49	3.82	3.79	3.93
50/51	--	1.80	1.76
MSD	--	-0.04	-0.09
MAD	--	0.08	0.15
STD	--	0.18	0.18

Supplementary Table 6 | Comparison of experimental and calculated ^{1}H shifts of *Z*-2. Calculations were performed at on the B3LYP level of theory with and without GD3BJ. For the numbering of the protons see Supplementary Table 14.

.

tweezers *E*-**1** proton number (see Supplementary Table 10)	experimental chemical shift δ in ppm	theoretical chemical shift δ of complex A (Supplementary Figure 14) in ppm	theoretical chemical shift δ of complex B (Supplementary Figure 15) in ppm	theoretical chemical shift δ of complex C (Supplementary Figure 16) in ppm	theoretical chemical shift δ of complex D (Supplementary Figure 17) in ppm	averaged theoretical chemical shift δ in ppm	DFT-energy Boltzmann averaged theoretical chemical shift δ in ppm
2	--	6.62	6.65	6.69	7.02	6.74	6.62
3	--	7.67	7.52	7.70	7.64	7.63	7.67
4	--	7.55	7.54	7.59	7.58	7.57	7.55
11/11‘	3.02	3.20	3.15	3.20	3.05	3.21	3.20
14	8.47	10.61	9.27	10.60	10.07	10.14	10.59
16	--	7.25	7.25	7.21	7.28	7.25	7.25
17	--	7.57	7.54	7.56	7.59	7.57	7.57
20/22	6.97	6.59	6.88	6.65	6.73	6.71	6.59
25/29	6.48	5.87	6.60	5.82	5.50	5.94	5.88
27	6.42	6.59	5.69	7.13	6.05	6.36	6.57
32/34	7.04	6.66	6.75	6.80	7.11	6.83	6.66
37/41	6.52	5.77	6.23	5.44	6.20	5.91	5.78
39	6.32	6.17	6.40	6.82	4.99	6.10	6.18
42/43	1.91	1.93	1.90	2.03	2.27	2.03	1.93
44/45	3.72	3.88	4.05	3.55	3.45	3.73	3.88
46/47	1.94	1.75	1.89	1.76	2.29	1.92	1.75
48/49	3.83	4.11	3.62	3.52	3.89	3.78	4.10
50/51	1.58	1.78	1.64	1.77	1.66	1.71	1.77
MSD	--	0.05	-0.01	0.06	-0.08	0.01	0.05
MAD	--	0.43	0.25	0.52	0.47	0.31	0.42
STD	--	0.71	0.34	0.76	0.59	0.55	0.70

Supplementary Table 7 | Experimental and calculated ^{1}H NMR shifts of *E*-1 in *E*-1·10. Comparison of experimentally obtained values with calculated ^{1}H shifts for *E*-**1** in the host-guest complexes *E*-**1**·**10** with different geometries **A** to **D**. The theoretically obtained chemical shifts were averaged as well as DFT-energy-based Boltzmann-averaged. The corresponding overall agreement between theory and experiment is given as mean signed deviation (MSD = $\frac{1}{N}\sum_{A}^{N}\delta_{theor(A)} - \delta_{exp(A)}$), mean absolute deviation (MAD = $\frac{1}{N}\sum_{A}^{N}|\delta_{theor(A)} - \delta_{exp(A)}|$), and standard deviation (STD = $\sqrt{\frac{1}{N-1}\sum_{A}^{N}(\mathrm{MSD} - (\delta_{theor(A)} - \delta_{exp(A)}))^2}$).

	experimental values				theoretical values			
proton #	proton shift unbound [ppm]	proton shift bound [ppm]	$\Delta\delta$ [ppm]	tendency	proton shift unbound [ppm]	averaged proton shift bound [ppm]	$\Delta\delta$ [ppm]	tendency
a	9.49	8.99 (8.97)	-0.5	U	9.9	9.49	-0.41	U
b	8.57	8.24 (8.23)	-0.33	U	8.78	8.92	0.14	D
c	8.23	7.57	-0.66	U	8.66	8.3	-0.36	U
d	8.96	8.88 (8.88)	-0.08	U	9.16	9.43	0.27	D
e	9.67	8.96 (8.91)	-0.71	U	10.1	9.52	-0.58	U

Supplementary Table 8 | Experimental and calculated ^{1}H shifts of 10 in *E*-1·10. Comparison of experimental ^{1}H shifts of **10** observed before and after addition of *E*-**1** with calculated shifts for **10** and the host guest complex *E*-**1**·**10**. Experimental chemical shifts were obtained after addition of 4.2 equiv. *E*-**1** to be able to trace all signals (later in the titration some signals are obscured by signal overlap). Note that the experimental shifts observed at the saturation point are given in brackets. For the numbering of the protons see the titration part of **10** and Supplementary Figures 14-17. To decide the tendency of the shifts, a threshold for $\Delta\delta$ of 0.05 ppm was applied. D = downfield shift; U = upfield shift; N = neutral.

guest **10** proton number	exper. chemical shift δ in ppm	theor. chemical shift δ of complex A (Supplementary Figure 14) in ppm	theor. chemical shift δ of complex B (Supplementary Figure 15) in ppm	theor. chemical shift δ of complex C (Supplementary Figure 16) in ppm	theor. chemical shift δ of complex D (Supplementary Figure 17) in ppm	averaged theor. chemical shift δ in ppm	DFT-energy Boltzm. averaged theoretical chemical shift δ in ppm
a	8.99	9.49	9.34	9.40	9.75	9.49	9.48
b	8.24	8.98	8.77	9.07	8.84	8.92	8.97
c	7.57	7.81	8.76	7.44	9.18	8.30	7.83
d	8.88	9.31	10.14	9.10	9.17	9.43	9.33
e	8.96	9.10	9.21	9.96	9.79	9.52	9.11
MSD	--	0.41	0.71	0.47	0.82	0.60	0.42
MAD	--	0.41	0.71	0.52	0.82	0.60	0.42
STD	--	0.23	0.47	0.46	0.49	0.09	0.23

Supplementary Table 9 | Experimental and calculated ^{1}H NMR shifts of 10 in *E*-1·10. Comparison of experimentally obtained values with calculated ^{1}H shifts for the guest **10** in host-guest complexes *E*-**1**·**10** with different geometries **A** to **D**. The theoretically obtained chemical shifts were averaged as well as DFT-energy-based Boltzmann-averaged. The corresponding overall agreement between theory and experiment is given as mean signed deviation (MSD = $\frac{1}{N}\sum_A^N \delta_{theor(A)} - \delta_{exp(A)}$), mean absolute deviation (MAD = $\frac{1}{N}\sum_A^N |\delta_{theor(A)} - \delta_{exp(A)}|$), and standard deviation (STD = $\sqrt{\frac{1}{N-1}\sum_A^N (MSD - (\delta_{theor(A)} - \delta_{exp(A)}))^2}$).

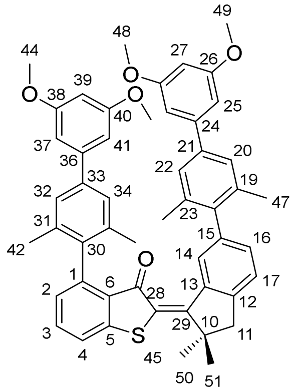

	experimental values				theoretical values			
proton #	proton shift unbound [ppm]	proton shift bound [ppm]	$\Delta\delta$ [ppm]	tendency	proton shift unbound [ppm]	averaged proton shift bound [ppm]	$\Delta\delta$ [ppm]	tendency
11	2.97	3.02	0.05	D	3	3.2	0.2	D
14	8.12	8.47	0.35	D	8.75	10.65	1.9	D
27	6.45	6.42	-0.03	N	6.38	6.65	0.27	D
39	6.43	6.32	-0.11	U	6.39	6.17	-0.22	U
20,22	7.17	6.97	-0.2	U	7.36	6.62	-0.74	U
44,45	3.75	3.72	-0.03	N	3.89	3.89	0	N
25,29	6.7	6.48	-0.22	U	6.73	5.89	-0.84	U
32,34	7.2	7.04	-0.16	U	7.39	6.68	-0.71	U
37,41	6.75	6.52	-0.23	U	6.76	5.79	-0.97	U
42,43	1.93	1.91	-0.02	N	1.92	1.94	0.02	N
46,47	2.02	1.94	-0.08	U	2.12	1.77	-0.35	U
48,49	3.84	3.83	-0.01	N	3.83	4.12	0.29	D
50, 51	1.51	1.58	0.07	D	1.54	1.78	0.24	D

Supplementary Table 10 | Experimental and calculated ^{1}H shifts of *E*-1 in *E*-1·10. Comparison of experimental ^{1}H shifts of *E*-**1** observed before and after addition of 2.7 equiv. of **10** with calculated shifts for *E*-**1** and the host-guest complex *E*-**1**·**10**. For the numbering of the protons see the structural depiction. To decide the trend of the shift, a threshold for $\Delta\delta$ of 0.05 ppm was applied. D = downfield shift; U = upfield shift; N = neutral.

.

tweezers *Z*-**2** proton number (see Supplementary Table 14)	experimental chemical shift *δ* in ppm	theoretical chemical shift *δ* of complex E (Supplementary Figure 18) in ppm	theoretical chemical shift *δ* of complex F (Supplementary Figure 19) in ppm	theoretical chemical shift *δ* of complex G (Supplementary Figure 20) in ppm	theoretical chemical shift *δ* of complex H (Supplementary Figure 21) in ppm	theoretical chemical shift *δ* of complex I (Supplementary Figure 22) in ppm	averaged theoretical chemical shift *δ* in ppm	DFT-energy Boltzmann averaged theoretical chemical shift *δ* in ppm
1	--	8.30	8.34	8.30	8.27	8.30	8.30	8.31
2	--	7.40	7.42	7.45	7.40	7.44	7.42	7.40
3	--	7.05	7.14	7.26	7.04	7.26	7.15	7.07
11/11‘	3.13	3.25	3.27	3.18	3.28	3.18	3.23	3.26
14	7.78	8.98	8.86	8.85	8.75	8.84	8.86	8.95
16	7.11	7.27	7.31	6.98	7.37	7.02	7.19	7.28
17	--	7.61	7.59	7.45	7.64	7.44	7.54	7.60
20/22	7.13	6.85	6.83	7.19	6.91	7.16	6.99	6.85
27	6.41	5.66	5.53	6.02	6.42	5.90	5.91	5.63
32/34	7.21	6.62	6.90	7.25	6.42	7.25	6.89	6.68
25/29	6.55	6.61	6.63	6.55	6.44	6.72	6.59	6.61
37/41	6.60	5.98	5.65	6.27	5.97	6.25	6.02	5.91
39	6.36	6.78	6.25	5.04	6.99	5.01	6.02	6.67
42/43	2.05	1.91	2.06	2.15	1.91	2.17	2.04	1.94
44/45	3.73	4.09	3.72	3.40	3.59	3.41	3.64	4.01
46/47	2.01	1.85	1.84	1.71	1.93	1.70	1.81	1.85
48/49	3.80	3.59	3.60	3.97	3.72	3.82	3.74	3.59
50/51	--	1.92	1.93	1.90	1.90	1.90	1.91	1.92
MSD	--	0.03	0.04	0.05	-0.01	0.04	0.07	-0.05
MAD	--	0.39	0.49	0.48	0.32	0.49	0.27	0.37
STD	--	0.52	0.52	0.54	0.46	0.55	0.41	0.51

Supplementary Table 11 | Experimental and calculated ^{1}H NMR shifts of *Z*-2 in *Z*-2·10. Comparison of experimentally obtained values with calculated ^{1}H shifts for *Z*-**2** in host-guest complexes *Z*-**2**·**10** with different geometries **E** to **I**. The theoretically obtained chemical shifts were averaged as well as DFT-energy-based Boltzmann- averaged. The corresponding overall agreement between theory and experiment is given as mean signed deviation ($\mathrm{MSD} = \frac{1}{N}\sum_A^N \delta_{theor(A)} - \delta_{exp(A)}$), mean absolute deviation ($\mathrm{MAD} = \frac{1}{N}\sum_A^N |\delta_{theor(A)} - \delta_{exp(A)}|$), and standard deviation ($\mathrm{STD} = \sqrt{\frac{1}{N-1}\sum_A^N (\mathrm{MSD} - (\delta_{theor(A)} - \delta_{exp(A)}))^2}$).

	experimental values				theoretical values			
proton #	proton shift unbound [ppm]	proton shift bound [ppm]	Δ*δ* [ppm]	tendency	proton shift unbound [ppm]	averaged proton shift bound [ppm]	Δ*δ* [ppm]	tendency
a	9.49	9.01	-0.57	U	9.9	8.83	-1.07	U
b	8.57	8.30	-0.27	U	8.78	8.41	-0.37	U
c	8.23	7.58	-0.65	U	8.66	8.51	-0.15	U
d	8.96	8.67	-0.29	U	9.16	8.83	-0.33	U
e	9.67	9.10	-0.57	U	10.1	9.16	-0.94	U

Supplementary Table 12 | Experimental and calculated ^{1}H shifts of 10 in *Z*-2·10. Comparison of experimental ^{1}H shifts of **10** observed before and after addition of 10.6 equiv. *Z*-**2** with calculated shifts for **10** and the host guest complex *E*-**1**·**10**. For the numbering of the protons see the titration part of **10** and Supplementary Figures 18-22. To decide the tendency of the shifts, a threshold for Δδ of 0.05 ppm was applied. D = downfield shift; U = upfield shift; N = neutral.

guest **10** proton number	exper. chemical shift δ in ppm	theor. chemical shift *δ* of complex E (Supplementary Figure18) in ppm	theor. chemical shift *δ* of complex F (Supplementary Figure 19) in ppm	theor. chemical shift *δ* of complex G (Supplementary Figure 20) in ppm	theor. chemical shift *δ* of complex H (Supplementary Figure 21) in ppm	theor. chemical shift *δ* of complex I (Supplementary Figure 22) in ppm	averaged theor. chemical shift *δ* in ppm	DFT-energy Boltzm. averaged theoretical chemical shift *δ* in ppm
a	9.01	9.34	9.54	7.71	9.78	7.77	8.83	9.38
b	8.30	8.76	8.75	7.95	8.65	7.96	8.41	8.75
c	7.58	8.81	8.90	8.13	8.56	8.17	8.51	8.83
d	8.67	8.87	8.86	8.89	8.58	8.93	8.83	8.87
e	9.10	9.28	9.44	9.05	9.08	8.95	9.16	9.31
MSD	--	0.48	0.56	-0.19	0.40	-0.18	0.22	0.50
MAD	--	0.48	0.56	0.49	0.44	0.52	0.29	0.50
STD	--	0.44	0.48	0.70	0.47	0.69	0.42	0.44

Supplementary Table 13 | Experimental and calculated ^{1}H NMR shifts of 10 in *Z*-2·10. Comparison of experimentally obtained values with calculated ^{1}H shifts for the guest **10** in host-guest complexes *Z*-**2**·**10** with different geometries **E** to **I**. The theoretically obtained chemical shifts were averaged as well as DFT-energy-based Boltzmann-averaged. The corresponding overall agreement between theory and experiment is given as mean signed deviation ($\mathrm{MSD} = \frac{1}{N}\sum_A^N \delta_{theor(A)} - \delta_{exp(A)}$), mean absolute deviation ($\mathrm{MAD} = \frac{1}{N}\sum_A^N |\delta_{theor(A)} - \delta_{exp(A)}|$), and standard deviation ($\mathrm{STD} = \sqrt{\frac{1}{N-1}\sum_A^N(\mathrm{MSD} - (\delta_{theor(A)} - \delta_{exp(A)}))^2}$).

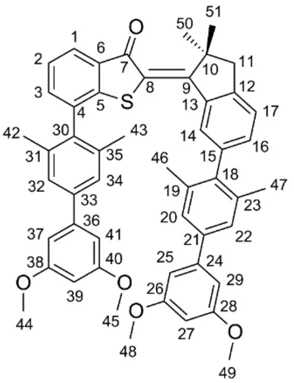

	experimental values				theoretical values			
proton #	proton shift unbound [ppm]	proton shift bound [ppm]	Δ*δ* [ppm]	tendency	proton shift unbound [ppm]	averaged proton shift bound [ppm]	Δ*δ* [ppm]	tendency
11	3.11	3.13	0.02	N	3.15	3.23	0.08	D
14	7.72	7.78	0.06	D	8.07	8.86	0.79	D
16	7.13	7.11	-0.02	N	7.22	7.19	-0.03	N
27	6.45	6.41	-0.04	N	6.37	5.91	-0.46	U
39	6.43	6.36	-0.07	U	6.39	6.02	-0.37	U
20,22	7.24	7.13	-0.11	U	7.32	6.99	-0.33	U
25,29	6.67	6.55	-0.12	U	6.71	6.59	-0.12	U
32,34	7.3	7.21	-0.09	U	7.47	6.89	-0.58	U
37,41	6.73	6.6	-0.13	U	6.71	6.02	-0.69	U
42,43	2.07	2.05	-0.02	N	2.05	2.04	-0.01	N
44,45	3.76	3.73	-0.03	N	3.79	3.64	-0.15	U
46,47	2.04	2.01	-0.03	N	2	1.81	-0.19	U
48,49	3.82	3.8	-0.02	N	3.79	3.74	-0.05	N

Supplementary Table 14 | Experimental and calculated ^{1}H shifts of *Z*-2 in *Z*-2·10. Comparison of experimental ^{1}H shifts of *Z*-**2** observed before and after addition of 2.9 equiv. of **10** with calculated shifts for *Z*-**2** and the host-guest complex *Z*-**2**·**10**. For the numbering of the protons see the structural depiction. To decide the trend of the shift, a threshold for Δ*δ* of 0.05 ppm was applied. D = downfield shift; U = upfield shift; N = neutral.

	**5** *E* isomer
net formula	$C_{19}H_{14}Br_2OS$
M_r/g mol^{-1}	450.18
crystal size/mm	0.060 × 0.050 × 0.040
T/K	293.(2)
radiation	MoKα
diffractometer	'Bruker D8 Venture TXS'
crystal system	triclinic
space group	'P -1'
a/Å	9.3672(5)
b/Å	10.0095(4)
c/Å	10.6908(5)
α/°	110.6760(10)
β/°	104.7190(10)
γ/°	100.8020(10)
V/Å^3	863.46(7)
Z	2
calc. density/g cm^{-3}	1.732
μ/mm^{-1}	4.816
absorption correction	Multi-Scan
transmission factor range	0.6697–0.7454
refls. measured	14321
R_{int}	0.0285
mean σ(*I*)/*I*	0.0265
θ range	3.326–25.344
observed refls.	2572
x, y (weighting scheme)	0.0286, 0.6870
hydrogen refinement	constr
refls in refinement	3148
parameters	210
restraints	0
$R(F_{obs})$	0.0299
$R_w(F^2)$	0.0739
S	1.047
shift/error$_{max}$	0.001
max electron density/e Å^{-3}	0.640
min electron density/e Å^{-3}	–0.309

Supplementary Table 15 | Crystal structural data for *E*-5 (CCDC 1578150).

Supplementary Methods

General experimental

Reagents and solvents were obtained from *Acros*, *Aldrich*, *Fluka*, *Merck*, or *Sigma-Aldrich* in the qualities *puriss*., *p.a*., or *purum* and used as recieved. Technical solvents were distilled before column chromatography and extraction using a rotary evaporator (*vacuubrand* CVC 3000). Reactions were monitored on *Merck* Silica 60 F254 TLC plates. Detection was done by irradiation with UV light (254 nm or 366 nm).

Column chromatography was performed with silica gel 60 (*Merck*, particle size 0.063 – 0.200 mm) and distilled technical solvents.

High Performance Liquid Chromatography (HPLC) was performed on a *Shimadzu* HPLC system consisting of a *LC-20AP* solvent delivery module, a *CTO-20A* column oven, a *SPD-M20A* photodiode array Ultraviolet/vis detector and a *CBM-20A* system controller using a preparative *NUCLEODUR*® 100-5 column (particle size 5 µm) from *Macherey-Nagel* and HPLC grade solvents (*i*-propanol and *n*-heptane) from *Sigma-Aldrich* and *Alfa Aesar*.

^{1}H NMR and ^{13}C NMR spectra were measured on a *Varian Mercury 200 VX*, *Varian 300, Inova 400*, *Varian 600 NMR*, or *Bruker Avance III HD 800 MHz* spectrometer at various temperatures. Titrations were performed on a *Jeol ECX 400* at 253 K. Deuterated solvents were obtained from *Cambridge Isotope Laboratories* and used without further purification. Chemical shifts (δ) are given relative to tetramethylsilane as external standard. Residual solvent signals in the ^{1}H and ^{13}C NMR spectra were used as internal reference. For ^{1}H NMR: $CDCl_3$ = 7.26 ppm, CD_2Cl_2 = 5.32 ppm, acetone-d_6 = 2.05 ppm, DMSO-d_6 = 2.50 ppm. For ^{13}C NMR: $CDCl_3$ = 77.16 ppm, CD_2Cl_2 = 53.84 ppm, acetone-d_6 = 206.68, 29.92 ppm, DMSO-d_6 = 39.52 ppm. The resonance multiplicity is indicated as *s* (singlet), *d* (doublet), *t* (triplet), *q* (quartet) and *m* (multiplet) and *br* (broad signal). The chemical shifts are given in parts per million (ppm) on the delta scale (δ), and the coupling constant values (J) are given in hertz (Hz).

Electron Impact (EI) mass spectra were measured on a *Finnigan MAT95Q* or on a *Finnigan MAT90* mass spectrometer. **Electronspray ionisation (ESI) mass spectra** were measured on a *Thermo Finnigan LTQ-FT*. The most important signals are reported in *m*/*z* units with *M* as the molecular ion.

Elemental analyses were performed in the micro analytical laboratory of the Ludwig-Maximilians-Universität München department of chemistry on an *Elementar Vario EL* apparatus.

Infrared spectra were recorded on a *Perkin Elmer Spectrum BX-FT-IR* instrument equipped with a Smith *DuraSamplIR II* ATR-device. Transmittance values are qualitatively described by wavenumber (cm^{-1}) as strong (*s*), medium (*m*) and weak (*w*).

Isomerization experiments: LED-diodes used for the isomerization experiments were purchased from *Roithner Lasertechnik GmbH* (435 nm: H2A1-H435, 530 nm: H2A3-H530).

Ultraviolet/vis absorption spectra were measured on a *Varian Cary 5000* spectrophotometer. The spectra were recorded in a quartz cuvette (1 cm). Solvents for spectroscopy were obtained from *VWR* and *Merck*. Absorption wavelength (λ) are reported in nm and the extinction coefficients (ε) $L\cdot mol^{-1}\cdot cm^{-1}$ in brackets. Shoulders are declared as *sh*.

Melting points (mp.) were measured on a *Büchi B-540 melting point* apparatus in open capillaries.

3,5-Dimethoxyphenylboronic acid (**7**), bromoacetic acid, 9-fluorenon, and 3-bromothiophenol were purchased as reagent grade from *Sigma-Aldrich* and 2,5-dibromoxylene (**6**) from *Fluorochem* and used as received.

2,4,7-trinitro-*9H*-fluoren-9-one was prepared according to published procedures[3].

Synthesis of 4-(3',5'-Dimethoxy-3,5-dimethyl-[1,1'-biphenyl]-4-yl)-2-(6-(3',5'-dimethoxy-3,5-dimethyl-[1,1'-biphenyl]-4-yl)-2,2-dimethyl-2,3-dihydro-1*H*-inden-1-ylidene)benzo[*b*]thiophen-3(2*H*)-one (1)

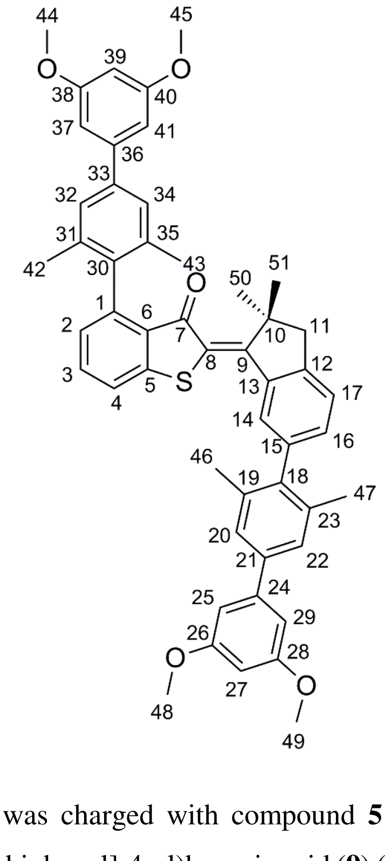

Z isomer

A flame-dried Schlenk tube was charged with compound **5** (1.00 eq, 100 mg, 0.22 mmol), (3',5'-dimethoxy-3,5-dimethyl-[1,1'-biphenyl]-4-yl)boronic acid (**9**) (4.00 eq, 254 mg, 0.88 mmol), and K_3PO_4

(6.00 eq, 282 mg, 1.34 mmol). The tube was evacuated and backfilled with argon (3 cycles). Toluene (600 µL), ethanol (600 µL), and H_2O (200 µL) were added. The solution was degassed by 3 freeze-pump-thaw cycles, before sSPhos Pd G2 (14.6 mg, 17.78 nmol, 8 mol%) was added. The reaction mixture was stirred for 18 h at 80 °C and subsequently allowed to cool to 23 °C. Then a saturated ammonium chloride solution (20 mL) was added. The aqueous phase was extracted with ethyl acetate (3 x 50 mL), the organic phases were separated and dried over Na_2SO_4. The solvent was removed *in vacuo*. The crude product was purified by column chromatography (SiO_2, *i*Hex/EtOAc 9:1) followed by NP-HPLC (SiO_2, *n*-Heptane/*i*-Propanol 99:1, t_R = *Z* isomer: 10.6 min., *E* isomer: 11.5 min., oven temperature 40 °C) to afford *Z*-**1** and *E*-**1** (combined isomers 110 mg, 64%) as yellow solid.

mp: 122 °C; TLC (SiO_2, *i*Hex/EtOAc 8:2 v/v): R_f= 0.61;

Z isomer:

^{1}H NMR (800 MHz, $CDCl_3$) δ 7.88 (d, *J*(H,H)=1.2 Hz, 1H; H-C(14)), 7.51 (dd, *J*(H,H)=7.9, 7.2 Hz, 1H; C-H(3)), 7.38 (dd, *J*(H,H)=7.9, 1.0 Hz, 1H; H-C(4)), 7.36 (s, 2H; H-C(20), H-C(22)), 7.34 (d, *J*(H,H)=8.0 Hz, 1H; H-C(17)), 7.33 (s, 2H; H-C(32), H-C(34)), 7.19 (dd, *J*(H,H)=7.5, 1.4 Hz, 1H; H-C(16)), 6.91 (dd, *J*(H,H)=7.2, 1.0 Hz, 1H; H-C(2)), 6.83 (d, *J*(H,H)=2.3 Hz, 2H; H-C(37), H-C(41)), 6.79 (d, *J*(H,H)=2.2 Hz, 2H; H-C(29), H-C(45)), 6.48 (t, *J*(H,H)=2.2 Hz, 1H; H-C(27)), 6.45 (t, *J*(H,H)=2.2 Hz, 1H; H-C(39)), 3.87 (2 s, 12H; H-C(25), H-C(44), H-C(48), H-C(49)), 3.06 (s, 2H; H-C(11)), 2.17 (s, 6H; H_3-C(46), H_3-C(47)), 2.00 (s, 6H; H_3-C(42), H_3-C(43)), 1.53 (s, 6H; H_3-C(50), H_3-C(51)); ^{13}C NMR (201 MHz, $CDCl_3$) δ 187.5 (C(7)), 162.2 (C(9)), 161.3 (C(26,28)), 161.2 (C(38,40)), 148.1 (C(12)), 145.7 (C(4 or 6)), 143.8 (C(36)), 143.6 (C(24)), 142.0 (C(4 or 6)), 141.1 (C(18)), 140.7 (C(8)), 140.4 (C(21)), 139.9 (C(13)), 139.7 (C(33)), 138.6 (C(30)), 137.0 (C(19), C(23)), 135.8 (C(31), C(35)), 133.8 (C(3)), 132.1 (C(16)), 129.1 (C(6)), 128.6 (C(14)), 127.6 (C(2)), 126.5 (C(20), C(22)), 126.2 (C(32), C(34)), 125.5 (C(15)), 122.5 (C(4)), 105.6 (C(37), C(41)), 105.6 (C(29), C(45)), 99.6 (C(27), C(39)), 55.7 (C(25), C(44), C(48) C(49)), 51.2 (C(18)), 48.9 (C(26)), 25.9 (C(50), C(51)), 21.4 (C(46), C(47)), 20.9 (C(42), C(43)); IR (ṽ): 2949w, 2836w, 2360w, 2337w, 1666m, 1593vs, 1563s, 1527m, 1456s, 1427m, 1387m, 1353m, 1297w, 1266m, 1229m, 1202s, 1173m, 1151vs, 1063s, 1054s, 1013w, 975w, 940w, 927w, 900w, 876w, 827m, 804m, 782m, 748w, 738w, 729w, 709w, 694w, 683w, 668w cm^{-1}; HREI-MS (*m/z*): $[M]^+$ calcd. for $C_{51}H_{48}O_5S$, 772.3222, found, 772.3220; analysis (calcd., found for $C_{51}H_{48}O_5S$): C (79.24, 78.9), H (6.26, 6.68), S (4.15, 4.34); UV-Vis ($CHCl_3$): λ_{max} (ε) = 268 (53,300 l mol^{-1} cm^{-1}), 329 (15,200 l mol^{-1} cm^{-1}), 347*sh* (11,800 l mol^{-1} cm^{-1}), 452 nm (15,400 l mol^{-1} cm^{-1}).

E isomer:

^{1}H NMR (600 MHz, $CDCl_3$) δ 8.12 (d, *J*(H,H)=1.4 Hz, 1H; H-C(14)), 7.54 (t, *J*(H,H)=7.6 Hz, 1H; HC(3)), 7.44 (dd, *J*(H,H)=7.9, 0.9 Hz, 1H; H-C(4)), 7.22 (dd, *J*(H,H)=7.7, 0.8 Hz, 1H; H-C(17)), 7.20 (d, *J*(H,H)=0.7 Hz, 2H; H-C(32), H-C(34)), 7.17 (d, *J*(H,H)=0.6 Hz, 2H; H-C(20), H-C(22)), 7.07 (dd, *J*(H,H)=7.7, 1.5 Hz, 1H; H-C(16)), 6.96 (dd, *J*(H,H)=7.3, 0.9 Hz, 1H; H-C(2)), 6.75 (d, *J*(H,H)=2.3 Hz,

2H; H-C(37), H-C(41)), 6.70 (d, *J*(H,H)=2.3 Hz, 2H; H-C(29), H-C(45)), 6.45 (t, *J*(H,H)=2.3 Hz, 1H; H-C(27)), 6.43 (t, *J*(H,H)=2.3 Hz, 1H; H-C(39)), 3.84 (s, 6H; H_3-C(48), H_3-C(49)), 3.75 (s, 6H; H_3-C(25), H_3-C(44)), 2.97 (s, 2H; H-C(11)), 2.02 (s, 6H; H_3-C(46), H_3-C(47)), 1.93 (s, 6H; H_3-C(42), H_3-C(43)) 1.51 (s, 6H; H_3-C(50), H_3-C(51)); ^{13}C NMR (101 MHz, $CDCl_3$) δ 186.7 (C(7)), 162.2 (C(9)), 161.2(C(26), C(28) or C(38), C(40)), 161.2 (C(26), C(28) or C(38), C(40)), 146.5 (C(12)), 145.3 (C(5) or C(1)), 144.1 (C(36)), 143.7 (C(24)), 142.6 (C(5) or C(1)), 140.9 (C(18)), 140.1 (C(21) or C(33)), 139.8 (C(21) or C(33)), 138.1 (C(30)), 138.0 (C(13)), 137.8 (C(8) or C(15)), 137.1 (C(19), C(23)), 135.9 (C(31), C(35)), 134.1 (C(3)), 133.0 (C(16)), 129.9 (C(14)), 129.1 (C(6)), 126.9 (C(2)), 126.4 (C(20), C(22)), 126.0 (C(32), C(34)), 125.8 (C(8) or C(15)), 124.5 (C(17)), 122.5 (C(4)), 105.6 (C(37), C(41) or (C(29), C(45)), 105.5(C(37), C(41) or (C(29), C(45)), 99.5 (C(27) or C(39)), 99.4 (C(27) or C(39)), 55.6 (C(25), C(44) or C(48), C(49)), 55.5 (C(25), C(44) or C(48), C(49)), 49.5 (C(11)), 49.2 (C(10)), 26.7 (C(50), C(51)), 21.2 (C(46), C(47)), 21.0 (C(42), C(43)); UV-Vis ($CHCl_3$): λ_{max} (ε) = 268 (55,500 l mol^{-1} cm^{-1}), 467 nm (8,200 l mol^{-1} cm^{-1}).

Synthesis of 7-(3',5'-Dimethoxy-3,5-dimethyl-[1,1'-biphenyl]-4-yl)-2-(6-(3',5'-dimethoxy-3,5-dimethyl-[1,1'-biphenyl]-4-yl)-2,2-dimethyl-2,3-dihydro-1*H*-inden-1-ylidene)benzo[*b*]thiophen-3(2*H*)-one (2)

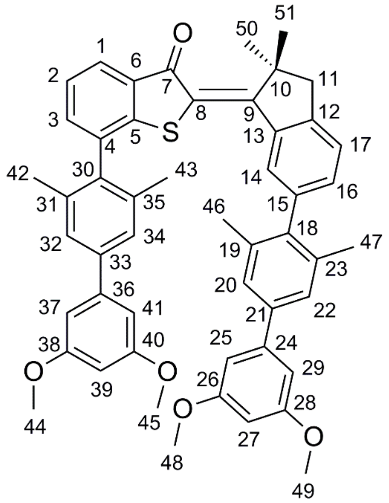

Z isomer

A flame-dried Schlenk tube was charged with compound **14** (1.00 eq, 20 mg, 0.04 mmol), (3',5'-dimethoxy-3,5-dimethyl-[1,1'-biphenyl]-4-yl)boronic acid (**9**) (4.00 eq, 51 mg, 0.18 mmol), and K_3PO_4 (6.00 eq, 57 mg, 0.26 mmol). The tube was evacuated and backfilled with argon (3 cycles). Toluene (120 µL), ethanol (120 µL), and H_2O (40 µL) were added. Argon was bubbled through the mixture for 30 min before sSPhos Pd G2 (2.90 mg, 0.35 nmol, 8 mol%) was added. The reaction mixture was stirred for 18 h at 100 °C and then was allowed to cool down to 23 °C. A saturated ammonium chloride solution (20 mL) was added. The aqueous phase was extracted with ethyl acetate (3 x 50 mL), the organic phases

were separated and dried over Na_2SO_4. The solvent was removed *in vacuo*. The crude product was purified by column chromatography (SiO_2, *i*Hex/EtOAc 9:1) followed by NP-HPLC (SiO_2, *n*-Heptane/*i*-Propanol 98.5:1.5; t_R = 5.5 min., oven temperature 40 °C) to afford *Z*-**2** (26 mg, 76%) as a yellow solid.

mp.: 129 °C; TLC (SiO_2, *i*Hex/EtOAc 8:2 v/v): R_f= 0.43;

Z isomer:

^{1}H NMR (400 MHz, $CDCl_3$) δ 7.87 (dd, *J*(H,H)=7.5, 1.5 Hz, 1H; H-C(1)), 7.73 (dd, *J*(H,H)=1.4, 0.6 Hz, 1H; H-C(14)), 7.35 (t, *J*(H,H)=7.4 Hz, 1H; H-C(2)), 7.33–7.29 (m, 4H; H-C(3), H-C(17), H-C(32), H-C(34)), 7.14 (dd, *J*(H,H)=7.6, 1.4 Hz, 1H; H-C(16)), 7.24 (s, 2H; H-C(20), H-C(22)), 6.73 (d, *J*(H,H)=2.3 Hz, 2H; H-C(37), H-C(41)), 6.67 (d, *J*(H,H)=2.3 Hz, 2H; H-C(25), H-C(29)), 6.45 (t, *J*(H,H)=2.3 Hz, 1H; H-C(27)), 6.43 (t, *J*(H,H)=2.2 Hz, 1H; H-C(39)), 3.82 (s, 6H; H_3-C(48), H_3-C(49)), 3.76 (s, 6H; H_3-C(44), H_3-C(45)), 3.11 (s, 2H; H_2-C(11)), 2.07 (s, 6H; H_3-C(42), H_3-C(43)), 2.05 (s, 6H; H_3-C(46), H_3-C(47)), 1.70 (s, 6H; H_3-C(50), H_3-C(51)); ^{13}C NMR (101 MHz, $CDCl_3$) δ 188.5 (C(7)), 164.2 (C(9)), 161.2 (C(26), C(28), C(38), C(40)), 148.5 (C(12)), 145.2 (C(5)), 143.5 (C(36)), 143.4 (C(24)), 141.2 (C(33)), 140.7 (C(18)), 140.5 (C(13)), 140.4 (C(21)), 139.3 (C(15)), 137.1*, 137.1*, 136.8 (C(30)), 136.3*, 135.0 (C(3)), 133.0 (C(16)), 132.7*, 128.7 (C(14)), 126.8 (C(32), C(34)), 126.6 (C(20), C(22)), 125.8 (C(2)), 125.4 (C(1)), 125.3 (C(17)), 105.6 (C(25), C(29)), 105.6 (C(37), C(41)), 99.7 (C(27), C(39)), 55.7 (C(48), C(49)), 55.6 (C(44), C(45)), 51.4 (C(11)), 49.3 (C(10)), 25.9 (C(50), C(51)), 21.3 (C(46), C(47)), 20.7 (C(42), C(43)); IR ($\tilde{\nu}$): 2991w, 2931w, 2836w, 1663m, 1594s, 1572m, 1457m, 1428m, 1407m ,1388m, 1355m, 1294w, 1267m, 1203s, 1178w, 1153vs, 1119w, 1099w, 1065s, 980w, 941w, 927w, 900w, 829m, 757m, 725w, 693w, 658w cm^{-1}; UV-Vis ($CHCl_3$): λ_{max} (ε) = 266 (45,000 l mol^{-1} cm^{-1}), 334 (11,600 l mol^{-1} cm^{-1}), 457 nm (11,100 l mol^{-1} cm^{-1}); HREI-MS (*m/z*): $[M]^+$ calcd. for $C_{51}H_{48}O_5S$, 772,3222, found: 772,3203; analysis (calcd., found for $C_{51}H_{48}O_5S$): C (79.24, 79.46), H (6.26, 6.55), S (4.15, 3.81).

E isomer:

^{1}H NMR (600 MHz, $CDCl_3$) δ 8.80 (d, *J*(H,H)=1.4 Hz, 1H; H-C(14)), 7.86–7.81 (m, 1H; H-C(1)), 7.37 (s, 2H; H-C(32), H-C(34)), 7.32 (s, 2H; H-C(20), H-C(22)), 7.31-7.29 (m, 3H; H-C(2), H-C(3), H-C(17)), 7.17 (dd, *J*(H,H)=7.6, 1.5 Hz, 1H; H-C(16)), 6.81 (d, *J*(H,H)=2.3 Hz, 2H; H-C(37), H-C(41)), 6.77 (d, *J*(H,H)=2.4 Hz, 2H; H-C(25), H-C(29)), 6.48 (t, *J*(H,H)=2.3 Hz, 1H; H-C(39)), 6.46 (t, *J*(H,H)=2.3 Hz, 1H; H-C(27)), 3.87 (s, 6H; H_3-C(44), H_3-C(45)), 3.86 (s, 6H; H_3-C(48), H_3-C(49)), 3.01 (s, 2H; H_2-C(11)), 2.19 (s, 6H; H_3-C(46), H_3-C(47)), 2.11 (s, 6H; H_3-C(42), H_3-C(43)); ^{13}C NMR (151 MHz, $CDCl_3$) δ 188.1 (C(7)), 163.4 (C(9)), 161.2 (C(26), C(28), C(38), C(40)), 146.9 (C(12)), 144.0*, 143.8*, 141.4*, 141.0 (C(33)), 140.9*, 140.0 (C(21)), 138.6*, 138.5*, 137.2*, 136.7*, 136.8*, 135.8*, 135.0 (C(3)), 132.7 (C(16)), 132.7*, 129.5 (C(14)), 127.4*, 126.6 (C(32), C(34)), 126.4 (C(20), C(22)), 125.6 (C(2)), 125.4 (C(1)), 124.8 (C(17)), 105.5 (C(37), C(41), C(25), C(29)), 99.6 (C(27)), 99.4

(C(39)), 55.6 (C(44), C(45), C(48), C(49)), 49.4 (C(11)), 49.2 (C(10)), 26.9 (C(50), C(51)), 21.2 (C(46), C(47)), 20.7 (C(42), C(43)); UV,Vis ($CHCl_3$): λ_{max} (ε) = 265 (42,600 l mol^{-1} cm^{-1}), 336 (12,700 l mol^{-1} cm^{-1}), 464 nm (7,900 l mol^{-1} cm^{-1}).

* could not be assigned unambiguously

Synthesis of 4-Bromobenzo[*b*]thiophen-3(2*H*)-one (3)

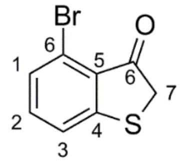

Thionyl chloride (9.00 eq, 5.32 mL, 72.84 mmol) was added to 2-((3-bromophenyl)thio)acetic acid (**11**) (2 g, 8.09 mmol) and the reaction mixture was stirred for 1 h at 80 °C. Unreacted thionyl chloride was removed *in vacuo* at 50 °C. 1,2-Dichloroethane (15 mL) was added to the residual acid chlorid. The reaction mixture was cooled to 0 °C and $AlCl_3$ (7.00 eq, 7.56 g, 56.7 mmol) was added in portions over a period of 2 min at 0 °C under vigorous stirring. The reaction mixture was stirred for 30 min at 0 °C and for 2 h at 23 °C. The suspension was poured on ice water (150 mL) and the resulting aqueous phase was extracted with CH_2Cl_2 (3 x 250 mL). The organic phases were separated, dried over Na_2SO_4, and the solvent removed *in vacuo*. The ring closed bromobenzothiophenone was obtained as a mixture of regioisomers (1.67 g, 88% combined yield) and pure **3** was isolated as a white solid after column chromatography (SiO_2, *n*-pentane/Et_2O 95:5).

TLC (SiO_2, *n*-Pentane:EtOAc, 95:5 v/v): R_f= 0.40 ; ^{1}H NMR (400 MHz, CD_2Cl_2) δ 7.41 (dd, *J*(H,H)=2.8, 1.4, 1H, H-C(1)), 7.39 (dd, *J*(H,H)=3.8, 1.4, 1H, H-C(3)), 7.35 (m, 1H, H-C(2)), 3.85 (s, 2H, H-C(7)).; ^{13}C NMR (101 MHz, CD_2Cl_2) δ 197.63 (C(6)), 158.05 (C(4)), 136.04 (C(2)), 130.74 (C(3)), 127.95 (C(5)), 124.42 (C(1)), 122.90 (C(6)), 40.57 (C(7)). HREI-MS (*m/z*): $[M]^+$ calcd. $C_8H_5{}^{79}BrOS$, 227.9244; found: 227.9245.

Synthesis of 6-Bromo-2,2-dimethyl-2,3-dihydro-1*H*-inden-1-one (4)

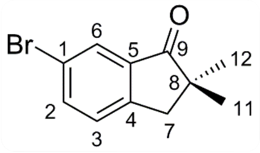

A suspension of sodium hydride (60% dispersion in mineral oil) (4.00 eq, 255 mg, 6.39 mmol) in 1,2-dimethoxyethan (4.6 mL) was cooled to 0 °C. 6-Bromo-1-indanone (1.00 eq, 337 mg, 1.60 mmol) was added and the suspension was stirred for 2 h at 0 °C. Iodomethane (4.00 eq, 400 µL, 6.39 mmol) was then added dropwise at 0 °C and the reaction mixture was stirred for 1 h at 0 °C and 20 h at 23 °C. H_2O (20 mL) was added and the aqueous phase was extracted with ethyl acetate (3 x 150 mL). The organic phases were separated and dried over Na_2SO_4. The solvent was removed *in vacuo* and the crude product was purified by column chromatography (SiO_2, *i*Hex/EtOAc 85:15) to afford **4** (336 mg, 88%) as a white solid.

mp: 45 °C; TLC (SiO_2, *i*Hex:EtOAc, 85:15 v/v): R_f= 0.7; ^{1}H NMR (600 MHz, $CDCl_3$): δ 7.88 (d, *J*(H,H)= 1.8 Hz, 1H; H-C(6)), 7.69 (dd, *J*(H,H)= 8.1, 1.9 Hz, 1 H, H-C(2)), 7.31 (d, *J*(H,H)= 8.1 Hz, 1 H, H-C(3)), 2.94 (s, 2 H, H_2-C(7)), 1.24 (s, 6 H, H-C(10), H_3-C(11)); ^{13}C NMR (150 MHz, $CDCl_3$): δ 210.0 (C(9)), 150.8 (C(4)), 137.7 (C(2)), 137.4 (C(5)), 128.4 (C(9)), 127.6 (C(2)), 121.7 (C(1)), 46.3 (C(8)), 42.6 (C(7)), 25.4 (C(11), C(12)); IR (ṽ): 3408w, 3084w, 3060w, 3030w, 2956m, 2923m, 2898w, 2360w, 1931w, 1782w, 1772w, 1710vs, 1640w, 1596m, 1538w, 1466s, 1435s, 1414s, 1380m, 1361m, 1338w, 1288m, 1254s, 1220m, 1189s, 1172vs, 1116s, 1094m, 1052m, 1002m, 912w, 887s, 863m, 818vs, 768s, 727s, 676w, 664w. cm^{-1}; HREI-MS (*m/z*): $[M]^+$ calcd. $C_{11}H_{11}{}^{79}BrO$, 237.9993; found: 237.9995 .

Synthesis of 4-Bromo-2-(6-bromo-2,2-dimethyl-2,3-dihydro-1*H*-inden-1-ylidene)benzo[*b*]thiophen-3(2*H*)-one (5)

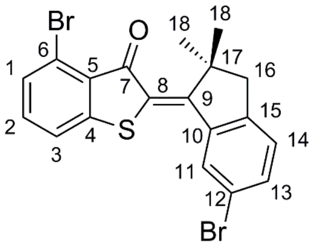

Z isomer

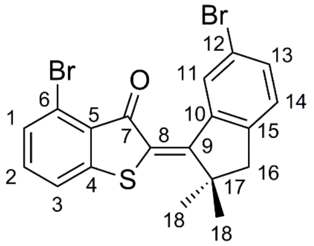

E isomer

In a flame-dried Schlenk tube 4-bromobenzo[*b*]thiophen-3(2*H*)-one (**3**) (1.00 eq, 163 mg, 0.71 mmol) was dissolved in dry THF (550 µL) under N_2 atmosphere and cooled to 0 °C. BCl_3 (1 M in CH_2Cl_2) (5.00 eq, 3.56 mL, 3.56 mmol) was added dropwise at 0 °C. 6-Bromo-2,2-dimethyl-2,3-dihydro-1*H*-inden-1-one (**4**) (1.00 eq, 170 mg, 0.71 mmol) was added and the reaction mixture was stirred for 20 min at 0 °C. Water (20 mL) was added and the aqueous phase was extracted with ethyl acetate (3 x 50 mL). The organic phases were separated and dried over Na_2SO_4. The solvent was removed *in vacuo* and the crude product was purified by column chromatography (SiO_2, *i*Hex/EtOAc 95:5) to afford **5** as a mixture of *E* and *Z* isomers (combined isomers 240 mg, 75%) as yellow solid.

mp: 189 °C;

Z isomer:

TLC (SiO_2, *i*Hex:EtOAc, 98:2 v/v): R_f= 0.46; ^{1}H NMR (400 MHz, $CDCl_3$) δ 8.19 (d, *J*(H,H)=1.7 Hz, 1H; H-C(11)), 7.49 (dd, *J*(H,H)=8.0, 1.7 Hz, 1H), 7.45 (dd, *J*(H,H)=7.7, 1.0 Hz, 1H; H-C(1 or 3)), 7.41 (dd, *J*(H,H)=7.7, 1.0 Hz, 1H; H-C(1 or 3)), 7.33 (t, *J*(H,H)=7.7 Hz, 1H; H-C(2)), 7.17 (d, *J*(H,H)=8.0, 1H; H-C(14)), 3.0 (s, 2H; H-C(16)), 1.62 (s, 6H; H-C(18)). ^{13}C NMR (151 MHz, $CDCl_3$) δ 185.6 (C(7)), 162.2 (C(9)), 148.58*, 147.72*, 142.3 (C(12)), 134.6 (C(2)), 134.1 (C(13)), 131.1 (C(1 or 3)), 130.8 (C(11)), 127.92*, 126.9 (C(14)), 126.44*, 123.02*, 122.7 (C(1 or 3)), 120.98*, 51.1 (C(16)), 49.4 (C(17)), 25.64 (C(18));

* signals could not be assigned unambiguously

E isomer:

TLC (SiO_2, *i*Hex:EtOAc, 98:2 v/v): R_f= 0.32; ^{1}H NMR (599 MHz, $CDCl_3$) δ = 9.12 (d, *J*(H,H)=1.6 Hz, 1H; H-C(11)), 7.46 (dd, *J*(H,H)=8.0, 1.6 Hz, 1H; H-C(13)), 7.43 (dd, *J*(H,H)=7.7, 1.0 Hz, 1H; H-C(1 or 3)), 7.37 (dd, *J*(H,H)=7.7, 1.0 Hz, 1H; H-C(1 or 3)), 7.32 (t, *J*(H,H)=7.7 Hz, 1H; H-C(2)), 7.14 (d, *J*(H,H)=8.0 Hz, 1H; H-C(14)), 2.91 (s, 2H; H-C(16)), 1.48 (s, 6H; H-C(18)). ^{13}C NMR (151 MHz, $CDCl_3$) δ 185.7 (C(7)), 161.3 (C(9)), 147.2 (C(4 or 6)), 146.9 (C(15)), 139.9 (C(12)), 134.6 (C(2)),

134.5 (C(13)), 131.5 (C(11)), 130.8 (C(1 or 3)), 128. (C(5)), 127.5 (C(8)), 126.3 (C())14, 123.4 (C(4 or 6)), 122. (C(1 or 3)), 120.3 (C(10)), 49.5 (C(17)), 48.9 (C(16)), 26.7 (C(18)); IR (ṽ): 3322w, 3122w, 3053w, 3015w, 2953m, 2922m, 2852m, 2363w, 2336w, 1941w, 1926w, 1895w, 1772w, 1717w, 1700w, 1664vs, 1574vs, 1560s, 1514vs, 1458s, 1438vs, 1416m, 1405m, 1376m, 1307w, 1272m, 1231vs, 1198m, 1178s, 1159m, 1116m, 1096m, 1071s, 1045s, 1014m, 994m, 974s, 939m, 906m, 886w, 865m, 856m, 844w, 806m, 770vs, 724m, 702w, 678m, 655w cm^{-1}; HREI-MS (*m/z*): $[M]^+$ calcd. for $C_{19}H_{14}{}^{81}Br_2OS$, 449.9112; found: 449.9078; calcd. for $C_{19}H_{14}{}^{79}Br_2OS$, 447.9132, found: 447.9129; analysis (calcd., found for $C_{19}H_{14}Br_2OS$): C (50.69, 50.80), H (3.13, 3.24), S (7.12, 7.15);.

Synthesis of 4-Bromo-3',5'-dimethoxy-3,5-dimethyl-1,1'-biphenyl (8)

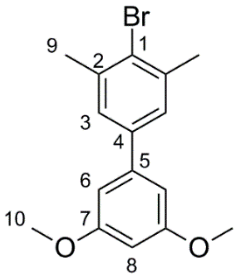

A flame-dried Schlenk tube was charged with 2,5-dibromoxylene (**6**) (1.00 eq, 750 mg, 2.84 mmol), 3,5-dimethoxy-phenylboronic acid (**7**) (1.10 eq, 569 mg, 3.13 mmol), and K_2CO_3 (3.00 eq, 1.18 g, 8.52 mmol). The tube was evacuated and backfilled with argon (3 cycles) before 1,2-dimethoxyethan (11.25 mL) and H_2O (11.25 mL) were added. Argon was bubbled through the mixture for 30 min before $Pd(PPh_3)_4$ (164 mg, 0.14 mmol, 5 mol%) was added. The reaction mixture was stirred for 13 h at 84 °C and was then allowed to cool down to 23 °C. A saturated ammonium chloride solution (100 mL) was added. The aqueous phase was extracted with ethyl acetate (3 x 250 mL), the organic phases were separated and dried over Na_2SO_4. The solvent was removed *in vacuo*. The crude product was purified by column chromatography (SiO_2, *i*Hex/EtOAc 99:1) to afford **8** (751 mg, 82%) as a white solid.

mp: 92 °C; TLC (SiO_2, *i*Hex/EtOAc 99:1 v/v): R_f=0.32; ^{1}H NMR (400 MHz, $CDCl_3$): δ 7.28 (m, 2H; H-C(3)), 6.66 (d, *J*(H,H)=2.3 Hz, 2H; H-C(6)), 6.48 (t, *J*(H,H)=2.3 Hz, 1H; H-C(8)), 3.83 (s, 6H; H_3-C(10)), 2.45 (s, 6H; H_3-C(9); ^{13}C NMR (101 MHz, $CDCl_3$): δ 161.3 (C(7)), 142.8 (C(5)), 139.9 (C(4)), 138.8 (C(2)), 127.1 (C(3)), 105.5 (C(6)), 99.6 (C(8)), 55.7 (C(10)), 24.2 (C(9)), C(1) not seen due to overlap;)); IR (ṽ): 2996w, 2966w, 2935w, 2843w, 2360w, 2335w, 2102w, 1653w, 1609s, 1588vs, 1573vs, 1453s, 1433s, 1406m, 1383m, 1378m, 1345vs, 1277s, 1246w, 1199vs, 1180m, 1159vs, 1064vs, 1055vs, 1031s, 1015s, 1000m, 991m, 943s, 883m, 849s, 838vs, 808s, 739w, 710w, 692s cm^{-1}; HREI-MS (*m/z*): $[M]^+$ calcd. for $C_{16}H_{17}{}^{79}BrO_2$, 320.0412, found: 320.0405; $[M]^+$ calcd. for $C_{16}H_{17}{}^{81}BrO_2$, 322.0391, found: 322.0388; analysis (calcd., found for $C_{16}H_{17}BrO_2$): C (59.83, 59.82), H (5.33, 5.31).

Synthesis of (3',5'-Dimethoxy-3,5-dimethyl-[1,1'-biphenyl]-4-yl)boronic acid (9)

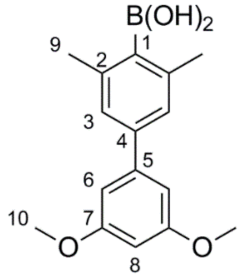

A solution of 4-bromo-3',5'-dimethoxy-3,5-dimethyl-1,1'-biphenyl (**8**) (1.00 eq, 688 mg, 2.14 mmol) in dry THF (7 mL) was cooled to -78 °C under N_2. SecBuLi (1.4 M in cyclohexane) (1.15 eq, 1.97 mL, 2.46 mmol) was added dropwise, and the solution was stirred at -78 °C for 30 min, before trimethyl borate (1.20 eq, 286 µL, 2.57 mmol) was added. The solution was stirred for 15 min at -78 °C, then the cooling bath was removed and the solution was stirred for 30 min at 23 °C. Aq. HCl (1M) was added (30 mL) and the aqueous phase was extracted with Et_2O (3 x 100 mL). The organic phases were separated and dried over Na_2SO_4. The solvent was removed *in vacuo*. The crude product was used without further purification to afford **9** (539 mg, 88%) as a white solid.

mp: 169 °C; ^{1}H NMR (400 MHz, acetone-d_6): δ 7.22 (s, 2H; H-C(3)), 6.78 (d, *J*(H,H)=2.3 Hz, 2H; H-C(6)), 6.47 (t, *J*(H,H)=2.2 Hz, 1H; H-C(8)), 3.84 (s, 6H; H_3-C(10)), 2.42 (s, 6H; H_3-C(9)); ^{13}C NMR (100 MHz, acetone-d_6): δ 162.2 (C(7)), 144.4 (C(5)), 141.2 (C(4)), 140.4 (C(2)), 138.9 (C(1)), 125.4 (C(3)), 105.8 (C(6)), 99.7 (C(8)), 55.7 (C(10)), 22.5 (C(9));)); IR (ṽ): 3220m, 3000w, 2940w, 2840w, 2361w, 2338w, 1700w, 1594vs, 1559m, 1456s, 1430s, 1387s, 1352vs, 1267m, 1203s, 1153vs, 1117w, 1064s, 992w, 939w, 925w, 882w, 832m, 755w, 732w, 694w cm^{-1}; HRESI-MS (*m/z*): $[M]^-$ calcd. for $C_{16}H_{18}BO_4^-$, 285.1304, found: 285.1305.

Synthesis of 2-(2,4,7-Trinitro-9*H*-fluoren-9-ylidene)malononitrile (10)

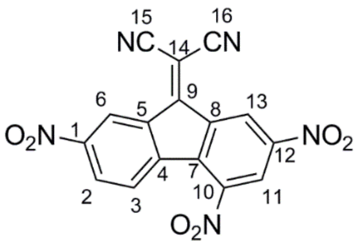

2,4,7-Trinitro-*9H*-fluoren-9-one (1.00 eq, 215 mg, 0.68 mmol) was dissolved in DMF (1.1 mL) and the solution was heated to 55 °C. Malononitrile (2.20 eq, 99 mg, 1.5 mmol) was added in portions and the solution was stirred for 2 h at 55 °C. Afterwards *i*-propanol (4 mL) was added and the solution was allowed to crystallize at 4 °C. The crude product was filtered off, washed with ethanol and recrystallized from acetonitrile to afford **10** (104 mg, 42%) as dark brown crystals.

mp: 266 °C; TLC (SiO_2, *i*Hex/EtOAc 8:2 v/v): R_f= 0.46; ^{1}H NMR(400 MHz, $CDCl_3$): δ 9.67 (d, *J*(H,H)=1.8 Hz, 1H; H-C(13)), 9.49 (d, *J*(H,H)=2.3 Hz, 1H; H-C(6)), 8.93 (d, *J*(H,H)=1.9 Hz, 1H; H-C(11)), 8.56 (dd, *J*(H,H)=8.7, 2.1 Hz, 1H; H-C(2)), 8.21 (dd, *J*(H,H)=8.7, 0.6 Hz, 1H; H-C(3)); ^{13}C NMR(151 MHz, $CDCl_3$): δ153.9 (C(9)), 150.2 (C(1)), 148.8 (C(12)), 145.7 (C(10)), 140.4 (C(4)), 138.7 (C(8)), 136.6 (C(5) or C(7)), 136.5 (C(5) or C(7)), 130.4 (C(2)), 127.8 (C(3)), 125.1 (C(11)), 124.4 (C(13)), 121.9 (C(6)), 111.8 (C(15) or C(16)), 111.6 (C(15) or C(16)), 83.6 (C(14)); HREI-MS (*m/z*): $[M]^+$ calcd. for $C_{16}H_5N_5O_6$, 363.0240, found: 363.0233; ; analysis (calcd., found for $C_{16}H_5N_5O_6$): C (52.91, 52.67), H (1.39, 1.49), N (19.28, 19.15).

Synthesis of 2-((3-Bromophenyl)thio)acetic acid (11)

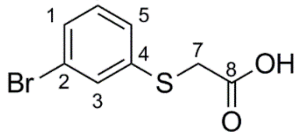

Bromoacetic acid (1.10 eq, 1.43 g, 10.30 mmol) was dissolved in acetone (22 mL). The solution was then cooled to 0 °C and K_2CO_3 (3.0 eq, 3.87 g, 28 mmol) was added. The slurry suspension was vigorously stirred for 15 min at 0 °C and 3-bromothiophenol (1.00 eq, 1.10 mL, 9.35 mmol) was added. The suspension was stirred for 20 min at 0 °C and 3 h at 23 °C and then acidified with aq. HCl (2M) to pH=2. The aqueous phase was extracted with CH_2Cl_2 (3 x 250 mL). The organic phases were separated and dried over Na_2SO_4. The solvent was removed *in vacuo* to afford **11** (2.28 g, quant.) as a white solid. The crude product was used without further purification.

mp: 86 °C; ^{1}H NMR (400 MHz, DMSO-d_6) δ 7.52 (t, *J*(H,H)=1.9 Hz, 1H; H-C(3)), 7.37 (dt, *J*(H,H)=7.8, 1.5 Hz, 1H; H-C(5)), 7.32 (dt, *J*(H,H)= 8.0, 1.3 Hz, 1H; H-C(1)), 7.26 (t, *J*(H,H)=7.8 Hz, 1H; H-C(6)), 3.86 (s, 2H; H-C((7)); ^{13}C NMR (101 MHz, DMSO-d_6) δ 170.3 (C(8)), 138.8 (C(4)), 130.8 (C(6)), 129.3 (C(3)), 128.5 (C(5)), 126.4 (C(1)), 122.1 (C(2)), 34.6 (C(7)); IR (ṽ): 3055w, 2999w, 2955w, 2919m, 2718w, 2691w, 2583m, 2497w, 2362w, 2341w, 2218w, 2110w, 1954w, 1862w, 1826w, 1768w, 1698vs, 1566s, 1553s, 1501w, 1462m, 1424s, 1390s, 1307s, 1259m, 1200vs, 1169m, 1101w, 1085m, 1071m, 989w, 955w, 906s, 893vs, 878s, 815m, 774vs, 750vs, 677s, 666m cm^{-1}; HREI-MS (*m/z*): $[M]^+$ calcd. $C_8H_7BrO_2S$, 245.9350; found 245.9344.

Synthesis of 2-((2-Bromophenyl)thio)acetic acid (12)

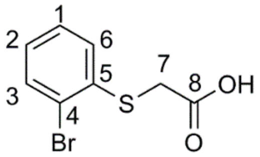

Bromoacetic acid (1.10 eq, 2.64 g, 18.98 mmol) was dissolved in acetone (40 mL). The solution was then cooled to 0 °C and K_2CO_3 (3.0 eq, 7.15 g, 51.76 mmol) was added. The slurry suspension was vigorously stirred for 15 min at 0 °C and 2-bromothiophenol (1.00 eq, 2.00 mL, 17.25 mmol) was added. The suspension was stirred for 20 min at 0 °C and 3 h at 23 °C and then acidified with aq. HCl (2M) to pH=2. The aqueous phase was extracted with CH_2Cl_2 (3 x 400 mL). The organic phases were separated and dried over Na_2SO_4. The solvent was removed *in vacuo* to afford **12** (4.35 g, quant.) as a white solid. The crude product was used without further purification.

mp: 101 °C; ^{1}H NMR (400 MHz, DMSO-d_6) δ 7.60 (dd, *J*(H,H)=7.9, 1.3 Hz, 1H; H-C(3)), 7.37 (ddd, *J*(H,H)=7.9, 7.3, 1.3 Hz,1H; H-C(1)), 7.30 (dd, *J*(H,H)=8.0, 1.7 Hz, 1H; H-C(6)), 7.10 (ddd, *J*(H,H)=7.9, 7.2, 1.6 Hz, 1H; H-C(2)), 3.89(s, 2H; H_2-C(7)); ^{13}C NMR (101 MHz, DMSO-d_6) δ 170.1 (C(8)), 137.2 (C(5)), 132.6 (C(3)), 128.3 (C(1)), 126.8 (C(6)), 126.7 (C(2)), 120.8 (C(4)), 34.2 (C(7)); IR (ṽ): 2988w, 2911w, 2688w, 2609w, 2577w, 2499w, 2359w, 1957w, 1917w, 1819w, 1699s, 1600m, 1569m, 1557m, 1449m, 1426vs, 1404m, 1384s, 1315s, 1260m, 1254m, 1230w, 1198vs, 1161m, 1120m, 1108m, 1039w, 1019s, 939w, 899s, 891vs, 851m, 809m, 749vs, 713s, 671m, 668m cm^{-1}; HREI-MS (*m/z*): $[M]^+$ calcd. for $C_8H_7BrO_2S$, 245.9350, found 245.9347.

Synthesis of 7-Bromobenzo[*b*]thiophen-3(2*H*)-one (13)

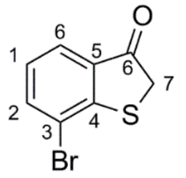

Thionyl chloride (9.00 eq, 1.33 mL, 18.18 mmol) was added to 2-((2-bromophenyl)thio)acetic acid (**12**) (1.00 eq, 500 mg, 2.02 mmol) and the reaction mixture was stirred for 1 h at 80 °C. Unreacted thionyl chloride was removed *in vacuo* at 50 °C and 1,2-dichloroethane (3.5 mL) was added to the residual acid chloride. The reaction mixture was cooled to 0 °C and $AlCl_3$ (5.00 eq, 1.35 g, 10.10 mmol) was added in portions over a period of 2 min at 0 °C under vigorous stirring. The reaction mixture was stirred for 30 min at 0 °C and for 2 h at 23 °C. The suspension was poured on ice water (150 mL) and the aqueous phase was extracted with CH_2Cl_2 (3 x 200 mL). The organic phases were separated, dried over Na_2SO_4 and the solvent was removed *in vacuo* to afford **13** (456 mg, quant.) as a pink solid. The crude product was used without further purification.

^{1}H NMR(400 MHz, CD_2Cl_2-d2) δ 7.73 (dd, J=7.7, 1.1, 1H, H-C(2)), 7.70 (dd, J=7.7, 1.1, 1H, H-C(6)), 7.14 (t, J=7.7, 1H, H-C(1)), 3.84 (s, 2H, H-C(7)), ^{13}C NMR (101 MHz, CD_2Cl_2) δ 199.54 (C(6)), 155.51 (C(5)), 138.36 (C(2)), 133.41 (C(4)), 126.56 (C(1)), 125.42 (C(6)), 118.93 (C(3)), 40.63 (C(7)); HREI-MS (*m/z*): $[M]^+$ calcd. for $C_8H_5Br^{79}OS$, 227.92444, found: 227.9249; calcd. for $C_8H_5Br^{81}OS$, 229.9224, found: 229.9234.

Synthesis of 7-Bromo-2-(6-bromo-2,2-dimethyl-2,3-dihydro-1*H*-inden-1-ylidene)benzo[*b*]thiophen-3(2*H*)-one (14)

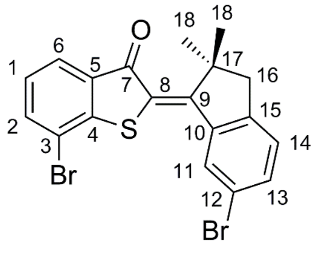

Z isomer

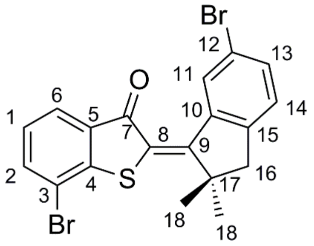

E isomer

In a flame-dried Schlenk tube 7-bromobenzo[*b*]thiophen-3(2*H*)-one (**13**) (1.00 eq, 313 mg, 1.37 mmol) was dissolved in dry THF (500 µL) under N_2 atmosphere and cooled to 0 °C. BCl_3 (1M in CH_2Cl_2)

(5.00 eq, 6.83 mL, 6.83 mmol) was added dropwise at 0 °C. 6-Bromo-2,2-dimethyl-2,3-dihydro-1*H*-inden-1-one (**4**) (1.00 eq, 327 mg, 1.37 mmol) was added and the reaction mixture was stirred for 20 min at 0 °C and 4 h at 23 °C. Water (30 mL) was added and the aqueous phase was extracted with ethyl acetate (3 x 50 mL). The organic phases were separated and dried over Na_2SO_4. The solvent was removed *in vacuo* and the crude product was purified by column chromatography (SiO_2, *i*Hex → *i*Hex/EtOAc 99:1) to afford a mixture of *Z*-**14** and *E*-**14** (combined isomers 353 mg, 57%) as yellow, microcrystalline solid.

mp: 179 °C; TLC (SiO_2, *i*Hex/EtOAc 9:1 v/v): R_f= 0.45;

Z isomer:

^{1}H NMR (800 MHz, $CDCl_3$) δ 8.31 (d, *J*(H,H)= 1.7 Hz, 1H; H-C(11), 7.83 (dd, *J*(H,H)=7.7, 1.1 Hz, 1H; H-C(6)), 7.72 (dd, *J*(H,H)=7.7, 1.1 Hz, 1H; H-C(2)), 7.72 (dd, *J*(H,H)=7.7, 1.1 Hz, 1H; H-C(2)), 7.54 (dd, *J*(H,H)=8.0, 1.7 Hz, 1H; H-C(13)), 7.20 (t, *J*(H,H)=7.7 Hz, 1H; H-C(1)), 7.20 (d, *J*(H,H)=8.1 Hz 1H; H-C(14)), 7.16 (d, *J*(H,H)=8.0 Hz, 1H; H-C(14)), 3.04 (s, 2H; H-C(16)), 1.63 (s, 3H; H_3-C(18)); ^{13}C NMR (201 MHz, $CDCl_3$) δ 187.4 (C(7)), 163.6 (C(9)), 148.8 (C(15)), 146.2 (C(4)), 142.0 (C(12)), 137.1 (C(2)), 134.4 (C(13)), 133.9 (C(5)), 131.0 (C(11)), 127.0 (C(8)), 126.9*, 126.7*, 126.4*, 126.3 (C(14)), 125.4 (C(6)), 121.1 (C(10)), 117.5* (C(3)), 117.3* (C(3)), 51.0 (C(16)), 49.3 (C(17)), 25. (C(18)); IR (ṽ): 3309w, 3125w, 3059w, 3027w, 2983w, 2951w, 2920w, 2858w, 2364w, 2337w, 1927w, 1871w, 1815w, 1660vs, 1580s, 1566m, 1519s, 1503s, 1460s, 1452s, 1436s, 1409s, 1375m, 1366m, 1358m, 1306m, 1272m, 1260s, 1220m, 1200w, 1179m, 1167m., 1133m, 1105s, 1079m, 1070m, 1053w, 1037s, 996m, 982m, 953w, 940w, 924w, 900m, 867m, 849m, 814w, 798s, 746vs, 722s, 702w, 683w, 654m cm^{-1}; HREI-MS (*m/z*): $[M]^+$ calcd. for $C_{19}H_{14}{}^{79}Br_2O_1S$, 447.9132, found: 447.9141; calcd. for $C_{19}H_{14}{}^{81}Br_2O_1S$, 449.9112, found: 449.9146.

E isomer:

^{1}H NMR (800 MHz, $CDCl_3$) δ 9.24 (d, *J*(H,H)=1.7 Hz, 1H; H-C(11)), 7.89 (dd, *J*(H,H)=7.7, 1.1 Hz, 1H; H-C(6)), 7.72 (dd, *J*(H,H)=7.7, 1.1 Hz, 1H; H-C(2)), 7.50 (dd, *J*(H,H)=8.0, 1.9 Hz, 1H; H-C(13)), 7.19 (t, *J*(H,H)=7.7 Hz, 1H; H-C(1)), 7.16 (d, *J*(H,H)=8.1 Hz 1H; H-C(14)), 7.16 (d, *J*(H,H)=8.0 Hz, 1H; H-C(14)), 2.97 (s, 2H; H-C(16)), 1.57 (s, 3H; H_3-C(18)); ^{13}C NMR (201 MHz, $CDCl_3$) δ 187.5 (C(7)), 163.0 (C(9)), 147.2 (C(15)), 145.6 (C(4)), 139.9 (C(12)), 137.2 (C(2)), 134.7 (C(13)), 134.1 (C(5)), 131.7 (C(11)), 128.1 (C(8)), 126.9*, 126.7*, 126.4*, 125.7 (C(6)), 120.2 (C(10)), 117.5* (C(3)), 117.3* (C(3)), 49.5 (C(17)), 49.0 (C(16)), 26.9 (C(18)).

* could not be assigned unambiguously

Determination of the guest binding of tweezers 1 and 2

General

The binding constants for the interaction of tweezers *E*-**1** and *Z*-**2** with **10** were determined by ^{1}H NMR titration. A modified version of the *dilution method* was performed in which the tweezers concentrations were varied and the concentration of **10** was kept constant to account for the poor solubility of the guest **10** in $CDCl_3$. Due to signal broadening at ambient temperatures ^{1}H NMR titrations were performed at 253 K.

The acquired ^{1}H NMR titration data sets were processed using the online tool *Bindfit* provided at http://www.supramolecular.org. This procedure to determine the binding stoichiometry has frequently been cited as reliable method.[4, 5] Data fitting of the NMR titrations and direct assessment of the binding stoichiometry via Job plot analysis yielded the same 1:1 ratio between tweezers and guest in the binding complex (Supplementary Figure 9). For the titration of **10** with *E*-**1** and *Z*-**2** (Supplementary Figure 10 and 12) composed screenshots from the supramolecular.org website are given in Supplementary Figure 11 and 13. The screeshots include the estimated binding constants and the corresponding errors of the fits for a 1:1 stoichiometry. Other stoichiometries were implausible due to unreasonable errors (2:1) of the fitting or noncredible binding modes (1:2).

Structural analysis of the tweezers-**10** complexes was conducted using solution NMR spectroscopy in close conjunction with theoretical descriptions.

Determination of the binding stoichiometry of 1 and 2 with 10

In order to directly determine the binding stoichiometry of tweezers *E*-**1** and *Z*-**2** with guest **10** Job plot analysis (method of continuous variation) was performed applying the procedure of *Crabtree*.[6] 11 NMR samples (500 µL) were prepared for each plot and the *E*-**1** and *Z*-**2** isomers were enriched previous to the measurements by irradiation at 435 nm and 530 nm, respectively. The total concentration of the guest **10** plus each tweezers *E*-**1** or *Z*-**2** was kept at 0.26 mM while the molar ratio of *E*-**1** and *Z*-**2** tweezers to guest were varied from 0 to 1 in 0.1 steps. ^{1}H NMR spectra of the samples were recorded on a 400 MHz NMR spectrometer at -20 °C (Supplementary Figure 9a and c), so that the results could be directly correlated with the titration experiments. In the graph $\frac{[H]}{[H]+[G]}$ was plotted against $\frac{\Delta\delta \cdot [H]}{[H]+[G]}$, with [H] as the respective *E*-**1** or *Z*-**2** tweezers concentration, [G] as the guest **10** concentration, and $\Delta\delta$ as difference of the chemical shift of the indicative proton 44 and 45 (tweezers *E*-**1**) and proton 46 and 47 (tweezers *Z*-**2**) signals.

Both Job plot analyses for the binding of tweezers *E*-**1** and *Z*-**2** with **10** showed a maximum at a mole fraction of 0.5 (Supplementary Figure 9b and d). Consequently a binding stoichiometry of 1:1 was applied in data evaluation of the NMR titration experiments.

Supplementary Note 1

Conformational Analysis

Tweezers *E*-1

E-**1** assumes a tweezers-like conformation in solution with both electron rich biphenyl units pointing in the same direction. Since we did not obtain a crystal structure of **1** the experimental assignment of *Z* or *E* configuration is not straight forward, although the positive photochromism that is observed for all HTIs gives a first indication. NOESY analysis did not allow us to measure cross-peak signals between the thioindigo- and the stilbene fragments and could therefore also not be used for an unambiguous assignment. To pinpoint the configuration of the central double bond we rely on indicative chemical shifts particularly of proton 14 (see the synthesis part of tweezers **1** for the full numbering of the atoms), which resides between the five-membered ring of the stilbene fragment and the biphenyl unit. The signal of proton 14 in the ^{1}H NMR spectrum of *E*-**1** appears as the most downfield signal and is shifted upfield upon photoisomerization. A similar behavior but to greater extend in absolute shift difference is observed for the dibrominated synthetic precursor **5**, which shows that the signal position of the corresponding proton 11 (see the synthesis part of **5** for the full numbering of the atoms) is strongly influenced by close proximity of either the sulfur atom (*Z*-**1**) or the carbonyl group (*E*-**1**) of the thioindigo fragment. Especially the magnetic anisotropic effect of the carbonyl group is responsible for the strong downfield shifting of the signal of nearby proton 11 (see Supplementary Figure 1). As we could obtain a crystal structure of *E*-**5** the corresponding ^{1}H NMR spectra and chemical shifts of proton 11 could unambiguously be assigned for both *Z*-**5** and *E*-**5**. Based on these assignments the corresponding ^{1}H NMR spectra of **1** could also be directly related to the specific configuration. In *E*-**1** the downfield shift effect of the carbonyl is strongly counteracted by the ring current of both nearby biphenyl arms (see Supplementary Figure 1c). This leads to a reduction in the overall chemical shift differences for proton 14 in *E*-**1** and *Z*-**1** but not to a reversal of the shifting trend observed for **5**. The *E*-**1** and *Z*-**1** assignment is further supported by the similar positive photochromism of **1** and **5**: the longer wavelength irradiation (530 nm) leads to formation of the thermodynamically more stable configuration with shorter wavelength absorptions, which is typically the *Z* isomer of HTI photoswitches.

Further signal analysis revealed that in the *E*-**1** most aromatic signals are shifted upfield with regard to their position in *Z*-**1**, which is to be expected if a twisted and parallel arrangement of the biphenyl units is indeed present. Only in *E*-**1** are the biphenyl units sufficiently close and in the correct spatial arrangement to affect each other by their respective ring currents. This behavior gives further evidence to the effectiveness of the intended preorganization of tweezers **1**.

Tweezers *Z*-2

For tweezers **2** a similar comparative analysis with the dibrominated precursor HTI **15** allowed to assign the *E* and *Z* configurations in solution. HTI **15** shows exactly the same behavior as **5**: strong downfield shift of the proton 11 (see the synthesis part of **15** for the full numbering of the atoms) signal in the *E* isomer and a considerable upfield shift of this signal in the *Z* isomer. Tweezers **2** follows this behavior closely the only difference being that a slight upfield shift is observed for this signal in both the *Z* and the *E* isomer (Supplementary Figure 1d).

Supplementary Note 2

Thermal stability of 1 and 2

For the determination of the thermal stability of tweezers **1** and **2** 5 mM solutions in $CDCl_3$. were prepared. The solutions were irradiated at 435 nm to obtain the thermodynamically less stable *E* isomer. The percentage of *E* isomer was determined by ^{1}H NMR spectroscopy. The shifting percentage of *E* isomer was followed by ^{1}H NMR spectroscopy in regular time intervals (e.g. 15 minutes) (see Supplementary Figure 7a for tweezers **1** and Supplementary Figure 8a for tweezers **2**). After heating the solution of *E* isomer enriched tweezers **1** to 35 °C for 2.5 h a stable ratio *Z*-**1**:*E*-**1** of 13:87 was established (see Supplementary Figure 7b), which can be translated to an energy difference of 1.16 kcal mol^{-1} between *Z*-**1** and *E*-**1**. For tweezers **2** the same procedure was applied, however at 50°C. After heating the solution to 50 °C for 2 h a stable ratio of 36:64 was established (see Supplementary Figure 8b), which can be translated to an energy difference of 0.36 kcal

The first-order rate constants for the thermal *E*/*Z* isomerization of tweezers **1** and **2** were determined from the decay kinetics of the *E* isomers during prolonged heating (Supplementary Figure 7 and 8). As the thermal isomerizations do not proceed to 100% *Z* isomer but instead lead to isomer equilibria the observed decays are composites of both isomerization processes *E* to *Z* and *vice versa* and have to be described according to equation 1:

$$\ln\left(\frac{c(E0)-c(E\text{eq})}{c(Et)-c(E\text{eq})}\right) = (k(E \to Z) + k(Z \to E))t \qquad \text{(Equation 1)}$$

with
$c(E_0)$ = initial concentration of the *E* isomer
$c(E_{eq})$ = concentration of the *E* isomer at equilibrium
$c(E_t)$ = concentration of the *E* isomer at the particular point in time
$k(E \to Z)$ = rate constant of the *E*/*Z* isomerization
$k(Z \to E)$ = rate constant of the *Z*/*E* isomerization
t = elapsed time.

The slope *m* obtained from the log-plot according to equation 1 is $m = 5.95782 \times 10^{-4}$ s^{-1} for tweezers **1** (Supplementary Figure 7) and $m = 5.84279 \times 10^{-4}$ s^{-1} for tweezers **2** (Supplementary Figure 8) and contains the rate constants for both isomerization directions. From this slope the desired rate constant of the thermal *E*/*Z* isomerization $k(E \to Z)$ can be calculated according to equation 2:

$$k(E \to Z) = \frac{m}{1+\frac{c(E\text{eq})}{c(Z\text{eq})}} \qquad \text{(Equation 2)}$$

when taking into account the law of mass action (equation 3):

$$\frac{c(E\text{eq})}{c(Z\text{eq})} = \frac{k(Z\to E)}{k(E\to Z)} \qquad \text{(Equation 3)}$$

with $c(Z_{eq})$ = concentration of the *Z* isomer at equilibrium

$c(E_{eq})$ = concentration of the *E* isomer at equilibrium

The first-order rate constant for the thermal *E*/*Z* isomerization of tweezers **1** is $k(E \to Z)$ = 5.21216 x 10^{-4} s^{-1} at 35 °C and for tweezers **2** is $k(E \to Z)$ = 3.73728 x 10^{-4} s^{-1} at 50 °C. The Gibbs energy of activation ΔG^* for the thermal *E*/*Z* isomerization can now be calculated using the Eyring equation:

$$k(E \to Z) = \frac{k_B\ T}{h}\ \mathrm{e}^{\frac{-\Delta G^*}{RT}} \qquad \text{(Equation 4)}$$

with k_B = Boltzmann constant ($1.381 \cdot 10^{-23}$ J·K^{-1})

T = temperature in K

h = Planck constant ($6.626 \cdot 10^{-34}$ J·s)

After rearranging equation 4 and insertion of the numerical values of the constants ΔG^* is given by:

$$\Delta G^* \text{ (in J mol}^{-1}) = 8.314 \cdot T \cdot \left[23.760 + \ln\left(\frac{T}{k(E\to Z)}\right)\right] \qquad \text{(Equation 5)}$$

For the thermal *E/Z* isomerization ΔG^* values of 22.7 kcal·mol^{-1} (tweezers **1**) and 24.0 kcal·mol^{-1} (tweezers **2**) were obtained.

The relative energy difference between the two isomeric states ΔG can be calculated according to:

$$-\Delta G = \ln K \cdot R \cdot T \qquad \text{(Equation 6)}$$

with K = equilibrium constant = [*Z*]/[*E*]

R = ideal gas constant (8.314 J·K^{-1}·mol^{-1})

T = temperature in K

Using K = 87/13 (tweezers **1** at 35 °C) and 64/36 (tweezers **2** at 50 °C) for the equilibrium constants at the corresponding temperatures energy differences between the *Z* and *E* isomer of 1.16 kcal·mol^{-1} for tweezers **1** and 0.37 kcal·mol^{-1} for tweezers **2** were obtained.

Supplementary Note 3

Theoretical description of molecular tweezers 1 and 2

Ground state geometries

For the low affinity isomers of the tweezers i.e. *Z*-**1** and *E*-**2** the structures were optimized on the B3LYP/6-311G(d,p) level of theory and have been confirmed to be minimum structures by frequency analysis.

For the high affinity isomers of the tweezers i.e. *E*-**1** and *Z*-**2** a conformational analysis has been conducted in advance of full optimization in order to ascertain that minima close to the global minimum are found. To this end the MM3* force field method with a Monte Carlo Multiple Minimum (MCMM) search algorithm and an energy threshold of 8 kJ/mol was employed using the MacroModel package from Schrödinger Software.[7]

The resulting minimum structures were scrutinized for conformational and energetic redundancies. The only significant degree of freedom that had to be taken into account consists of the orientation of the methoxy groups at the end of the biphenyl arms.

Only one clear global minimum structure was found for each *E*-**1** and *Z*-**2** - each for the two most different methoxy group orientations, i.e. all four methoxy groups pointing away from or all four pointing towards the tweezers. These four global minimum structures were then further optimized on the B3LYP/6-311G(d,p) level of theory and have been confirmed to be minimum structures by frequency analysis. Likewise the structures were also further optimized on the B3LYP-GD3BJ/6-311G(d,p) level of theory to test the influence of added dispersion. These latter structures provided less agreement with the experimentally obtained geometries in solution as analyzed by ^{1}H NMR spectroscopy.

For the host-guest complexes *E*-**1**·**10** and *Z*-**2**·**10** dispersive effects between host and guest have been considered using the D3 Grimme-dispersion with Becke-Johnson damping (GD3BJ). For calculations including dispersion (GD3BJ) the Gaussian16 Revision A.03[8] program package was used. Other calculations have been carried out using the Gaussian09 Revision A.02[9] program package.

Seven to ten different orientations of the guest **10** inside each *E*-**1** and *Z*-**2** were used as starting geometries obtained from a prior assembly search using the MM3* force field method with a Monte Carlo Multiple Minimum (MCMM) search algorithm and an energy threshold of 8 kJ/mol. As a result only one global minimum for the host-guest complexes was found for each tweezers.

Since frequency analysis for supramolecular binding complexes are hardly accessible, cost intensive, and often yield one or more imaginary frequencies as a result of numerical noise we omitted this step for *E*-**1**·**10** and *Z*-**2**·**10**.[10]

Based on the B3LYP/6-311G(d,p) optimized structures electrostatic potentials (ESPs) for *E*-**1** and *Z*-**2** and **10** were calculated and visualized using GaussView 5.0.8.

Additionally, ^{1}H NMR chemical shifts have been calculated for all optimized structures on the B3LYP/6-311++G(d,p) level of theory. It should be noted that although the chosen basis set is not particularly optimized for NMR shift calculations it provides good agreement with experimental data (due in part to intrinsic error compensation) within reasonable calculation times. Additionally, using this basis prevented SCF convergence issues during calculation of the ^{1}H NMR chemical shifts for the optimized structures. In the case of tweezers *E*-**1** and *Z*-**2** the ^{1}H NMR chemical shifts of both minimum structures with all four methoxy groups pointing towards and away from the tweezers were averaged as the experimental NOESY NMR spectra show a mixture of conformations.

We have also optimized the different isomers of **1** and **2** in the absence of guest molecule **10** using the B3LYP-GD3BJ/6-311G(d,p) level of theory but found less agreement with the experimental NMR data for the obtained structures. It seemed therefore clear that dispersion interactions are preeminent in the host-guest complexes and are too exaggerated in the gas phase calculations of the tweezers alone.

Supplementary Note 4

Dynamic relocation of 10 between tweezers 1 and 2

To demonstrate the reversible exchange of **10** between tweezers **1** and **2** a solution with a 1:1:0.6 stochiometry of tweezers **1** (conc. = 2.2 mM):tweezers **2** (conc. = 2.2 mM):**10** (conc. = 1.3 mM) was prepared. ^{1}H NMR spectra were recorded after irradiation at 435 nm (88% *E*-**1** and 64% of *E*-**2**) for 60 min and 530 nm (82% *Z*-**1** and 85% of *Z*-**2**) for 60 min (see Supplementary Figure 23 spectra 3 to 6, respectively). For evaluation of the guest relocation changes in the chemical shifts of proton 11 for **1** and simultaneously **2** were analyzed. Different to the proton signals of guest **10** proton 11 of the hosts is shifted significantly downfield upon host-guest complexation. Due to its isolated position in the ^{1}H NMR spectra proton 11 is therefore highly indicative for the binding state of the tweezers.

For comparison a mixture of **1** and **2** without **10** in the respective pss at 435 nm and 530 nm was examined first (see Supplementary Figure 23 spectra 1 and 2, respectively).

After the addition of **10** the 3-component solution was irradiated at 435 nm for *Z*/*E* isomerization, transforming non binding *Z*-**1** into binding *E*-**1** and vice versa for **2**. The obtained mixture contains now 1.4 equiv. *E*-**1** and 0.6 equiv. of *Z*-**2** with respect to 1 equiv. of the guest **10**. The proton 11 signal of *E*-**1** shows a drastic downfield shift correlated with the uptake of **10**. For the remaining minority species *Z*-**2** (36% of **2** remaining as *Z* isomer at 435 nm) the chemical shift of proton 11 shows also a downfield shift but to a much lesser degree compared with *E*-**1**. This shift indicates that somewhat limited binding also occurs to *Z*-**2** at this wavelength. As the binding constant of *E*-**1** is significantly higher than that of *Z*-**2** and *E*-**1** is the majority high-affinity component at 435 nm the guest molecule **10** is primarily bound by tweezers *E*-**1** at this wavelength.

Irradiation at 530 nm leads to a pss containing the maximum concentration of binding *Z*-**2** (82%) and minimum concentration of binding *E*-**1** (15%). The obtained mixture contains now 0.3 equiv. *E*-**1** and 1.4 equiv. of *Z*-**2** with respect to 1 equiv. of the guest **10**. A clearly more pronounced downfield shift of the proton 11 signal of tweezers *Z*-**2** is observed in the ^{1}H NMR spectrum (see Supplementary Figure 23 spectrum 3), which indicates increased *Z*-**2**·**10** complex formation. The proton 11 signal of residual *E*-**1** is also shifted a little further downfield. This latter behavior can be explaind with the higher binding constant of *E*-**1**, resulting in saturation of the remaining minority species *E*-**1** with **10** before residual **10** can be bound by the majority component tweezers *Z*-**2**. The signals of the open tweezers *Z*-**1** and *E*-**2** are not shifted at all upon additon of guest **10**, which shows that they do not possesss any affinity.

Irradiations to the pss in the mixture were reapeated twice to show full reversibility of the guest relocation.

Supplementary References

1 Friebolin, H. *Basic One- and Two-Dimensional NMR Spectroscopy*. 5 edn, (Wiley-VCH, 2010).

2 www.supramolecular.org.

3 Percec, V. *et al.* Self-Assembly of Dendronized Triphenylenes into Helical Pyramidal Columns and Chiral Spheres. *J. Am. Chem. Soc.* **131**, 7662-7677, (2009).

4 Connors, K. A. (eds Jerry L. Atwood *et al.*) Ch. 205 - 241, 205-241 (Pergamon, 1996).

5 Thordarson, P. Determining association constants from titration experiments in supramolecular chemistry. *Chem. Soc. Rev.* **40**, 1305-1323, (2011).

6 Kavallieratos, K., de Gala, S. R., Austin, A. J. & Crabtree, R. H. A Readily Available Non-preorganized Neutral Acyclic Halide Receptor with an Unusual Nonplanar Binding Conformation. *J. Am. Chem. Soc.* **119**, 2325-2326, (1997).

7 Schrödinger Release 2017-2: MacroModel, S., LLC, New York, NY, 2017.

8 Gaussian 16 Rev. A.03 (Wallingford, CT, 2016).

9 Gaussian 09 Rev. A.02 (Wallingford, CT, 2016).

10 Grimme, S. Supramolecular binding thermodynamics by dispersion-corrected density functional theory. *Chemistry* **18**, 9955-9964, (2012).
