# Supplementary material for: Simultaneous complementary photoswitching of hemithioindigo tweezers for dynamic guest relocalization
Source: Nat Commun. 2018 Apr 13;9:1456. doi: 10.1038/s41467-018-03912-7 (PMC5899155; doi:10.1038/s41467-018-03912-7)
Supplement: Supplementary file 2 — Description of Additional Supplementary Files [file 41467_2018_3912_MOESM2_ESM.pdf]

Description of Additional Supplementary Files

File Name: Supplementary Data 1
Description: Geometries of all calculated structures.

File Name: Supplementary Movie 1
Description: Simultaneous complementary photoswitching of two molecular tweezers for dynamic relocation of a guest molecule
