# Supplementary material for: Simultaneous complementary photoswitching of hemithioindigo tweezers for dynamic guest relocalization
Source: Nat Commun. 2018 Apr 13;9:1456. doi: 10.1038/s41467-018-03912-7 (PMC5899155; doi:10.1038/s41467-018-03912-7)
Supplement: Supplementary file 3 — Supplementary Data 1 [file 41467_2018_3912_MOESM3_ESM.pdf]

Supplementary Data 1

xyz coordinates of the optimized structures.

Z-**1** (MeO groups pointing to front) E = -2747.24288069				*E*-**1** (MeO groups pointing to front) E = -2747.24075124			
C	0.6192	-1.8902	-1.1799	C	-5.8160	2.0926	0.9460
O	2.7848	0.5948	0.1350	O	-2.9643	0.5542	-0.5060
C	0.4283	-3.2074	-1.5863	C	-6.4116	3.2391	1.4646
C	3.0099	-2.2424	-0.8803	C	-3.6678	3.2183	0.7144
C	2.8116	-3.5620	-1.2958	C	-4.2721	4.3645	1.2356
C	1.5423	-4.0372	-1.6382	C	-5.6225	4.3755	1.5990
C	1.8805	-1.4012	-0.8245	C	-4.4718	2.0691	0.5633
C	1.8475	0.0140	-0.3879	C	-4.0479	0.7603	0.0136
C	0.4830	0.6090	-0.5924	C	-5.1570	-0.2523	0.1102
C	0.1106	1.9146	-0.4547	C	-5.1152	-1.5725	-0.2326
C	1.0477	3.1456	-0.3069	C	-6.3540	-2.5037	-0.3319
C	-3.6745	3.8383	-0.3533	C	-2.0836	-4.4012	-1.1804
C	-2.4725	4.5293	-0.4888	C	-3.4370	-4.6208	-1.4195
C	-1.2797	3.8197	-0.5296	C	-4.3553	-3.6346	-1.0830
C	0.1140	4.3444	-0.6729	C	-5.8444	-3.6691	-1.2300
C	-2.4915	1.7354	-0.2870	C	-2.5709	-2.2240	-0.2558
C	-3.6967	2.4397	-0.2442	C	-1.6394	-3.2104	-0.5831
C	1.4889	3.2822	1.1689	C	-6.6766	-3.0448	1.0811
C	-1.2734	2.4173	-0.4426	C	-3.9355	-2.4129	-0.5266
C	2.2663	3.1675	-1.2460	C	-7.6175	-1.9013	-0.9713
H	-4.6114	4.3839	-0.3202	H	-1.3573	-5.1585	-1.4550
H	0.2914	4.6564	-1.7086	H	-6.1286	-3.4869	-2.2733
H	0.3097	5.2151	-0.0406	H	-6.2848	-4.6278	-0.9404
H	2.0900	4.1889	1.2892	H	-7.5257	-3.7333	1.0324
H	0.6218	3.3597	1.8311	H	-5.8229	-3.5832	1.4999
H	2.0909	2.4264	1.4691	H	-6.9357	-2.2339	1.7654
H	3.0271	2.4498	-0.9540	H	-8.1664	-1.2371	-0.3054
H	1.9650	2.9676	-2.2785	H	-7.3789	-1.3492	-1.8837
H	2.7138	4.1663	-1.2183	H	-8.2954	-2.7177	-1.2388
H	3.6664	-4.2263	-1.3500	H	-3.6746	5.2602	1.3609
H	1.4254	-5.0686	-1.9521	H	-6.0602	5.2842	1.9974
S	-0.6699	-0.6884	-1.0551	S	-6.6504	0.5557	0.6992
H	-2.4710	5.6121	-0.5579	H	-3.7696	-5.5539	-1.8622
H	-2.5242	0.6619	-0.1647	H	-2.2224	-1.3092	0.1930
C	-4.9955	1.7116	-0.0731	C	-0.1833	-2.9851	-0.3128
C	-5.7027	1.2582	-1.2031	C	0.5389	-2.0514	-1.0838
C	-5.5108	1.4815	1.2169	C	0.4668	-3.6928	0.7177
C	-6.9119	0.5855	-1.0241	C	1.8912	-1.8420	-0.8093
C	-6.7217	0.8022	1.3549	C	1.8199	-3.4560	0.9595
C	-7.4418	0.3448	0.2471	C	2.5545	-2.5318	0.2098
H	-7.4408	0.2184	-1.8968	H	2.4294	-1.1014	-1.3901
H	-7.1249	0.6476	2.3496	H	2.3151	-4.0188	1.7430
C	-8.7312	-0.3754	0.4158	C	3.9931	-2.2862	0.4936
C	-8.8975	-1.2922	1.4586	C	4.4759	-2.3402	1.8052
C	-9.7910	-0.1433	-0.4667	C	4.8842	-1.9981	-0.5466
C	-10.1084	-1.9659	1.6144	C	5.8258	-2.1167	2.0705
H	-8.0915	-1.5198	2.1434	H	3.8160	-2.5278	2.6415
C	-10.9992	-0.8208	-0.3064	C	6.2302	-1.7670	-0.2735
H	-9.7100	0.5795	-1.2677	H	4.5561	-1.9749	-1.5771
C	-11.1735	-1.7394	0.7350	C	6.7217	-1.8266	1.0349
H	-12.1090	-2.2620	0.8574	H	7.7653	-1.6496	1.2422

C	-5.1702	1.4787	-2.6007
H	-5.0258	2.5419	-2.8130
H	-4.1992	0.9956	-2.7422
H	-5.8595	1.0760	-3.3449
C	-4.7790	1.9614	2.4497
H	-3.7808	1.5201	2.5211
H	-4.6426	3.0466	2.4410
H	-5.3302	1.6976	3.3539
O	-11.9705	-0.5149	-1.2162
O	-10.1627	-2.8446	2.6582
C	-11.3572	-3.5791	2.8741
H	-11.6003	-4.2166	2.0162
H	-11.1666	-4.2071	3.7430
H	-12.2052	-2.9182	3.0877
C	-13.2333	-1.1527	-1.1104
H	-13.8362	-0.7548	-1.9253
H	-13.1483	-2.2396	-1.2245
H	-13.7228	-0.9245	-0.1566
C	4.3990	-1.7916	-0.5429
C	4.8668	-1.8692	0.7807
C	5.2525	-1.3450	-1.5679
C	6.1852	-1.5094	1.0558
C	3.9607	-2.3109	1.9047
C	6.5647	-0.9919	-1.2523
C	4.7684	-1.2181	-2.9945
C	7.0553	-1.0686	0.0542
H	6.5482	-1.5945	2.0741
H	3.1731	-1.5705	2.0743
H	3.4700	-3.2631	1.6866
H	4.5213	-2.4228	2.8345
H	7.2116	-0.6245	-2.0412
H	4.4214	-2.1755	-3.3933
H	3.9294	-0.5200	-3.0705
H	5.5674	-0.8530	-3.6420
C	8.4585	-0.6927	0.3688
C	9.4942	-1.0272	-0.5098
C	8.7582	-0.0007	1.5466
C	10.8093	-0.6729	-0.2125
H	9.3053	-1.5867	-1.4164
C	10.0757	0.3495	1.8391
H	7.9807	0.3019	2.2354
C	11.1173	0.0189	0.9645
O	11.7444	-1.0546	-1.1327
O	10.2580	1.0299	3.0095
H	12.1355	0.2915	1.1928
C	13.1071	-0.7424	-0.8932
C	11.5688	1.4356	3.3683
H	13.4745	-1.2131	0.0261
H	13.6575	-1.1424	-1.7435
H	13.2706	0.3401	-0.8371
H	11.4687	1.9564	4.3195
H	12.2371	0.5765	3.4983
H	11.9975	2.1203	2.6273
H	-0.5553	-3.5744	-1.8544
C	-0.1180	-1.2675	-2.1978
H	-0.6860	-1.9214	-2.8652
H	-0.8177	-0.5221	-1.8103
H	0.6357	-0.7467	-2.7911
C	-0.2727	-4.6921	1.5799
H	-1.2017	-4.2709	1.9730
H	-0.5458	-5.5925	1.0214
H	0.3453	-5.0017	2.4248
O	7.0150	-1.4829	-1.3620
O	6.1878	-2.1912	3.3856
C	7.5419	-1.9601	3.7372
H	7.8644	-0.9490	3.4624
H	7.5905	-2.0678	4.8198
H	8.2116	-2.6937	3.2732
C	8.4170	-1.3662	-1.1789
H	8.8353	-1.2003	-2.1708
H	8.6746	-0.5172	-0.5347
H	8.8446	-2.2826	-0.7572
C	-2.2077	3.2330	0.3792
C	-1.7799	3.4791	-0.9372
C	-1.2653	2.9963	1.3966
C	-0.4156	3.4545	-1.2215
C	-2.7720	3.7220	-2.0491
C	0.0917	2.9843	1.0735
C	-1.6935	2.7099	2.8182
C	0.5381	3.1926	-0.2337
H	-0.0872	3.6449	-2.2376
H	-3.3207	2.8039	-2.2807
H	-3.5093	4.4837	-1.7822
H	-2.2634	4.0455	-2.9591
H	0.8147	2.7601	1.8500
H	-2.2606	3.5402	3.2485
H	-2.3359	1.8259	2.8724
H	-0.8242	2.5310	3.4530
C	1.9823	3.0847	-0.5669
C	2.9534	3.6605	0.2572
C	2.3802	2.3620	-1.6953
C	4.3061	3.5063	-0.0446
H	2.6806	4.2439	1.1268
C	3.7345	2.1987	-1.9831
H	1.6534	1.8805	-2.3360
C	4.7138	2.7711	-1.1636
O	5.1747	4.1160	0.8170
O	4.0138	1.4406	-3.0843
H	5.7608	2.6355	-1.3834
C	6.5674	3.9903	0.5844
C	5.3726	1.1949	-3.4231
H	6.8574	4.4204	-0.3814
H	7.0539	4.5480	1.3835
H	6.8910	2.9437	0.6273
H	5.3446	0.5955	-4.3321
H	5.9093	2.1286	-3.6278
H	5.8890	0.6305	-2.6399
H	-7.4559	3.2468	1.7536

Z-**1** (MeO groups pointing back) E = -2747.24366795				*E*-**1** (MeO groups pointing back) E = -2747.24097001			
C	0.6693	-1.9201	-1.1347	C	5.4680	1.8244	-1.5392
O	2.7780	0.6240	0.1596	O	2.7149	0.5794	0.3340
C	0.5070	-3.2458	-1.5257	C	6.0004	2.8290	-2.3425
C	3.0656	-2.2197	-0.8230	C	3.3259	2.9741	-1.3849
C	2.8960	-3.5479	-1.2231	C	3.8656	3.9765	-2.1945
C	1.6378	-4.0532	-1.5639	C	5.1803	3.9037	-2.6656
C	1.9191	-1.4010	-0.7811	C	4.1534	1.8784	-1.0653
C	1.8555	0.0182	-0.3615	C	3.7804	0.7018	-0.2467
C	0.4805	0.5830	-0.5799	C	4.9144	-0.2850	-0.1773
C	0.0821	1.8829	-0.4632	C	4.9241	-1.4920	0.4581
C	0.9939	3.1345	-0.3320	C	6.1886	-2.3339	0.7708
C	-3.7404	3.7324	-0.4020	C	1.9756	-4.1262	2.0060
C	-2.5521	4.4449	-0.5472	C	3.3274	-4.2221	2.3264
C	-1.3454	3.7584	-0.5735	C	4.2211	-3.3096	1.7803
C	0.0382	4.3081	-0.7223	C	5.7096	-3.2500	1.9383
C	-2.5161	1.6547	-0.2981	C	2.4138	-2.2183	0.5942
C	-3.7351	2.3356	-0.2691	C	1.5086	-3.1400	1.1211
C	1.4250	3.3043	1.1433	C	6.5249	-3.2039	-0.4632
C	-1.3116	2.3582	-0.4630	C	3.7716	-2.2764	0.9397
C	2.2164	3.1649	-1.2657	C	7.4330	-1.5521	1.2243
H	-4.6879	4.2599	-0.3796	H	1.2695	-4.8282	2.4359
H	0.2128	4.6046	-1.7629	H	5.9752	-2.7945	2.8997
H	0.2146	5.1937	-0.1054	H	6.1869	-4.2337	1.9054
H	2.0077	4.2246	1.2511	H	7.3933	-3.8346	-0.2502
H	0.5535	3.3760	1.8003	H	5.6866	-3.8531	-0.7272
H	2.0419	2.4653	1.4602	H	6.7607	-2.5841	-1.3313
H	2.9900	2.4676	-0.9580	H	7.9499	-1.0506	0.4072
H	1.9244	2.9415	-2.2960	H	7.1803	-0.8026	1.9784
H	2.6437	4.1727	-1.2530	H	8.1438	-2.2541	1.6710
H	3.7642	-4.1953	-1.2664	H	3.2475	4.8275	-2.4561
H	1.5431	-5.0905	-1.8657	H	5.5675	4.7006	-3.2911
S	-0.6444	-0.7435	-1.0289	S	6.3549	0.3927	-1.0073
H	-2.5721	5.5261	-0.6345	H	3.6770	-5.0071	2.9887
H	-2.5273	0.5830	-0.1577	H	2.0519	-1.4589	-0.0789
C	-5.0191	1.5847	-0.0865	C	0.0635	-3.0651	0.7364
C	-5.7159	1.0970	-1.2087	C	-0.7301	-1.9878	1.1815
C	-5.5311	1.3666	1.2069	C	-0.5024	-4.0523	-0.0953
C	-6.9109	0.4020	-1.0187	C	-2.0653	-1.9162	0.7822
C	-6.7279	0.6647	1.3559	C	-1.8425	-3.9457	-0.4677
C	-7.4375	0.1721	0.2562	C	-2.6466	-2.8821	-0.0454
H	-7.4295	0.0056	-1.8848	H	-2.6573	-1.0659	1.1018
H	-7.1308	0.5222	2.3527	H	-2.2736	-4.7219	-1.0905
C	-8.7112	-0.5732	0.4370	C	-4.0678	-2.7770	-0.4692
C	-8.8331	-1.5113	1.4698	C	-4.4448	-3.1616	-1.7631
C	-9.7895	-0.3377	-0.4252	C	-5.0333	-2.2888	0.4204
C	-10.0341	-2.2099	1.6333	C	-5.7828	-3.0588	-2.1561
H	-7.9837	-1.7075	2.1078	H	-3.6881	-3.5017	-2.4548
C	-10.9850	-1.0424	-0.2483	C	-6.3670	-2.1856	0.0116
H	-9.6940	0.4115	-1.1977	H	-4.7399	-2.0217	1.4249
C	-11.1086	-1.9768	0.7776	C	-6.7439	-2.5724	-1.2729
H	-12.0352	-2.5194	0.9092	H	-7.7768	-2.4910	-1.5837
C	-5.1861	1.3034	-2.6095	C	-0.1687	-0.9068	2.0783
H	-5.0648	2.3655	-2.8412	H	0.3454	-1.3354	2.9432
H	-4.2044	0.8396	-2.7408	H	0.5567	-0.2768	1.5575
H	-5.8647	0.8717	-3.3473	H	-0.9710	-0.2618	2.4415
C	-4.8116	1.8847	2.4315	C	0.3132	-5.2178	-0.6111

H	-3.8043	1.4662	2.5122	H	1.2624	-4.8846	-1.0385
H	-4.6981	2.9721	2.4025	H	0.5568	-5.9310	0.1820
H	-5.3586	1.6267	3.3399	H	-0.2371	-5.7589	-1.3832
O	-12.0939	-0.8777	-1.0260	O	-7.3775	-1.7265	0.8044
O	-10.2486	-3.1496	2.5989	O	-6.2461	-3.3997	-3.3950
C	-9.1996	-3.4402	3.5108	C	-5.3260	-3.9095	-4.3476
H	-8.9134	-2.5560	4.0915	H	-4.8532	-4.8342	-3.9978
H	-9.5948	-4.1990	4.1843	H	-5.9115	-4.1221	-5.2409
H	-8.3171	-3.8380	2.9974	H	-4.5490	-3.1764	-4.5922
C	-12.0436	0.0568	-2.0938	C	-7.0593	-1.2498	2.1045
H	-13.0207	0.0122	-2.5724	H	-8.0004	-0.9070	2.5318
H	-11.8661	1.0754	-1.7308	H	-6.6461	-2.0445	2.7359
H	-11.2713	-0.2072	-2.8250	H	-6.3543	-0.4124	2.0642
C	4.4435	-1.7351	-0.4865	C	1.9168	3.1058	-0.8917
C	4.9074	-1.7852	0.8398	C	1.6613	3.7664	0.3230
C	5.2908	-1.2812	-1.5135	C	0.8514	2.5959	-1.6546
C	6.2153	-1.3894	1.1159	C	0.3432	3.9043	0.7555
C	4.0078	-2.2362	1.9653	C	2.7875	4.3030	1.1751
C	6.5923	-0.8913	-1.1967	C	-0.4533	2.7496	-1.1860
C	4.8098	-1.1838	-2.9434	C	1.0991	1.8547	-2.9478
C	7.0790	-0.9381	0.1129	C	-0.7337	3.3977	0.0211
H	6.5771	-1.4555	2.1361	H	0.1506	4.4362	1.6807
H	3.2026	-1.5126	2.1245	H	3.4117	3.4876	1.5536
H	3.5400	-3.2017	1.7553	H	3.4426	4.9748	0.6141
H	4.5674	-2.3260	2.8981	H	2.3962	4.8509	2.0341
H	7.2326	-0.5143	-1.9867	H	-1.2678	2.3206	-1.7591
H	4.4893	-2.1545	-3.3321	H	1.6720	2.4567	-3.6585
H	3.9533	-0.5088	-3.0312	H	1.6689	0.9367	-2.7750
H	5.6016	-0.8054	-3.5923	H	0.1561	1.5768	-3.4216
C	8.4693	-0.5184	0.4301	C	-2.1294	3.5340	0.5144
C	9.5235	-0.8836	-0.4169	C	-3.1689	3.8185	-0.3800
C	8.7258	0.2442	1.5765	C	-2.4074	3.3717	1.8784
C	10.8287	-0.4842	-0.1109	C	-4.4797	3.9379	0.0937
H	9.3171	-1.4983	-1.2810	H	-2.9396	3.9718	-1.4245
C	10.0359	0.6377	1.8687	C	-3.7226	3.4954	2.3380
H	7.8997	0.5482	2.2029	H	-1.6032	3.1199	2.5541
C	11.0865	0.2747	1.0286	C	-4.7582	3.7774	1.4497
O	11.9229	-0.7976	-0.8647	O	-5.5565	4.2224	-0.6937
O	10.3865	1.3894	2.9525	O	-4.0958	3.3446	3.6431
H	12.0978	0.5814	1.2598	H	-5.7735	3.8736	1.8104
C	11.7384	-1.5675	-2.0430	C	-5.3576	4.3674	-2.0924
C	9.3685	1.7975	3.8545	C	-3.0904	3.0869	4.6119
H	11.0866	-1.0571	-2.7610	H	-4.9522	3.4524	-2.5381
H	12.7296	-1.6843	-2.4786	H	-6.3428	4.5650	-2.5120
H	11.3245	-2.5570	-1.8182	H	-4.6932	5.2084	-2.3206
H	9.8736	2.3689	4.6318	H	-3.6084	3.0287	5.5681
H	8.6254	2.4352	3.3630	H	-2.5782	2.1371	4.4213
H	8.8637	0.9376	4.3089	H	-2.3510	3.8946	4.6505
H	-0.4678	-3.6361	-1.7929	H	7.0205	2.7765	-2.7039

E-**1** (MeO groups pointing to front, GD3BJ) E = -2747.52099365				*E*-**1** (MeO groups pointing back, GD3BJ) E = -2747.52363534			
C	-5.8966	2.1803	0.6115	C	5.380773	1.675792	-1.780821
O	-3.2853	0.6501	-1.1945	O	2.967429	0.989833	0.712747
C	-6.3862	3.3291	1.2242	C	5.775879	2.464047	-2.856925
C	-3.6399	3.1038	0.5309	C	3.143112	2.647430	-1.807584
C	-4.1302	4.2476	1.1609	C	3.536359	3.420877	-2.898858
C	-5.4873	4.3619	1.4809	C	4.838256	3.335985	-3.404079
C	-4.5567	2.0875	0.2305	C	4.093459	1.779971	-1.247814
C	-4.2472	0.8303	-0.4713	C	3.876234	0.891705	-0.091317
C	-5.3375	-0.1682	-0.2329	C	4.986260	-0.111038	0.000992
C	-5.2156	-1.5132	-0.3229	C	4.933262	-1.284184	0.674857
C	-6.3655	-2.5327	-0.2375	C	6.116193	-2.215471	0.987927
C	-1.9330	-4.1720	-0.6339	C	1.768421	-3.647519	2.190855
C	-3.2513	-4.5655	-0.8409	C	3.101312	-3.853125	2.539501
C	-4.2660	-3.6219	-0.7319	C	4.078646	-3.016295	2.012570
C	-5.7469	-3.8089	-0.8838	C	5.570691	-3.073432	2.172970
C	-2.6400	-1.9112	-0.1834	C	2.386381	-1.780962	0.797216
C	-1.6164	-2.8468	-0.2892	C	1.402792	-2.625304	1.299572
C	-6.6668	-2.8039	1.2514	C	6.364760	-3.125021	-0.233488
C	-3.9660	-2.2838	-0.4339	C	3.722280	-1.953819	1.168890
C	-7.6434	-2.1293	-0.9818	C	7.406409	-1.497309	1.397151
H	-1.1307	-4.8935	-0.7364	H	0.997843	-4.293579	2.595730
H	-6.0186	-3.8576	-1.9444	H	5.872532	-2.623076	3.125307
H	-6.1164	-4.7231	-0.4118	H	5.967688	-4.091645	2.153706
H	-7.4621	-3.5489	1.3431	H	7.183864	-3.818533	-0.023655
H	-5.7786	-3.1823	1.7627	H	5.471495	-3.707253	-0.470766
H	-6.9897	-1.8915	1.7563	H	6.631090	-2.534134	-1.112083
H	-8.1978	-1.3414	-0.4718	H	7.896794	-1.003819	0.557646
H	-7.4172	-1.7875	-1.9943	H	7.210907	-0.746657	2.166250
H	-8.3068	-2.9960	-1.0535	H	8.113549	-2.226796	1.802109
H	-3.4425	5.0428	1.4228	H	2.818072	4.088186	-3.359787
H	-5.8438	5.2633	1.9663	H	5.120745	3.952392	-4.249925
S	-6.8269	0.7035	0.2746	S	6.386591	0.457257	-0.968734
H	-3.4821	-5.5968	-1.0846	H	3.371870	-4.666881	3.203351
H	-2.3825	-0.8996	0.0794	H	2.092790	-1.001756	0.113866
C	-0.1978	-2.4416	-0.0674	C	-0.005074	-2.438453	0.842812
C	0.4368	-1.5742	-0.9785	C	-0.738073	-1.307790	1.249186
C	0.5133	-2.9423	1.0387	C	-0.573847	-3.365254	-0.049177
C	1.7905	-1.2886	-0.8084	C	-2.038134	-1.131699	0.771040
C	1.8583	-2.6102	1.1917	C	-1.867430	-3.149593	-0.517137
C	2.5219	-1.8078	0.2603	C	-2.619060	-2.039932	-0.118232
H	2.2790	-0.6274	-1.5118	H	-2.592927	-0.251378	1.072192
H	2.4107	-3.0193	2.0302	H	-2.311335	-3.876007	-1.188126
C	3.9771	-1.5568	0.3818	C	-4.000607	-1.860352	-0.627918
C	4.5569	-1.3080	1.6272	C	-4.290165	-2.166648	-1.964542
C	4.7878	-1.6110	-0.7555	C	-5.016691	-1.421674	0.225915
C	5.9339	-1.1408	1.7354	C	-5.603371	-2.059689	-2.429592
H	3.9538	-1.2173	2.5199	H	-3.488302	-2.471415	-2.619957
C	6.1625	-1.4413	-0.6384	C	-6.326511	-1.319423	-0.254148
H	4.3685	-1.8143	-1.7312	H	-4.779713	-1.190225	1.253090
C	6.7570	-1.2130	0.6059	C	-6.622009	-1.646463	-1.575707
H	7.8246	-1.0932	0.6943	H	-7.637343	-1.566681	-1.939445
C	-0.3083	-0.9478	-2.1317	C	-0.136133	-0.279362	2.174825
H	-0.9360	-1.6757	-2.6505	H	0.385234	-0.753280	3.009771
H	-0.9687	-0.1460	-1.7917	H	0.595452	0.345887	1.655557
H	0.3951	-0.5207	-2.8481	H	-0.913256	0.375667	2.568379
C	-0.1540	-3.8301	2.0629	C	0.198968	-4.573458	-0.522205

H	-1.1347	-3.4415	2.3470	H	1.194748	-4.293857	-0.875260
H	-0.3140	-4.8432	1.6823	H	0.342958	-5.303267	0.279355
H	0.4606	-3.9070	2.9616	H	-0.328583	-5.072544	-1.336773
O	6.8722	-1.5026	-1.8097	O	-7.384151	-0.905502	0.492952
O	6.4061	-0.8813	2.9904	O	-5.984141	-2.329683	-3.712245
C	7.8085	-0.8399	3.1892	C	-4.993158	-2.775930	-4.624053
H	8.2732	-0.0149	2.6371	H	-4.533453	-3.713016	-4.290963
H	7.9540	-0.6786	4.2559	H	-5.510504	-2.943436	-5.567132
H	8.2835	-1.7829	2.8959	H	-4.210854	-2.022399	-4.769200
C	8.2862	-1.4222	-1.7462	C	-7.142229	-0.524503	1.842096
H	8.6384	-1.5312	-2.7705	H	-8.103733	-0.194323	2.231276
H	8.6195	-0.4551	-1.3514	H	-6.782059	-1.372022	2.435549
H	8.7055	-2.2265	-1.1321	H	-6.425248	0.296518	1.892270
C	-2.1838	2.8940	0.2836	C	1.757743	2.688387	-1.255626
C	-1.5541	3.4705	-0.8282	C	1.411854	3.673853	-0.319909
C	-1.4642	2.0576	1.1551	C	0.842108	1.680453	-1.599756
C	-0.1971	3.2319	-1.0349	C	0.149893	3.633247	0.265896
C	-2.3431	4.2914	-1.8160	C	2.411809	4.719740	0.103102
C	-0.1106	1.8359	0.9150	C	-0.413314	1.670055	-0.995446
C	-2.1370	1.3746	2.3212	C	1.221211	0.579336	-2.558999
C	0.5451	2.4259	-0.1681	C	-0.772814	2.632347	-0.048528
H	0.2982	3.6986	-1.8787	H	-0.122504	4.398254	0.983961
H	-3.1175	3.6759	-2.2836	H	3.269065	4.249375	0.594410
H	-2.8477	5.1351	-1.3387	H	2.800670	5.283677	-0.748182
H	-1.6965	4.6808	-2.6037	H	1.963924	5.425025	0.804725
H	0.4422	1.1724	1.5696	H	-1.101761	0.864533	-1.222186
H	-2.7297	2.0763	2.9132	H	1.627488	0.978396	-3.491852
H	-2.8212	0.5882	1.9871	H	1.991386	-0.071857	-2.133600
H	-1.3962	0.9109	2.9739	H	0.356650	-0.042626	-2.794200
C	1.9969	2.2187	-0.3732	C	-2.083107	2.571989	0.639236
C	2.8608	2.2286	0.7234	C	-3.242093	2.249800	-0.072422
C	2.5158	2.0296	-1.6556	C	-2.146438	2.813544	2.016571
C	4.2311	2.0702	0.5347	C	-4.467429	2.183953	0.597816
H	2.4946	2.4041	1.7255	H	-3.178347	2.076811	-1.135813
C	3.8849	1.8364	-1.8325	C	-3.374810	2.730992	2.676258
H	1.8704	1.9891	-2.5228	H	-1.234809	3.024386	2.554505
C	4.7594	1.8652	-0.7422	C	-4.534420	2.421712	1.969527
O	4.9925	2.1328	1.6658	O	-5.651616	1.896642	-0.005881
O	4.2841	1.6027	-3.1157	O	-3.537748	2.930845	4.014994
H	5.8147	1.6967	-0.8783	H	-5.484580	2.386503	2.484367
C	6.3956	2.2710	1.5232	C	-5.651739	1.681407	-1.413768
C	5.6664	1.3865	-3.3666	C	-2.389302	3.245518	4.788666
H	6.6473	3.1659	0.9424	H	-5.010276	0.839810	-1.685919
H	6.7882	2.3681	2.5341	H	-6.679633	1.441351	-1.673205
H	6.8395	1.3916	1.0482	H	-5.333332	2.584052	-1.945917
H	5.7508	1.2485	-4.4435	H	-2.743987	3.365810	5.810708
H	6.2650	2.2555	-3.0692	H	-1.648608	2.439259	4.754111
H	6.0318	0.4904	-2.8578	H	-1.923669	4.178825	4.453857
H	-7.4272	3.4140	1.5106	H	6.775722	2.393946	-3.267277

Z-**2** (MeO groups pointing to front)
E = -2747.24767134

C	-4.1800	2.0515	0.7389
O	-7.1769	0.1758	1.1039
C	-3.5223	3.2746	0.9204
C	-6.2705	2.9522	1.5698
C	-5.6418	4.1749	1.7603
C	-4.2877	4.3260	1.4360
C	-5.5301	1.8910	1.0494
C	-6.0514	0.5353	0.7908
C	-4.9941	-0.3253	0.1653
C	-5.1229	-1.5970	-0.3148
C	-6.4488	-2.3597	-0.5876
C	-2.3059	-4.5429	-1.5020
C	-3.6684	-4.6402	-1.7736
C	-4.5185	-3.6224	-1.3621
C	-6.0009	-3.5191	-1.5353
C	-2.6438	-2.4185	-0.4068
C	-1.7816	-3.4409	-0.8086
C	-6.9796	-2.9614	0.7340
C	-4.0179	-2.4942	-0.6907
C	-7.5430	-1.5417	-1.2956
H	-1.6338	-5.3336	-1.8179
H	-6.2405	-3.2683	-2.5750
H	-6.5225	-4.4525	-1.3052
H	-7.8757	-3.5567	0.5321
H	-6.2336	-3.6164	1.1931
H	-7.2407	-2.1719	1.4368
H	-7.9934	-0.8017	-0.6408
H	-7.1406	-1.0375	-2.1790
H	-8.3304	-2.2240	-1.6320
H	-7.3152	2.7958	1.8121
H	-6.1937	5.0176	2.1599
H	-3.8063	5.2866	1.5862
S	-3.4308	0.5723	0.1237
H	-4.0597	-5.5066	-2.2963
H	-2.2307	-1.5927	0.1553
C	-2.0780	3.4560	0.5739
C	-1.0855	3.2345	1.5478
C	-1.7148	3.8550	-0.7271
C	0.2540	3.4208	1.2054
C	-0.3645	4.0281	-1.0305
C	0.6384	3.8181	-0.0790
H	1.0172	3.2252	1.9503
H	-0.0896	4.3578	-2.0262
C	-0.3190	-3.3771	-0.4881
C	0.5592	-2.6634	-1.3259
C	0.1795	-4.0396	0.6503
C	1.9194	-2.6311	-1.0159
C	1.5452	-3.9808	0.9285
C	2.4364	-3.2842	0.1069
H	2.5875	-2.0617	-1.6525
H	1.9263	-4.5115	1.7940
C	3.8895	-3.2434	0.4177
C	4.3265	-3.1316	1.7414
C	4.8348	-3.3217	-0.6101
C	5.6901	-3.1008	2.0305
H	3.6259	-3.0387	2.5605
C	6.1969	-3.2893	-0.3148
H	4.5356	-3.4371	-1.6434

E-**2** (MeO groups pointing to front)
E = -2747.24570435

C	2.0329	-1.0819	-1.0475
O	-1.3788	-0.2917	-1.6574
C	3.0394	-2.0559	-1.0668
C	0.3663	-2.6623	-1.8180
C	1.3448	-3.6463	-1.8455
C	2.6597	-3.3394	-1.4732
C	0.7199	-1.3755	-1.4124
C	-0.2063	-0.2302	-1.3210
C	0.5118	0.9900	-0.8161
C	0.0017	2.2353	-0.5838
C	0.8516	3.5041	-0.2975
C	-3.8739	3.9389	-0.1980
C	-2.7162	4.6983	-0.3424
C	-1.4939	4.0533	-0.4766
C	-0.1356	4.6656	-0.6144
C	-2.5860	1.8981	-0.3130
C	-3.8183	2.5364	-0.1672
C	1.2079	3.5299	1.2080
C	-1.4128	2.6487	-0.4907
C	2.1210	3.6874	-1.1483
H	-4.8353	4.4323	-0.1029
H	0.0206	5.0174	-1.6411
H	0.0207	5.5235	0.0461
H	1.7765	4.4353	1.4405
H	0.3061	3.5238	1.8252
H	1.8165	2.6669	1.4864
H	2.9509	3.0576	-0.8326
H	1.9244	3.4885	-2.2048
H	2.4534	4.7261	-1.0572
H	-0.6612	-2.8610	-2.0996
H	1.0976	-4.6551	-2.1547
H	3.4172	-4.1158	-1.4985
S	2.2609	0.6101	-0.5940
H	-2.7697	5.7820	-0.3447
H	-2.5492	0.8217	-0.3016
C	4.4499	-1.7436	-0.6769
C	5.3732	-1.3246	-1.6542
C	4.8539	-1.8744	0.6659
C	6.6859	-1.0471	-1.2724
C	6.1750	-1.5852	1.0077
C	7.1102	-1.1706	0.0543
H	7.3878	-0.7023	-2.0235
H	6.4884	-1.7098	2.0382
C	-5.0686	1.7301	0.0065
C	-5.6260	1.0517	-1.0958
C	-5.6878	1.6438	1.2688
C	-6.7963	0.3129	-0.9178
C	-6.8528	0.8887	1.4071
C	-7.4287	0.2164	0.3252
H	-7.2108	-0.2210	-1.7658
H	-7.3333	0.8404	2.3781
C	-8.6730	-0.5794	0.4924
C	-8.8723	-1.3471	1.6444
C	-9.6578	-0.5692	-0.5006
C	-10.0405	-2.0923	1.7983
H	-8.1208	-1.4038	2.4207
C	-10.8234	-1.3174	-0.3415
H	-9.5521	0.0338	-1.3927

10
E = -1337.80083705

C	-3.0626	-1.2518	-0.0307
C	-3.2956	0.1109	-0.0471
C	-2.2667	1.0456	-0.0138
C	-0.9594	0.5748	0.0302
C	-0.6691	-0.8189	0.0609
C	-1.7493	-1.7052	0.0438
H	-3.8856	-1.9503	-0.0733
H	-2.5199	2.0949	-0.0326
C	1.3816	0.3138	0.0188
C	2.7642	0.4673	-0.0320
C	3.5391	-0.6862	-0.0689
C	2.9907	-1.9615	-0.0559
C	1.6071	-2.1143	0.0027
C	0.7980	-0.9812	0.0412
H	3.2584	1.4267	-0.0495
H	3.6483	-2.8190	-0.0868
H	1.1863	-3.1062	0.0355
C	0.3134	1.3256	0.0366
C	0.4628	2.6853	0.0676
C	1.7350	3.3331	0.0877
C	-0.6326	3.6007	0.0914
N	2.7436	3.8963	0.1047
N	-1.4917	4.3730	0.1115
N	-4.6998	0.5900	-0.1075
O	-4.8736	1.7969	-0.1446
O	-5.5772	-0.2589	-0.1162
N	-1.5958	-3.1763	0.1197
O	-0.7202	-3.6125	0.8535
O	-2.3791	-3.8482	-0.5279
N	5.0168	-0.5440	-0.1280
O	5.6756	-1.5723	-0.1559
O	5.4698	0.5893	-0.1455

C	6.6421	-3.1794	1.0075	C	-11.0302	-2.0877	0.8087
H	7.6964	-3.1558	1.2336	H	-11.9328	-2.6655	0.9297
C	2.0714	4.0105	-0.4242	C	8.5139	-0.8696	0.4399
C	2.5578	3.6037	-1.6704	C	8.7906	-0.2115	1.6423
C	2.9462	4.6001	0.4936	C	9.5713	-1.2421	-0.3959
C	3.9026	3.7844	-1.9914	C	10.1085	0.0684	2.0018
H	1.9178	3.1166	-2.3939	H	7.9977	0.1178	2.3007
C	4.2897	4.7789	0.1664	C	10.8871	-0.9592	-0.0313
H	2.6004	4.9523	1.4564	H	9.3984	-1.7780	-1.3198
C	4.7846	4.3739	-1.0785	C	11.1723	-0.3013	1.1707
H	5.8244	4.5124	-1.3290	H	12.1909	-0.0836	1.4507
C	-1.4482	2.7920	2.9468	C	4.9627	-1.1654	-3.1002
H	-2.0647	3.5379	3.4570	H	4.6338	-2.1155	-3.5312
H	-2.0205	1.8601	2.9367	H	4.1304	-0.4640	-3.2059
H	-0.5506	2.6298	3.5457	H	5.7956	-0.7941	-3.6996
C	-2.7603	4.1058	-1.7893	C	3.8855	-2.3307	1.7328
H	-3.3686	3.2149	-1.9698	H	3.0199	-1.6655	1.7984
H	-3.4469	4.9058	-1.4980	H	3.5003	-3.3331	1.5246
H	-2.2915	4.3905	-2.7328	H	4.3696	-2.3519	2.7106
C	0.0546	-1.9281	-2.5466	C	-4.9803	1.1052	-2.4622
H	-0.5089	-2.5899	-3.2102	H	-4.8093	2.1366	-2.7831
H	-0.6166	-1.1088	-2.2734	H	-4.0076	0.6058	-2.4692
H	0.8857	-1.5050	-3.1133	H	-5.6153	0.6192	-3.2053
C	-0.7360	-4.8130	1.5724	C	-5.1102	2.3430	2.4794
H	-1.5436	-4.1832	1.9559	H	-4.0499	2.1095	2.6076
H	-1.2105	-5.6548	1.0597	H	-5.1856	3.4313	2.3968
H	-0.1809	-5.2086	2.4246	H	-5.6378	2.0411	3.3860
O	4.2763	3.3440	-3.2286	O	10.2682	0.7212	3.1902
O	7.0349	-3.3813	-1.3900	O	-11.7232	-1.2305	-1.3657
O	6.0087	-2.9822	3.3532	O	-10.1309	-2.8129	2.9554
O	5.0558	5.3726	1.1285	O	11.8439	-1.3734	-0.9129
C	7.3762	-2.9300	3.7263	C	-11.2825	-3.6108	3.1765
H	7.8837	-2.0656	3.2825	H	-11.3934	-4.3830	2.4064
H	7.3847	-2.8308	4.8106	H	-11.1313	-4.0896	4.1428
H	7.9075	-3.8468	3.4455	H	-12.1943	-3.0036	3.2156
C	6.4313	5.5985	0.8656	C	13.2084	-1.1351	-0.6053
H	6.8296	6.0777	1.7586	H	13.7768	-1.5503	-1.4361
H	6.9692	4.6594	0.6916	H	13.4231	-0.0634	-0.5233
H	6.5746	6.2640	0.0064	H	13.5074	-1.6380	0.3216
C	8.4356	-3.3812	-1.1677	C	-12.9392	-1.9546	-1.2716
H	8.8928	-3.4717	-2.1519	H	-13.4886	-1.7327	-2.1852
H	8.7714	-2.4481	-0.7003	H	-12.7633	-3.0349	-1.2119
H	8.7468	-4.2299	-0.5476	H	-13.5342	-1.6354	-0.4080
C	5.6349	3.4669	-3.6180	C	11.5794	1.0529	3.6191
H	6.2985	2.9044	-2.9512	H	12.0777	1.7259	2.9118
H	5.6957	3.0462	-4.6205	H	11.4593	1.5632	4.5734
H	5.9537	4.5153	-3.6489	H	12.1954	0.1581	3.7653

Z-**2** (MeO groups pointing back)				*E*-**2** (MeO groups pointing back)			
E = -2747.24767134				E = -2747.24637157			
C	-4.0412	2.0224	0.6323	C	2.0683	-1.1082	-0.9662
O	-7.0152	0.1260	1.0693	O	-1.3579	-0.4120	-1.6079
C	-3.4003	3.2604	0.7671	C	3.0950	-2.0611	-0.9498
C	-6.1445	2.9268	1.4274	C	0.4370	-2.7491	-1.6837
C	-5.5325	4.1643	1.5713	C	1.4359	-3.7129	-1.6751
C	-4.1804	4.3204	1.2419	C	2.7432	-3.3657	-1.3115
C	-5.3893	1.8567	0.9486	C	0.7627	-1.4417	-1.3228
C	-5.8936	0.4855	0.7435	C	-0.1875	-0.3136	-1.2730
C	-4.8247	-0.3873	0.1555	C	0.5040	0.9386	-0.8114
C	-4.9365	-1.6815	-0.2650	C	-0.0317	2.1812	-0.6262
C	-6.2529	-2.4734	-0.4995	C	0.7919	3.4765	-0.3850
C	-2.0855	-4.6553	-1.2924	C	-3.9420	3.8195	-0.3135
C	-3.4463	-4.7810	-1.5608	C	-2.7991	4.5962	-0.4816
C	-4.3074	-3.7508	-1.2071	C	-1.5637	3.9716	-0.5889
C	-5.7902	-3.6727	-1.3882	C	-0.2176	4.6057	-0.7456
C	-2.4471	-2.4765	-0.3174	C	-2.6130	1.8021	-0.3509
C	-1.5741	-3.5108	-0.6613	C	-3.8584	2.4204	-0.2324
C	-6.7764	-3.0168	0.8501	C	1.1438	3.5645	1.1191
C	-3.8202	-2.5827	-0.5964	C	-1.4544	2.5693	-0.5518
C	-7.3568	-1.7052	-1.2471	C	2.0593	3.6544	-1.2400
H	-1.4051	-5.4564	-1.5607	H	-4.9132	4.2967	-0.2394
H	-6.0295	-3.4758	-2.4395	H	-0.0657	4.9234	-1.7840
H	-6.3022	-4.5993	-1.1139	H	-0.0805	5.4899	-0.1163
H	-7.6647	-3.6324	0.6773	H	1.6932	4.4892	1.3193
H	-6.0224	-3.6395	1.3401	H	0.2408	3.5627	1.7346
H	-7.0480	-2.1980	1.5141	H	1.7688	2.7247	1.4300
H	-7.8167	-0.9394	-0.6299	H	2.9010	3.0534	-0.9004
H	-6.9602	-1.2407	-2.1546	H	1.8692	3.4131	-2.2889
H	-8.1354	-2.4131	-1.5491	H	2.3704	4.7022	-1.1865
H	-7.1872	2.7656	1.6751	H	-0.5853	-2.9792	-1.9603
H	-6.0958	5.0143	1.9381	H	1.2105	-4.7367	-1.9493
H	-3.7118	5.2924	1.3556	H	3.5166	-4.1266	-1.3085
S	-3.2737	0.5294	0.0740	S	2.2599	0.6030	-0.5718
H	-3.8280	-5.6791	-2.0348	H	-2.8742	5.6778	-0.5232
H	-2.0430	-1.6158	0.1970	H	-2.5547	0.7278	-0.3010
C	-1.9586	3.4531	0.4155	C	4.4975	-1.7050	-0.5687
C	-0.9625	3.2697	1.3937	C	5.4132	-1.2972	-1.5579
C	-1.6015	3.8330	-0.8928	C	4.9018	-1.7818	0.7782
C	0.3734	3.4764	1.0485	C	6.7180	-0.9757	-1.1833
C	-0.2547	4.0294	-1.1979	C	6.2151	-1.4504	1.1121
C	0.7523	3.8597	-0.2421	C	7.1429	-1.0446	0.1474
H	1.1397	3.3079	1.7971	H	7.4124	-0.6358	-1.9438
H	0.0137	4.3504	-2.1983	H	6.5300	-1.5368	2.1462
C	-0.1126	-3.4150	-0.3433	C	-5.0928	1.5952	-0.0352
C	0.7710	-2.8027	-1.2524	C	-5.6305	0.8664	-1.1150
C	0.3801	-3.9479	0.8635	C	-5.7167	1.5408	1.2265
C	2.1302	-2.7392	-0.9429	C	-6.7860	0.1098	-0.9158
C	1.7453	-3.8618	1.1384	C	-6.8668	0.7670	1.3863
C	2.6423	-3.2632	0.2481	C	-7.4235	0.0441	0.3269
H	2.8026	-2.2440	-1.6349	H	-7.1831	-0.4654	-1.7451
H	2.1230	-4.2973	2.0571	H	-7.3536	0.7460	2.3554
C	4.0943	-3.1888	0.5586	C	-8.6525	-0.7708	0.5166
C	4.5177	-2.8725	1.8556	C	-8.8134	-1.5378	1.6775
C	5.0391	-3.4377	-0.4448	C	-9.6509	-0.7721	-0.4657
C	5.8855	-2.8063	2.1411	C	-9.9722	-2.3023	1.8483
H	3.7795	-2.6501	2.6123	H	-8.0218	-1.5545	2.4125
C	6.4036	-3.3700	-0.1438	C	-10.8051	-1.5402	-0.2804
H	4.6999	-3.7119	-1.4331	H	-9.5293	-0.1522	-1.3421

C	6.8276	-3.0551	1.1453	C	-10.9673	-2.3044	0.8732
H	7.8841	-3.0023	1.3718	H	-11.8616	-2.8973	1.0109
C	2.1808	4.0827	-0.5884	C	8.5378	-0.6958	0.5253
C	2.6810	3.6382	-1.8186	C	8.7833	0.0497	1.6853
C	3.0255	4.7411	0.3140	C	9.6063	-1.1123	-0.2786
C	4.0253	3.8550	-2.1390	C	10.0984	0.3755	2.0341
H	2.0279	3.1021	-2.4917	H	7.9487	0.3927	2.2793
C	4.3674	4.9520	-0.0207	C	10.9163	-0.7811	0.0841
H	2.6203	5.1081	1.2458	H	9.4062	-1.7141	-1.1531
C	4.8677	4.5106	-1.2438	C	11.1633	-0.0386	1.2368
H	5.9066	4.6750	-1.4966	H	12.1782	0.2154	1.5119
C	-1.3182	2.8482	2.8009	C	5.0022	-1.1953	-3.0089
H	-1.9529	3.5901	3.2941	H	4.6964	-2.1665	-3.4088
H	-1.8690	1.9035	2.8092	H	4.1538	-0.5176	-3.1387
H	-0.4185	2.7190	3.4049	H	5.8271	-0.8240	-3.6193
C	-2.6507	4.0415	-1.9605	C	3.9421	-2.2263	1.8579
H	-3.2467	3.1379	-2.1170	H	3.0612	-1.5794	1.9012
H	-3.3484	4.8390	-1.6897	H	3.5805	-3.2432	1.6811
H	-2.1865	4.3086	-2.9114	H	4.4246	-2.2057	2.8365
C	0.2706	-2.2050	-2.5477	C	-4.9782	0.8838	-2.4792
H	-0.2711	-2.9411	-3.1482	H	-4.8267	1.9063	-2.8365
H	-0.4201	-1.3764	-2.3667	H	-3.9953	0.4050	-2.4630
H	1.1020	-1.8259	-3.1446	H	-5.5984	0.3577	-3.2075
C	-0.5410	-4.6151	1.8596	C	-5.1607	2.2955	2.4135
H	-1.3268	-3.9337	2.1979	H	-4.0967	2.0888	2.5556
H	-1.0444	-5.4838	1.4256	H	-5.2577	3.3783	2.2904
H	0.0157	-4.9515	2.7359	H	-5.6875	2.0169	3.3280
O	4.6097	3.4530	-3.3046	O	10.4409	1.1051	3.1346
O	7.4001	-3.6063	-1.0456	O	-11.8373	-1.6001	-1.1714
O	6.3984	-2.4964	3.3670	O	-10.2176	-3.0898	2.9359
O	5.2662	5.5911	0.7822	O	12.0234	-1.1479	-0.6235
C	5.4991	-2.2254	4.4317	C	-9.2477	-3.1401	3.9714
H	4.8600	-3.0885	4.6499	H	-9.0839	-2.1535	4.4194
H	6.1226	-2.0128	5.2988	H	-9.6540	-3.8131	4.7250
H	4.8704	-1.3540	4.2170	H	-8.2922	-3.5372	3.6109
C	4.8328	6.0613	2.0498	C	11.8517	-1.9067	-1.8114
H	5.7075	6.5171	2.5112	H	12.8537	-2.0729	-2.2038
H	4.0428	6.8144	1.9527	H	11.3792	-2.8741	-1.6075
H	4.4765	5.2415	2.6836	H	11.2586	-1.3629	-2.5553
C	7.0460	-3.9231	-2.3834	C	-11.7414	-0.8515	-2.3740
H	7.9870	-4.0551	-2.9152	H	-12.6579	-1.0577	-2.9248
H	6.4672	-4.8520	-2.4389	H	-11.6739	0.2241	-2.1759
H	6.4770	-3.1127	-2.8526	H	-10.8806	-1.1636	-2.9760
C	3.8134	2.7798	-4.2679	C	9.4094	1.5595	3.9983
H	2.9846	3.4066	-4.6157	H	8.8447	0.7239	4.4266
H	4.4775	2.5672	-5.1043	H	9.9110	2.1011	4.7988
H	3.4150	1.8377	-3.8748	H	8.7197	2.2366	3.4819

Z-**2** (MeO groups pointing to front, GD3BJ)				*Z*-**2** (MeO groups pointing back, GD3BJ)			
E = -2747.52490194				E = -2747.52783874			
C	-4.348415	2.086467	0.601482	C	-4.017352	-2.238545	-0.414696
O	-7.272056	0.116982	1.009806	O	-7.102785	-0.533810	-0.836331
C	-3.711715	3.324467	0.720520	C	-3.273093	-3.415583	-0.528394
C	-6.475153	2.995228	1.274249	C	-6.060522	-3.335575	-1.059377
C	-5.870804	4.241532	1.388036	C	-5.350315	-4.526295	-1.160086
C	-4.503340	4.401221	1.115374	C	-3.971178	-4.562724	-0.901480
C	-5.701435	1.906138	0.870820	C	-5.381575	-2.176950	-0.679642
C	-6.152445	0.507318	0.714702	C	-5.947005	-0.815237	-0.557738
C	-5.023785	-0.348565	0.218120	C	-4.881467	0.151983	-0.128237
C	-5.041592	-1.663100	-0.139103	C	-4.978934	1.492097	0.102437
C	-6.279190	-2.558065	-0.370339	C	-6.265811	2.333239	0.246270
C	-1.904113	-4.307030	-1.200542	C	-1.992963	4.411699	0.844703
C	-3.248829	-4.598706	-1.419548	C	-3.354321	4.652949	1.028346
C	-4.219019	-3.684272	-1.029805	C	-4.273178	3.646655	0.755705
C	-5.706587	-3.770379	-1.176020	C	-5.764197	3.665199	0.896958
C	-2.498908	-2.218228	-0.161385	C	-2.473131	2.187127	0.063406
C	-1.515066	-3.122255	-0.557730	C	-1.539850	3.179306	0.352783
C	-6.810569	-3.045787	0.992432	C	-6.838859	2.626510	-1.154866
C	-3.851123	-2.470642	-0.428257	C	-3.837784	2.392902	0.298999
C	-7.396402	-1.898892	-1.192064	C	-7.329553	1.701152	1.155625
H	-1.141440	-5.003228	-1.529128	H	-1.271072	5.183665	1.085068
H	-5.988215	-3.688293	-2.230941	H	-6.043672	3.696575	1.955136
H	-6.109192	-4.719758	-0.812368	H	-6.218707	4.539089	0.422406
H	-7.647010	-3.733695	0.837043	H	-7.714479	3.276232	-1.065618
H	-6.029693	-3.575578	1.544836	H	-6.097207	3.135444	-1.776605
H	-7.158997	-2.202553	1.586620	H	-7.141160	1.701948	-1.643808
H	-7.903964	-1.118079	-0.634224	H	-7.776219	0.821177	0.702758
H	-6.993587	-1.473610	-2.115342	H	-6.894237	1.424746	2.119814
H	-8.132145	-2.661235	-1.465889	H	-8.120635	2.434072	1.341148
H	-7.525873	2.845829	1.492324	H	-7.121330	-3.281716	-1.273045
H	-6.455033	5.100196	1.696122	H	-5.859864	-5.436637	-1.451832
H	-4.044103	5.378141	1.217943	H	-3.429247	-5.496492	-1.002250
S	-3.509969	0.617667	0.130335	S	-3.306068	-0.698906	0.029244
H	-3.532353	-5.528805	-1.899858	H	-3.690124	5.617809	1.392274
H	-2.184915	-1.336477	0.374146	H	-2.106046	1.268794	-0.364107
C	-2.250540	3.365260	0.426552	C	-1.808984	-3.318678	-0.259247
C	-1.337834	2.991497	1.428607	C	-0.961956	-2.798905	-1.255222
C	-1.809406	3.579934	-0.890231	C	-1.314175	-3.557716	1.033705
C	0.001478	2.812885	1.090646	C	0.358917	-2.498143	-0.931884
C	-0.460720	3.399285	-1.189936	C	0.018258	-3.261587	1.315185
C	0.458740	2.993391	-0.217999	C	0.869912	-2.709099	0.352603
H	0.693659	2.474051	1.852134	H	0.990692	-2.045944	-1.686932
H	-0.120521	3.571802	-2.204228	H	0.401061	-3.468721	2.307561
C	-0.081708	-2.802406	-0.298434	C	-0.095733	2.869539	0.141340
C	0.508773	-1.672533	-0.898783	C	0.519718	1.869311	0.919787
C	0.676897	-3.607334	0.575071	C	0.631392	3.509495	-0.880402
C	1.844290	-1.375584	-0.625991	C	1.850101	1.536387	0.671464
C	2.006627	-3.277172	0.823956	C	1.955656	3.138275	-1.109966
C	2.611819	-2.165971	0.229631	C	2.583474	2.154943	-0.341506
H	2.281246	-0.491040	-1.071958	H	2.312877	0.744613	1.247715
H	2.588935	-3.911027	1.482855	H	2.516350	3.635765	-1.893737
C	4.033583	-1.840892	0.494427	C	3.989107	1.756296	-0.589010
C	4.585374	-2.053569	1.759836	C	4.425229	1.490174	-1.891994
C	4.839261	-1.326906	-0.526918	C	4.864350	1.613630	0.488991
C	5.927329	-1.767494	1.999009	C	5.741162	1.069723	-2.100265
H	3.984192	-2.419327	2.580844	H	3.724544	1.577933	-2.709206

C	6.177050	-1.043031	-0.278366	C	6.178672	1.198645	0.261808
H	4.441589	-1.163727	-1.518481	H	4.509070	1.820893	1.486966
C	6.742870	-1.261311	0.980902	C	6.618219	0.927458	-1.030076
H	7.781988	-1.042128	1.165281	H	7.629708	0.585176	-1.197842
C	1.870544	2.708252	-0.567403	C	2.266440	-2.338931	0.685514
C	2.183408	2.133538	-1.802683	C	2.579553	-1.867638	1.967262
C	2.895953	2.971590	0.342680	C	3.271455	-2.440941	-0.281091
C	3.502055	1.805599	-2.108693	C	3.897306	-1.519451	2.271144
H	1.413713	1.893950	-2.522401	H	1.789671	-1.743543	2.692073
C	4.213815	2.647208	0.025683	C	4.584678	-2.085136	0.039320
H	2.698082	3.441296	1.296300	H	3.029961	-2.828215	-1.259159
C	4.533988	2.056817	-1.200125	C	4.903109	-1.638200	1.318431
H	5.543967	1.754952	-1.423729	H	5.919407	-1.351647	1.547881
C	-1.807303	2.724780	2.836888	C	-1.483452	-2.516821	-2.642178
H	-2.351814	3.579569	3.245609	H	-1.979335	-3.392387	-3.067803
H	-2.487701	1.868669	2.867755	H	-2.218787	-1.707089	-2.628388
H	-0.962687	2.509461	3.492651	H	-0.671153	-2.223069	-3.308450
C	-2.788187	3.949989	-1.976373	C	-2.221797	-4.086696	2.116009
H	-3.526868	3.155962	-2.121720	H	-3.054250	-3.400143	2.296375
H	-3.342825	4.857925	-1.726636	H	-2.659399	-5.049478	1.840247
H	-2.273509	4.112442	-2.924363	H	-1.677020	-4.215344	3.052350
C	-0.251889	-0.760130	-1.833000	C	-0.222461	1.123166	2.004158
H	-1.028391	-1.292333	-2.382700	H	-0.926558	1.765950	2.534565
H	-0.737832	0.055062	-1.288151	H	-0.793928	0.285381	1.593924
H	0.433577	-0.306355	-2.549940	H	0.483886	0.709735	2.726313
C	0.074704	-4.808361	1.266335	C	-0.005883	4.568681	-1.748078
H	-0.921241	-4.586954	1.655828	H	-0.985275	4.249009	-2.111915
H	-0.031341	-5.659671	0.587602	H	-0.160966	5.502828	-1.200577
H	0.706708	-5.126613	2.097109	H	0.626301	4.791168	-2.609216
O	3.691649	1.190349	-3.312472	O	4.288697	-1.004949	3.478227
O	6.884404	-0.519974	-1.332714	O	7.097617	1.009045	1.250329
O	6.366834	-2.005207	3.267711	O	6.252883	0.740190	-3.326005
O	5.137671	2.946730	0.983088	O	5.623452	-2.149739	-0.834703
C	7.714769	-1.700570	3.584398	C	5.426503	0.923012	-4.463543
H	7.929834	-0.635308	3.441660	H	5.109424	1.966484	-4.566205
H	7.838412	-1.953486	4.635771	H	6.034186	0.643638	-5.322594
H	8.413818	-2.295894	2.985894	H	4.538593	0.281070	-4.425368
C	6.491557	2.599061	0.742299	C	5.330840	-2.346941	-2.210221
H	7.043078	2.930666	1.620734	H	6.265765	-2.180643	-2.740394
H	6.605883	1.517724	0.627600	H	4.971432	-3.364095	-2.401839
H	6.885681	3.108368	-0.144785	H	4.588221	-1.622119	-2.557834
C	8.291328	-0.386122	-1.206120	C	6.736344	1.370473	2.580309
H	8.646538	-0.031098	-2.172129	H	7.625008	1.190709	3.183075
H	8.561343	0.344872	-0.435722	H	6.466060	2.430313	2.639762
H	8.763460	-1.346553	-0.974927	H	5.913499	0.755087	2.954278
C	5.025606	1.025301	-3.784318	C	3.307616	-0.855072	4.493050
H	5.602249	0.350493	-3.147213	H	2.855331	-1.816791	4.757058
H	4.932690	0.597006	-4.781002	H	3.832285	-0.454137	5.358512
H	5.537683	1.991979	-3.850492	H	2.520597	-0.155469	4.190734

E-**1**·**10** complex A				*E*-**1**·**10** complex B			
E = -4085.49401675				E = -4085.49036644			
C	6.2956	-2.8719	0.3347	C	-6.0565	-2.6720	-1.5608
O	3.6462	-0.5176	0.2453	O	-3.8967	-0.7515	0.4857
C	6.6992	-4.1833	0.5648	C	-6.2910	-3.8385	-2.2806
C	3.9640	-3.4301	0.7378	C	-3.7428	-3.2997	-1.1346
C	4.3748	-4.7428	0.9766	C	-3.9888	-4.4695	-1.8586
C	5.7204	-5.1120	0.8948	C	-5.2404	-4.7371	-2.4169
C	4.9557	-2.4839	0.4261	C	-4.8110	-2.3945	-0.9828
C	4.7467	-1.0490	0.1726	C	-4.8021	-1.1181	-0.2437
C	6.0362	-0.3561	-0.1466	C	-6.0964	-0.3826	-0.4152
C	6.2703	0.9597	-0.4393	C	-6.4051	0.8574	0.0525
C	7.6894	1.5261	-0.7302	C	-7.8263	1.4585	0.1208
C	3.9964	4.5110	-1.0293	C	-4.2007	4.1957	1.4543
C	5.3864	4.4883	-0.9802	C	-5.5760	4.0653	1.6287
C	6.0389	3.2827	-0.7702	C	-6.2148	2.9266	1.1556
C	7.5070	3.0622	-0.6295	C	-7.6731	2.5879	1.1834
C	3.9233	2.1162	-0.7069	C	-4.1145	2.0709	0.3117
C	3.2609	3.3280	-0.8893	C	-3.4641	3.2090	0.7821
C	8.8181	1.1131	0.2300	C	-8.1541	2.0841	-1.2517
C	5.3247	2.0761	-0.6244	C	-5.4866	1.9047	0.5232
C	8.0491	1.1513	-2.1849	C	-8.9422	0.4996	0.5549
H	3.4698	5.4475	-1.1746	H	-3.6898	5.0805	1.8165
H	8.0979	3.5897	-1.3830	H	-7.9577	2.2074	2.1707
H	7.8440	3.4185	0.3502	H	-8.3195	3.4418	0.9646
H	9.6431	1.8209	0.1113	H	-9.1440	2.5480	-1.2238
H	8.4880	1.1502	1.2705	H	-7.4207	2.8493	-1.5152
H	9.2203	0.1223	0.0330	H	-8.1530	1.3257	-2.0373
H	8.0819	0.0690	-2.3192	H	-9.2513	-0.1837	-0.2344
H	7.3143	1.5604	-2.8821	H	-8.6392	-0.0931	1.4209
H	9.0313	1.5588	-2.4399	H	-9.8209	1.0876	0.8346
H	3.6247	-5.4865	1.2166	H	-3.1829	-5.1829	-1.9804
H	6.0031	-6.1411	1.0848	H	-5.3931	-5.6589	-2.9662
S	7.3798	-1.5520	-0.0783	S	-7.2718	-1.4239	-1.2768
H	5.9551	5.4047	-1.0931	H	-6.1416	4.8504	2.1181
H	3.3515	1.2133	-0.6069	H	-3.5540	1.3303	-0.2297
C	1.7695	3.3579	-0.8823	C	-1.9944	3.3515	0.5623
C	1.0416	3.0387	-2.0386	C	-1.0980	3.1541	1.6289
C	1.0967	3.6900	0.3071	C	-1.4952	3.6334	-0.7213
C	-0.3508	3.0454	-1.9855	C	0.2721	3.1852	1.3824
C	-0.2938	3.6804	0.3237	C	-0.1172	3.6832	-0.9210
C	-1.0440	3.3552	-0.8109	C	0.7956	3.4273	0.1070
H	-0.9003	2.8192	-2.8905	H	0.9358	2.9697	2.2089
H	-0.7969	3.8949	1.2573	H	0.2394	3.9034	-1.9177
C	-2.5241	3.3043	-0.7496	C	2.2569	3.3512	-0.1578
C	-3.2258	3.9895	0.2452	C	2.7485	3.2881	-1.4666
C	-3.2433	2.5213	-1.6619	C	3.1731	3.2809	0.8979
C	-4.6129	3.8831	0.3327	C	4.1165	3.1757	-1.7094
H	-2.7261	4.6191	0.9667	H	2.0930	3.2875	-2.3240
C	-4.6297	2.4248	-1.5766	C	4.5380	3.1599	0.6494
H	-2.7461	1.9468	-2.4292	H	2.8650	3.3328	1.9315
C	-5.3335	3.1021	-0.5786	C	5.0331	3.1159	-0.6559
H	-6.4034	3.0058	-0.4982	H	6.0893	3.0094	-0.8413
C	1.7489	2.6820	-3.3214	C	-1.5982	2.8663	3.0226
H	2.5258	3.4107	-3.5646	H	-2.0046	3.7674	3.4911
H	2.2308	1.7047	-3.2416	H	-2.3933	2.1200	3.0169
H	1.0438	2.6375	-4.1529	H	-0.7866	2.5042	3.6568
C	1.8583	4.0302	1.5641	C	-2.4210	3.8436	-1.8944
H	2.5951	3.2622	1.8095	H	-2.7831	2.8861	-2.2804
H	2.4102	4.9677	1.4528	H	-3.2960	4.4333	-1.6155

H	1.1799	4.1406	2.4111	H	-1.9006	4.3518	-2.7076
O	-5.2142	1.5866	-2.4788	O	5.3241	3.0484	1.7568
O	-5.1897	4.5686	1.3534	O	4.4735	3.0797	-3.0209
C	-6.5777	4.3843	1.5975	C	5.8574	3.0292	-3.3444
H	-6.8128	3.3309	1.7806	H	6.3562	2.1825	-2.8635
H	-6.7986	4.9629	2.4918	H	5.9065	2.9055	-4.4238
H	-7.1841	4.7562	0.7644	H	6.3639	3.9556	-3.0544
C	-6.6351	1.4531	-2.4681	C	6.7370	3.1579	1.6029
H	-6.8663	0.7292	-3.2456	H	7.1428	3.1535	2.6122
H	-6.9909	1.0713	-1.5076	H	7.1468	2.3065	1.0530
H	-7.1172	2.4101	-2.6924	H	7.0016	4.0947	1.1026
C	2.4951	-3.1546	0.7740	C	-2.3550	-3.1121	-0.6061
C	1.7423	-3.3535	-0.3946	C	-2.0514	-3.3667	0.7421
C	1.8526	-2.8488	1.9835	C	-1.3157	-2.8333	-1.5132
C	0.3516	-3.3105	-0.3177	C	-0.7162	-3.3885	1.1455
C	2.4237	-3.6023	-1.7163	C	-3.1380	-3.6112	1.7556
C	0.4600	-2.8245	2.0230	C	0.0034	-2.8445	-1.0653
C	2.6544	-2.5384	3.2208	C	-1.6047	-2.5194	-2.9619
C	-0.3187	-3.0752	0.8886	C	0.3401	-3.1421	0.2606
H	-0.2125	-3.4767	-1.2255	H	-0.5076	-3.6283	2.1798
H	1.6957	-3.7770	-2.5068	H	-3.5976	-2.6605	2.0378
H	3.0258	-2.7364	-2.0068	H	-3.9241	-4.2561	1.3575
H	3.0981	-4.4605	-1.6634	H	-2.7337	-4.0731	2.6573
H	-0.0195	-2.6284	2.9736	H	0.7805	-2.6074	-1.7802
H	2.0034	-2.3814	4.0816	H	-0.7133	-2.1260	-3.4544
H	3.3566	-3.3409	3.4602	H	-1.9225	-3.4095	-3.5111
H	3.2414	-1.6269	3.0763	H	-2.4003	-1.7784	-3.0556
C	-1.8019	-3.0831	0.9536	C	1.7571	-3.1821	0.7108
C	-2.4780	-2.4406	2.0000	C	2.0816	-3.1803	2.0715
C	-2.5559	-3.7261	-0.0311	C	2.8022	-3.1926	-0.2213
C	-3.8683	-2.4668	2.0663	C	3.4146	-3.2119	2.4832
H	-1.9497	-1.9070	2.7757	H	1.3281	-3.1249	2.8436
C	-3.9499	-3.7171	0.0205	C	4.1324	-3.2177	0.1945
H	-2.0910	-4.2564	-0.8494	H	2.6205	-3.1985	-1.2857
C	-4.6258	-3.0979	1.0751	C	4.4592	-3.2424	1.5518
O	-4.4210	-1.7905	3.1128	O	3.6076	-3.1812	3.8273
O	-4.5777	-4.3163	-1.0275	O	5.0544	-3.1775	-0.8067
H	-5.7029	-3.0787	1.1103	H	5.4881	-3.2539	1.8719
C	-5.7870	-2.0413	3.4412	C	4.9381	-3.1357	4.3255
C	-5.9967	-4.4503	-0.9862	C	6.4376	-3.2822	-0.4739
H	-5.9556	-3.1108	3.5999	H	5.4725	-2.2538	3.9588
H	-5.9679	-1.4988	4.3669	H	4.8450	-3.0712	5.4071
H	-6.4618	-1.6667	2.6676	H	5.4946	-4.0402	4.0576
H	-6.2662	-4.9772	-1.8988	H	6.9711	-3.2151	-1.4192
H	-6.3093	-5.0343	-0.1148	H	6.7585	-2.4624	0.1739
H	-6.4872	-3.4736	-0.9834	H	6.6511	-4.2421	0.0074
H	7.7412	-4.4691	0.4923	H	-7.2613	-4.0408	-2.7171
C	-2.4162	-1.5564	-2.9090	C	-0.9954	0.0462	0.6861
C	-3.7062	-1.4469	-2.4310	C	-0.3655	-0.2215	1.8866
C	-4.0044	-0.8206	-1.2277	C	1.0118	-0.1772	2.0362
C	-2.9563	-0.3024	-0.4888	C	1.7732	0.0583	0.9031
C	-1.6032	-0.4101	-0.9214	C	1.1848	0.2142	-0.3823
C	-1.3735	-1.0192	-2.1580	C	-0.2125	0.2822	-0.4399
H	-2.2170	-2.0525	-3.8459	H	-2.0714	-0.0063	0.6148
H	-5.0302	-0.7604	-0.9125	H	1.4453	-0.3796	3.0018
C	-1.5885	0.6202	1.1805	C	3.5008	-0.0064	-0.6739
C	-1.0514	1.1153	2.3605	C	4.6995	-0.1671	-1.3491
C	0.3320	1.0718	2.4970	C	4.6546	-0.2235	-2.7356
C	1.1723	0.5754	1.5127	C	3.4702	-0.1154	-3.4507
C	0.6338	0.1125	0.3178	C	2.2699	0.0645	-2.7706
C	-0.7436	0.1296	0.1451	C	2.2740	0.1345	-1.3797
H	-1.6515	1.5088	3.1656	H	5.6478	-0.2682	-0.8511

H	2.2380	0.5404	1.6694	H	3.4987	-0.1638	-4.5293
H	1.3011	-0.2793	-0.4278	H	1.3566	0.1700	-3.3286
C	-2.9879	0.4026	0.8046	C	3.2333	0.0186	0.7653
C	-4.1001	0.7810	1.5022	C	4.1444	0.0134	1.7837
C	-4.0525	1.5322	2.7097	C	5.5444	-0.0870	1.5630
C	-5.4223	0.4312	1.1129	C	3.7906	0.1489	3.1549
N	-4.0667	2.1457	3.6885	N	6.6850	-0.1983	1.4109
N	-6.5099	0.1362	0.8541	N	3.5421	0.2691	4.2768
N	-4.8078	-2.0053	-3.2279	N	-1.1801	-0.6235	3.0455
O	-5.9484	-1.8385	-2.8058	O	-0.6000	-1.2068	3.9544
O	-4.5259	-2.5948	-4.2586	O	-2.3741	-0.3733	3.0197
N	-0.0399	-1.1338	-2.7718	N	-0.9465	0.5904	-1.6760
O	0.1446	-2.0698	-3.5384	O	-2.1474	0.3598	-1.7019
O	0.7892	-0.2753	-2.5093	O	-0.3311	1.0965	-2.6047
N	0.9317	1.5786	3.7439	N	5.9165	-0.4048	-3.4683
O	2.1097	1.3180	3.9541	O	5.8761	-0.3750	-4.6906
O	0.2170	2.2356	4.4895	O	6.9396	-0.5633	-2.8090

E-**1**·**10** complex C				*E*-**1**·**10** complex D			
E = -4085.48791674				E = -4085.48481919			
C	6.3300	-2.7243	0.3856	C	6.6282	-2.3531	-0.1245
O	3.5965	-0.4630	0.2870	O	3.8696	-0.2489	-0.8702
C	6.7854	-4.0142	0.6401	C	7.1510	-3.6367	-0.0092
C	4.0302	-3.3555	0.8460	C	4.3997	-3.2111	-0.5769
C	4.4916	-4.6470	1.1062	C	4.9315	-4.4973	-0.4611
C	5.8478	-4.9700	1.0086	C	6.2840	-4.7075	-0.1857
C	4.9789	-2.3809	0.4919	C	5.2776	-2.1225	-0.4092
C	4.7125	-0.9608	0.2030	C	4.9486	-0.6842	-0.5050
C	5.9722	-0.2374	-0.1699	C	6.1419	0.1660	-0.1724
C	6.1588	1.0692	-0.5296	C	6.2131	1.5233	-0.0739
C	7.5551	1.6631	-0.8740	C	7.5368	2.3139	0.0877
C	3.7653	4.5030	-1.2995	C	3.4841	4.7504	0.3401
C	5.1553	4.5280	-1.2697	C	4.8450	4.9272	0.1114
C	5.8482	3.3597	-0.9915	C	5.6563	3.8125	-0.0469
C	7.3239	3.1954	-0.8570	C	7.1323	3.7694	-0.2763
C	3.7719	2.1333	-0.8150	C	3.7536	2.3512	0.2564
C	3.0701	3.3097	-1.0675	C	2.9325	3.4639	0.4209
C	8.7085	1.3415	0.0922	C	7.9512	2.2603	1.5739
C	5.1753	2.1429	-0.7586	C	5.1233	2.5117	0.0120
C	7.9083	1.2212	-2.3114	C	8.7004	1.8806	-0.8161
H	3.2061	5.4103	-1.4984	H	2.8387	5.6114	0.4724
H	7.8896	3.6986	-1.6456	H	7.3555	3.9661	-1.3311
H	7.6582	3.6170	0.0974	H	7.6852	4.5042	0.3146
H	9.5080	2.0681	-0.0769	H	8.8688	2.8357	1.7255
H	8.3893	1.4265	1.1333	H	7.1691	2.6836	2.2084
H	9.1413	0.3549	-0.0542	H	8.1330	1.2339	1.8967
H	7.9744	0.1351	-2.3889	H	9.2016	0.9774	-0.4743
H	7.1519	1.5694	-3.0186	H	8.3627	1.7167	-1.8420
H	8.8734	1.6454	-2.6017	H	9.4481	2.6788	-0.8274
H	3.7728	-5.4104	1.3784	H	4.2713	-5.3454	-0.5948
H	6.1711	-5.9834	1.2170	H	6.6621	-5.7203	-0.1076
S	7.3594	-1.3804	-0.0804	S	7.5803	-0.8816	0.0544
H	5.6927	5.4523	-1.4507	H	5.2681	5.9246	0.0673
H	3.2312	1.2219	-0.6404	H	3.3328	1.3664	0.3120
C	1.5795	3.3060	-1.0354	C	1.4723	3.2910	0.6786
C	0.8368	2.9169	-2.1604	C	0.5463	3.5531	-0.3498
C	0.9216	3.6939	0.1447	C	1.0167	2.8906	1.9485
C	-0.5543	2.9237	-2.0884	C	-0.8125	3.3946	-0.0928
C	-0.4680	3.6750	0.1829	C	-0.3528	2.7389	2.1637
C	-1.2352	3.2920	-0.9227	C	-1.2919	2.9807	1.1554
H	-1.1116	2.6505	-2.9753	H	-1.5054	3.5768	-0.9031
H	-0.9544	3.9296	1.1150	H	-0.6886	2.4543	3.1531
C	-2.7144	3.2404	-0.8382	C	-2.7445	2.7819	1.3806
C	-3.3972	3.9420	0.1646	C	-3.2007	1.9292	2.3929
C	-3.4445	2.4504	-1.7371	C	-3.6839	3.4023	0.5511
C	-4.7844	3.8301	0.2787	C	-4.5647	1.6988	2.5612
H	-2.8506	4.5797	0.8400	H	-2.5176	1.4003	3.0407
C	-4.8318	2.3568	-1.6216	C	-5.0456	3.1620	0.7179
H	-2.9280	1.8796	-2.4919	H	-3.3889	4.0907	-0.2273
C	-5.5057	3.0294	-0.6098	C	-5.5050	2.3061	1.7243
H	-6.5777	2.9340	-0.5106	H	-6.5583	2.1123	1.8423
C	1.5275	2.4845	-3.4287	C	1.0011	3.9816	-1.7215
H	2.3044	3.1938	-3.7231	H	1.4174	4.9928	-1.7026
H	2.0067	1.5113	-3.2971	H	1.7786	3.3179	-2.1032
H	0.8126	2.3937	-4.2481	H	0.1686	3.9696	-2.4262
C	1.7002	4.1025	1.3706	C	1.9863	2.6414	3.0760
H	2.4362	3.3454	1.6503	H	2.5619	1.7284	2.9064
H	2.2558	5.0282	1.1980	H	2.7003	3.4627	3.1706

H	1.0339	4.2649	2.2187	H	1.4560	2.5314	4.0233
O	-5.5990	1.5616	-2.4229	O	-5.8635	3.7869	-0.1710
O	-5.5241	4.4567	1.2270	O	-4.8948	0.8226	3.5510
C	-4.8625	5.3211	2.1473	C	-6.2629	0.4687	3.7124
H	-4.3680	6.1470	1.6254	H	-6.6627	0.0032	2.8055
H	-5.6463	5.7150	2.7907	H	-6.2869	-0.2491	4.5301
H	-4.1409	4.7716	2.7564	H	-6.8712	1.3391	3.9795
C	-5.1052	1.2323	-3.7153	C	-7.2729	3.6622	-0.0049
H	-5.9137	0.7006	-4.2124	H	-7.7148	4.2629	-0.7973
H	-4.8483	2.1362	-4.2766	H	-7.5935	2.6233	-0.1140
H	-4.2290	0.5790	-3.6662	H	-7.5903	4.0532	0.9676
C	2.5558	-3.1277	0.9130	C	2.9386	-3.0840	-0.8560
C	1.7802	-3.3756	-0.2300	C	2.4801	-2.7438	-2.1382
C	1.9378	-2.8097	2.1319	C	2.0186	-3.3983	0.1620
C	0.3912	-3.3626	-0.1186	C	1.1093	-2.7281	-2.3852
C	2.4355	-3.6422	-1.5615	C	3.4311	-2.3568	-3.2387
C	0.5470	-2.8187	2.2069	C	0.6560	-3.3533	-0.1210
C	2.7633	-2.4478	3.3391	C	2.4781	-3.7311	1.5599
C	-0.2539	-3.1112	1.0980	C	0.1722	-3.0175	-1.3907
H	-0.1915	-3.5573	-1.0088	H	0.7755	-2.4827	-3.3851
H	1.6919	-3.7809	-2.3447	H	3.6943	-1.3006	-3.1346
H	3.0720	-2.8021	-1.8526	H	4.3542	-2.9380	-3.2055
H	3.0735	-4.5292	-1.5218	H	2.9696	-2.4941	-4.2179
H	0.0868	-2.6157	3.1657	H	-0.0385	-3.5578	0.6832
H	2.1307	-2.2820	4.2119	H	1.6353	-3.7612	2.2516
H	3.4905	-3.2267	3.5817	H	2.9819	-4.7006	1.5982
H	3.3241	-1.5273	3.1536	H	3.1828	-2.9830	1.9276
C	-1.7340	-3.1203	1.1920	C	-1.2843	-2.9288	-1.6599
C	-2.3840	-2.4100	2.2092	C	-2.2056	-3.5874	-0.8407
C	-2.5002	-3.8096	0.2438	C	-1.7658	-2.1610	-2.7280
C	-3.7781	-2.3895	2.2692	C	-3.5733	-3.4914	-1.0886
H	-1.8039	-1.8452	2.9205	H	-1.8896	-4.2097	-0.0160
C	-3.8940	-3.7523	0.2937	C	-3.1333	-2.0705	-2.9744
H	-2.0085	-4.3881	-0.5218	H	-1.1011	-1.5993	-3.3681
C	-4.5412	-3.0426	1.3033	C	-4.0567	-2.7368	-2.1628
O	-4.4793	-1.7044	3.2151	O	-4.3739	-4.1686	-0.2208
O	-4.7057	-4.3352	-0.6324	O	-3.4896	-1.2594	-4.0130
H	-5.6209	-2.9987	1.3376	H	-5.1154	-2.6627	-2.3514
C	-3.7793	-1.2325	4.3628	C	-5.7677	-4.2431	-0.4966
C	-4.1328	-5.2698	-1.5429	C	-4.8578	-1.1987	-4.3915
H	-3.0402	-0.4698	4.1037	H	-5.9519	-4.6846	-1.4817
H	-4.5335	-0.7839	5.0050	H	-6.1849	-4.8908	0.2722
H	-3.2854	-2.0581	4.8845	H	-6.2399	-3.2591	-0.4315
H	-4.9618	-5.6434	-2.1392	H	-4.9057	-0.4929	-5.2172
H	-3.4085	-4.7924	-2.2090	H	-5.2218	-2.1797	-4.7138
H	-3.6544	-6.0950	-1.0063	H	-5.4865	-0.8296	-3.5743
H	7.8360	-4.2630	0.5555	H	8.2001	-3.7950	0.2083
C	-2.6585	-1.8690	-2.7647	C	-4.4081	1.0101	-1.8164
C	-3.9492	-1.7122	-2.2954	C	-3.1982	1.3772	-2.3638
C	-4.2480	-0.9686	-1.1605	C	-1.9891	1.0084	-1.7990
C	-3.1908	-0.4035	-0.4644	C	-2.0188	0.2400	-0.6459
C	-1.8408	-0.5508	-0.8919	C	-3.2373	-0.1890	-0.0437
C	-1.6150	-1.2572	-2.0774	C	-4.4278	0.2143	-0.6759
H	-2.4634	-2.4444	-3.6562	H	-5.3314	1.3294	-2.2703
H	-5.2779	-0.8577	-0.8647	H	-1.0713	1.3240	-2.2655
C	-1.8056	0.6401	1.1210	C	-1.4686	-0.8669	1.3437
C	-1.2499	1.2165	2.2544	C	-0.8408	-1.4773	2.4198
C	0.1337	1.1639	2.3877	C	-1.6377	-2.1587	3.3320
C	0.9644	0.5867	1.4392	C	-3.0158	-2.2411	3.2023
C	0.4097	0.0464	0.2849	C	-3.6430	-1.6311	2.1213
C	-0.9705	0.0658	0.1214	C	-2.8839	-0.9331	1.1862
H	-1.8352	1.6835	3.0300	H	0.2246	-1.4517	2.5738

H	2.0315	0.5507	1.5906	H	-3.5843	-2.7856	3.9417
H	1.0673	-0.4003	-0.4395	H	-4.7054	-1.7194	2.0130
C	-3.2112	0.3922	0.7767	C	-0.8847	-0.1780	0.1910
C	-4.3209	0.7862	1.4685	C	0.4449	-0.0067	-0.0772
C	-4.2439	1.6188	2.6202	C	1.4416	-0.2926	0.8976
C	-5.6449	0.3737	1.1442	C	0.9261	0.4475	-1.3368
N	-4.1947	2.2874	3.5609	N	2.2300	-0.4697	1.7217
N	-6.7245	0.0276	0.9249	N	1.2958	0.7717	-2.3813
N	-5.0508	-2.3418	-3.0440	N	-3.1967	2.1935	-3.5864
O	-6.1890	-2.0113	-2.7567	O	-2.1530	2.7591	-3.8816
O	-4.7469	-3.1502	-3.9146	O	-4.2385	2.2559	-4.2250
N	-0.2891	-1.3933	-2.7005	N	-5.7750	-0.1771	-0.2488
O	-0.0908	-2.3904	-3.3836	O	-6.7288	0.4346	-0.7133
O	0.5186	-0.4915	-2.5329	O	-5.8933	-1.1260	0.5186
N	0.7422	1.7572	3.5906	N	-0.9828	-2.8394	4.4665
O	1.9167	1.5000	3.8199	O	-1.7037	-3.3368	5.3201
O	0.0360	2.4781	4.2856	O	0.2412	-2.8680	4.4783

Z-**2**·**10** complex E				*Z*-**2**·**10** complex F			
E = -4085.49669399				E = -4085.49547824			
C	-4.5956	2.4307	-1.0036	C	4.6404	2.6306	-0.4732
O	-7.8968	1.2782	-1.6624	O	8.0161	1.6972	-1.0916
C	-3.6229	3.4139	-1.2012	C	3.6620	3.6269	-0.5080
C	-6.2571	3.6940	-2.2262	C	6.3653	4.1638	-1.1967
C	-5.3119	4.6921	-2.4269	C	5.4139	5.1757	-1.2309
C	-4.0140	4.5454	-1.9226	C	4.0827	4.9031	-0.8935
C	-5.8918	2.5584	-1.5066	C	5.9704	2.8845	-0.8118
C	-6.7596	1.3945	-1.2306	C	6.8431	1.6937	-0.7496
C	-6.0093	0.3662	-0.4408	C	6.0585	0.4985	-0.2983
C	-6.4341	-0.8544	-0.0107	C	6.4789	-0.7879	-0.1387
C	-7.8884	-1.3594	0.0993	C	7.9366	-1.2939	-0.0724
C	-4.3193	-4.2004	1.5037	C	4.3550	-4.4192	0.4028
C	-5.7017	-4.0607	1.6195	C	5.7317	-4.3088	0.5885
C	-6.3196	-2.9293	1.1021	C	6.3500	-3.0794	0.4006
C	-7.7713	-2.5607	1.0953	C	7.7952	-2.7205	0.5515
C	-4.1815	-2.1078	0.3146	C	4.2258	-2.0900	-0.2075
C	-3.5513	-3.2343	0.8392	C	3.5937	-3.3149	-0.0036
C	-8.8806	-0.3335	0.6624	C	8.4957	-1.4151	-1.5047
C	-5.5606	-1.9291	0.4748	C	5.6006	-1.9532	0.0241
C	-8.3490	-1.8756	-1.2791	C	8.8623	-0.4488	0.8136
H	-3.8274	-5.0771	1.9095	H	3.8620	-5.3724	0.5563
H	-8.4183	-3.3914	0.8021	H	8.0664	-2.6946	1.6122
H	-8.0848	-2.2466	2.0966	H	8.4621	-3.4392	0.0682
H	-9.8247	-0.8389	0.8877	H	9.5059	-1.8336	-1.4703
H	-8.5004	0.0991	1.5917	H	7.8707	-2.0785	-2.1087
H	-9.0792	0.4661	-0.0449	H	8.5418	-0.4368	-1.9799
H	-8.3702	-1.0603	-2.0005	H	9.0773	0.5174	0.3677
H	-7.6791	-2.6592	-1.6434	H	8.4148	-0.2957	1.7993
H	-9.3558	-2.2955	-1.1972	H	9.8068	-0.9837	0.9521
H	-7.2655	3.7686	-2.6153	H	7.4009	4.3370	-1.4635
H	-5.5717	5.5863	-2.9802	H	5.6956	6.1797	-1.5236
H	-3.2813	5.3269	-2.0913	H	3.3468	5.6989	-0.9280
S	-4.3353	0.9309	-0.1349	S	4.3475	0.9610	-0.0318
H	-6.2871	-4.8321	2.1074	H	6.3130	-5.1769	0.8789
H	-3.5945	-1.4067	-0.2574	H	3.6375	-1.2683	-0.5838
C	-2.2255	3.2716	-0.6926	C	2.2326	3.3623	-0.1639
C	-1.2021	2.8659	-1.5695	C	1.2836	3.1987	-1.1912
C	-1.9153	3.5921	0.6410	C	1.8207	3.3353	1.1797
C	0.1142	2.8354	-1.1145	C	-0.0646	3.0770	-0.8601
C	-0.5827	3.5666	1.0525	C	0.4601	3.2244	1.4699
C	0.4604	3.2058	0.1909	C	-0.5094	3.1202	0.4676
H	0.8825	2.5078	-1.8031	H	-0.7829	2.9477	-1.6604
H	-0.3633	3.8698	2.0676	H	0.1566	3.2719	2.5083
C	-2.0736	-3.3763	0.6774	C	2.1177	-3.4095	-0.2037
C	-1.5327	-3.7638	-0.5611	C	1.2631	-3.4743	0.9119
C	-1.2146	-3.0652	1.7466	C	1.5707	-3.3771	-1.4990
C	-0.1487	-3.8091	-0.7147	C	-0.1158	-3.4625	0.7161
C	0.1637	-3.0979	1.5455	C	0.1860	-3.3886	-1.6535
C	0.7280	-3.4470	0.3138	C	-0.6862	-3.4001	-0.5597
H	0.2440	-4.1157	-1.6745	H	-0.7492	-3.4577	1.5930
H	0.8008	-2.7975	2.3668	H	-0.2117	-3.3713	-2.6593
C	2.1953	-3.3751	0.0871	C	-2.1568	-3.2891	-0.7421
C	3.0806	-3.2556	1.1638	C	-2.7003	-2.8649	-1.9603
C	2.7205	-3.3678	-1.2104	C	-3.0287	-3.5524	0.3197
C	4.4517	-3.1415	0.9470	C	-4.0806	-2.7344	-2.1148
H	2.7429	-3.2649	2.1897	H	-2.0780	-2.5941	-2.7997
C	4.0954	-3.2651	-1.4201	C	-4.4054	-3.4067	0.1630
H	2.0863	-3.4039	-2.0830	H	-2.6760	-3.8936	1.2817

C	4.9819	-3.1574	-0.3456	C	-4.9536	-3.0100	-1.0590
H	6.0429	-3.0570	-0.5057	H	-6.0186	-2.8890	-1.1705
C	1.8764	3.2201	0.6435	C	-1.9559	3.0638	0.8000
C	2.9182	3.1984	-0.2821	C	-2.3707	2.6166	2.0685
C	2.1889	3.2468	2.0162	C	-2.9209	3.4434	-0.1290
C	4.2543	3.2296	0.1396	C	-3.7234	2.5545	2.3773
H	2.7372	3.1891	-1.3468	H	-1.6370	2.2867	2.7863
C	3.5191	3.2914	2.4261	C	-4.2837	3.3706	0.1889
H	1.4004	3.2098	2.7502	H	-2.6525	3.8234	-1.1042
C	4.5649	3.2936	1.4903	C	-4.6951	2.9251	1.4398
H	5.5792	3.3172	1.8591	H	-5.7338	2.8451	1.7230
C	-1.5193	2.4595	-2.9875	C	1.7159	3.1425	-2.6348
H	-1.8909	3.3051	-3.5719	H	2.4571	2.3547	-2.7867
H	-0.6296	2.0715	-3.4871	H	0.8654	2.9385	-3.2872
H	-2.2907	1.6871	-3.0126	H	2.1719	4.0828	-2.9541
C	-2.9959	3.9618	1.6253	C	2.8216	3.4398	2.3029
H	-3.7155	4.6577	1.1901	H	3.5312	4.2520	2.1328
H	-3.5493	3.0710	1.9368	H	2.3200	3.6110	3.2564
H	-2.5676	4.4160	2.5198	H	3.4011	2.5162	2.3908
C	-2.4221	-4.0912	-1.7350	C	1.8129	-3.5130	2.3170
H	-3.2912	-4.6771	-1.4310	H	2.1928	-4.5079	2.5668
H	-2.7944	-3.1760	-2.2048	H	2.6418	-2.8154	2.4440
H	-1.8700	-4.6504	-2.4917	H	1.0361	-3.2682	3.0439
C	-1.7635	-2.6596	3.0922	C	2.4511	-3.2978	-2.7219
H	-2.5764	-1.9391	2.9937	H	2.7772	-2.2684	-2.8976
H	-2.1647	-3.5228	3.6312	H	3.3476	-3.9103	-2.6137
H	-0.9813	-2.2186	3.7133	H	1.9070	-3.6272	-3.6083
O	5.1768	3.1619	-0.8570	O	-4.2089	2.0842	3.5605
O	4.4886	-3.2236	-2.7239	O	-5.1457	-3.6388	1.2810
O	5.2076	-2.9749	2.0672	O	-4.4947	-2.2765	-3.3288
O	3.9163	3.3059	3.7228	O	-5.1383	3.7427	-0.8021
C	6.6232	-3.1147	1.9628	C	-5.8924	-2.1978	-3.5809
H	7.0642	-2.3008	1.3815	H	-6.3985	-1.5484	-2.8608
H	6.9983	-3.0620	2.9826	H	-5.9921	-1.7740	-4.5777
H	6.8855	-4.0812	1.5218	H	-6.3534	-3.1905	-3.5543
C	2.9174	3.3349	4.7403	C	-6.5280	3.8368	-0.4924
H	3.4610	3.3678	5.6816	H	-7.0090	4.1761	-1.4072
H	2.2883	4.2258	4.6458	H	-6.9431	2.8656	-0.2112
H	2.2988	2.4346	4.7160	H	-6.7009	4.5611	0.3095
C	5.8810	-3.1919	-3.0104	C	-6.5635	-3.7314	1.1550
H	5.9615	-3.1166	-4.0924	H	-6.9262	-4.0215	2.1386
H	6.3691	-2.3259	-2.5529	H	-7.0047	-2.7683	0.8860
H	6.3753	-4.1058	-2.6652	H	-6.8381	-4.4939	0.4198
C	6.5593	3.2616	-0.5127	C	-3.3116	1.9778	4.6644
H	6.8622	2.4572	0.1624	H	-2.8255	2.9378	4.8622
H	7.1017	3.1632	-1.4500	H	-3.9244	1.6888	5.5150
H	6.7756	4.2312	-0.0537	H	-2.5530	1.2079	4.5027
C	-0.9523	-0.0936	0.4073	C	0.9674	-0.2118	0.5863
C	-0.4024	0.2814	1.6171	C	0.4733	-0.2993	1.8721
C	0.9655	0.2686	1.8460	C	-0.8805	-0.4498	2.1409
C	1.7953	-0.0143	0.7752	C	-1.7611	-0.3838	1.0751
C	1.2832	-0.2729	-0.5264	C	-1.3092	-0.1574	-0.2548
C	-0.1048	-0.3995	-0.6516	C	0.0722	-0.1777	-0.4761
H	-2.0190	-0.1103	0.2711	H	2.0267	-0.1449	0.4107
H	1.3384	0.5249	2.8224	H	-1.2023	-0.5695	3.1626
C	3.6042	-0.0122	-0.7096	C	-3.6408	0.0507	-0.2542
C	4.8313	0.1398	-1.3353	C	-4.9007	0.3531	-0.7516
C	4.8540	0.1054	-2.7229	C	-4.9829	0.8100	-2.0588
C	3.7102	-0.0799	-3.4882	C	-3.8681	0.9645	-2.8731
C	2.4818	-0.2473	-2.8582	C	-2.6128	0.6327	-2.3844
C	2.4198	-0.2290	-1.4671	C	-2.4904	0.1613	-1.0787
H	5.7509	0.2972	-0.7993	H	-5.7994	0.2717	-0.1643

H	3.7933	-0.1001	-4.5648	H	-3.9977	1.3366	-3.8783
H	1.5993	-0.4135	-3.4511	H	-1.7526	0.7266	-3.0228
C	3.2619	0.0403	0.7135	C	-3.2363	-0.3661	1.0890
C	4.1169	0.0928	1.7778	C	-4.0496	-0.6904	2.1385
C	5.5278	0.1547	1.6224	C	-5.4652	-0.5825	2.0717
C	3.6762	0.0287	3.1289	C	-3.5507	-1.1945	3.3705
N	6.6754	0.2176	1.5016	N	-6.6154	-0.4860	2.0208
N	3.3247	-0.0368	4.2273	N	-3.1479	-1.6073	4.3718
N	-1.2905	0.7322	2.6979	N	1.4152	-0.2224	2.9997
O	-0.7663	1.2519	3.6757	O	0.9375	-0.1568	4.1247
O	-2.4938	0.5812	2.5455	O	2.6104	-0.2088	2.7404
N	-0.7645	-0.8571	-1.8839	N	0.6753	-0.1969	-1.8187
O	-1.9721	-0.6869	-1.9778	O	-0.0494	-0.4340	-2.7750
O	-0.0825	-1.4197	-2.7294	O	1.8821	-0.0232	-1.9002
N	6.1486	0.2689	-3.4045	N	-6.3065	1.1505	-2.6005
O	6.1686	0.1548	-4.6222	O	-7.2834	0.9420	-1.8867
O	7.1320	0.4981	-2.7084	O	-6.3599	1.6114	-3.7316

Z-**2**·**10** complex G				*Z*-**2**·**10** complex H				*Z*-**2**·**10** I			
E = -4085.49114633				E = -4085.49101506				E = -4085.49009954			
C	5.1402	1.9799	-1.0397	C	-4.3606	2.7148	-0.4774	C	-5.1333	2.0031	-1.0721
O	8.1835	0.1572	-1.1313	O	-7.6939	1.9027	-1.4163	O	-8.1988	0.2205	-1.2128
C	4.4634	3.1772	-1.2847	C	-3.3434	3.6715	-0.4352	C	-4.4386	3.1935	-1.2998
C	7.2501	2.9205	-1.7452	C	-5.9318	4.2937	-1.4237	C	-7.2206	2.9730	-1.8056
C	6.5983	4.1198	-2.0050	C	-4.9392	5.2648	-1.3870	C	-6.5501	4.1651	-2.0503
C	5.2214	4.2421	-1.7722	C	-3.6650	4.9499	-0.9009	C	-5.1758	4.2696	-1.7949
C	6.5110	1.8467	-1.2514	C	-5.6355	3.0135	-0.9613	C	-6.5025	1.8881	-1.3051
C	7.0330	0.5072	-0.9155	C	-6.5651	1.8659	-0.9502	C	-7.0467	0.5547	-0.9806
C	5.9380	-0.3504	-0.3477	C	-5.8923	0.6599	-0.3719	C	-5.9712	-0.3186	-0.4009
C	6.0273	-1.6126	0.1585	C	-6.4072	-0.5859	-0.1764	C	-6.0840	-1.5810	0.0997
C	7.3269	-2.3730	0.5130	C	-7.8936	-1.0040	-0.1832	C	-7.3969	-2.3252	0.4382
C	3.1279	-4.5175	1.2354	C	-4.5449	-4.2812	0.7350	C	-3.2337	-4.5263	1.1956
C	4.4783	-4.6382	1.5487	C	-5.9203	-4.0781	0.8444	C	-4.5881	-4.6288	1.4990
C	5.3549	-3.6296	1.1737	C	-6.4542	-2.8340	0.5334	C	-5.4483	-3.6083	1.1180
C	6.8266	-3.5405	1.4195	C	-7.8800	-2.3771	0.5677	C	-6.9212	-3.4994	1.3498
C	3.5366	-2.4011	0.1458	C	-4.2479	-2.0182	-0.0381	C	-3.6060	-2.4038	0.1050
C	2.6472	-3.4041	0.5314	C	-3.7010	-3.2586	0.2812	C	-2.7333	-3.4194	0.4955
C	7.9382	-2.9595	-0.7761	C	-8.8334	-0.0390	0.5517	C	-8.4292	-1.4884	1.2071
C	4.8964	-2.4970	0.4802	C	-5.6181	-1.7857	0.1186	C	-4.9688	-2.4812	0.4299
C	8.3602	-1.5485	1.2939	C	-8.3505	-1.2194	-1.6405	C	-8.0001	-2.9029	-0.8586
H	2.4317	-5.2970	1.5240	H	-4.1188	-5.2472	0.9805	H	-2.5508	-5.3162	1.4873
H	7.0142	-3.3056	2.4727	H	-8.5660	-3.0934	0.1084	H	-7.4565	-4.4259	1.1263
H	7.3514	-4.4742	1.2011	H	-8.2060	-2.2389	1.6040	H	-7.1160	-3.2615	2.4010
H	8.8228	-3.5525	-0.5255	H	-9.8121	-0.5149	0.6661	H	-9.2153	-2.1525	1.5796
H	7.2204	-3.6122	-1.2807	H	-8.4497	0.1852	1.5507	H	-7.9629	-1.0028	2.0685
H	8.2352	-2.1621	-1.4544	H	-8.9657	0.8905	0.0065	H	-8.8905	-0.7320	0.5801
H	8.8384	-0.7981	0.6726	H	-8.3014	-0.2836	-2.1948	H	-8.2789	-2.1011	-1.5394
H	7.8896	-1.0571	2.1497	H	-7.7228	-1.9639	-2.1376	H	-7.2847	-3.5640	-1.3554
H	9.1335	-2.2222	1.6759	H	-9.3836	-1.5787	-1.6526	H	-8.8951	-3.4850	-0.6192
H	8.3131	2.7958	-1.9134	H	-6.9257	4.4989	-1.8028	H	-8.2824	2.8621	-1.9902
H	7.1523	4.9690	-2.3860	H	-5.1447	6.2692	-1.7364	H	-7.0876	5.0226	-2.4363
H	4.7240	5.1852	-1.9703	H	-2.8962	5.7144	-0.8764	H	-4.6641	5.2077	-1.9797
S	4.3757	0.5226	-0.4409	S	-4.1885	1.0481	0.0438	S	-4.3957	0.5337	-0.4691
H	4.8405	-5.5115	2.0797	H	-6.5652	-4.8878	1.1674	H	-4.9661	-5.4979	2.0260
H	3.1580	-1.5783	-0.4393	H	-3.6011	-1.2603	-0.4504	H	-3.2111	-1.5856	-0.4765
C	3.0026	3.2747	-1.0020	C	-1.9721	3.3679	0.0711	C	-2.9823	3.2759	-0.9904
C	2.5671	3.6313	0.2870	C	-0.9212	3.1644	-0.8423	C	-2.5679	3.5974	0.3147
C	2.0668	2.9623	-2.0011	C	-1.7115	3.3457	1.4536	C	-2.0303	2.9887	-1.9818
C	1.2000	3.6933	0.5466	C	0.3763	3.0018	-0.3634	C	-1.2051	3.6533	0.5977
C	0.7072	3.0222	-1.6983	C	-0.3970	3.1925	1.8931	C	-0.6757	3.0429	-1.6565
C	0.2430	3.3896	-0.4302	C	0.6732	3.0376	1.0043	C	-0.2326	3.3781	-0.3720
H	0.8849	3.9955	1.5367	H	1.1685	2.8292	-1.0808	H	-0.9061	3.9298	1.6002
H	0.0021	2.7624	-2.4775	H	-0.2077	3.2360	2.9580	H	0.0422	2.8043	-2.4307
C	1.1899	-3.3067	0.2140	C	-2.2294	-3.4464	0.1243	C	-1.2758	-3.3365	0.1777
C	0.2766	-2.9801	1.2300	C	-1.6725	-3.5727	-1.1612	C	-0.8161	-3.5688	-1.1319
C	0.7187	-3.5682	-1.0859	C	-1.3895	-3.4214	1.2519	C	-0.3538	-3.0385	1.1947
C	-1.0836	-2.9055	0.9320	C	-0.2884	-3.6455	-1.2968	C	0.5494	-3.4941	-1.3981
C	-0.6500	-3.5023	-1.3417	C	-0.0080	-3.4648	1.0712	C	1.0040	-2.9569	0.8872
C	-1.5795	-3.1654	-0.3494	C	0.5736	-3.5582	-0.1975	C	1.4862	-3.1782	-0.4062
H	-1.7603	-2.6107	1.7234	H	0.1201	-3.7649	-2.2916	H	0.8841	-3.7050	-2.4057
H	-0.9921	-3.7435	-2.3401	H	0.6226	-3.3699	1.9462	H	1.6881	-2.6834	1.6800
C	-3.0321	-3.0629	-0.6468	C	2.0449	-3.4866	-0.3799	C	2.9326	-3.0542	-0.7228
C	-3.4811	-2.9178	-1.9745	C	2.9143	-3.7375	0.6899	C	3.8928	-3.0792	0.2949
C	-3.9688	-3.0768	0.3838	C	2.5723	-3.0763	-1.6105	C	3.3612	-2.8794	-2.0433
C	-4.8445	-2.8337	-2.2451	C	4.2872	-3.5389	0.5321	C	5.2446	-2.9374	-0.0072
H	-2.7688	-2.8539	-2.7806	H	2.5225	-4.0768	1.6355	H	3.6259	-3.2188	1.3323
C	-5.3338	-2.9498	0.1046	C	3.9499	-2.9049	-1.7598	C	4.7199	-2.7782	-2.3449
H	-3.6819	-3.1935	1.4186	H	1.9089	-2.8261	-2.4225	H	2.6676	-2.8105	-2.8679

C	-5.7842	-2.8492	-1.2052	C	4.8119	-3.1227	-0.6912	C	5.6811	-2.8041	-1.3291
H	-6.8281	-2.7536	-1.4635	H	5.8762	-2.9682	-0.8020	H	6.7294	-2.7037	-1.5583
C	-1.2103	3.4241	-0.1212	C	2.0684	2.9061	1.4913	C	1.2155	3.4115	-0.0401
C	-1.6552	3.4103	1.2055	C	3.1411	3.2009	0.6385	C	2.1869	3.4719	-1.0464
C	-2.1666	3.4438	-1.1436	C	2.3274	2.4625	2.7945	C	1.6398	3.3587	1.2925
C	-3.0153	3.4208	1.4988	C	4.4559	3.0466	1.0860	C	3.5434	3.4870	-0.7238
H	-0.9683	3.3599	2.0363	H	2.9469	3.5744	-0.3541	H	1.9251	3.5284	-2.0930
C	-3.5282	3.4564	-0.8430	C	3.6477	2.3313	3.2380	C	2.9948	3.3716	1.6073
H	-1.8893	3.4702	-2.1874	H	1.5091	2.1938	3.4436	H	0.9402	3.2764	2.1101
C	-3.9719	3.4597	0.4806	C	4.7145	2.6178	2.3839	C	3.9665	3.4517	0.6061
H	-5.0243	3.4575	0.7125	H	5.7333	2.5007	2.7263	H	5.0151	3.4520	0.8546
C	3.5620	3.9269	1.3812	C	-1.1884	3.1068	-2.3262	C	-3.5796	3.8630	1.4012
H	4.2357	4.7392	1.0983	H	-1.5224	4.0731	-2.7124	H	-4.1957	2.9784	1.5853
H	3.0555	4.2003	2.3068	H	-0.2847	2.8236	-2.8698	H	-3.0875	4.1253	2.3378
H	4.1855	3.0527	1.5885	H	-1.9711	2.3811	-2.5571	H	-4.2586	4.6729	1.1241
C	2.5220	2.5331	-3.3719	C	-2.8242	3.4879	2.4606	C	-2.4629	2.5897	-3.3691
H	3.0919	1.6024	-3.3144	H	-3.5351	4.2630	2.1689	H	-3.0997	3.3527	-3.8230
H	1.6694	2.3657	-4.0315	H	-3.3797	2.5494	2.5471	H	-1.5993	2.4316	-4.0166
H	3.1701	3.2845	-3.8292	H	-2.4267	3.7319	3.4466	H	-3.0378	1.6607	-3.3402
C	0.7481	-2.6972	2.6345	C	-2.5421	-3.6038	-2.3944	C	-1.7807	-3.8862	-2.2451
H	1.0078	-3.6229	3.1566	H	-3.4465	-4.1931	-2.2348	H	-2.5070	-4.6427	-1.9402
H	1.6436	-2.0739	2.6348	H	-2.8565	-2.5944	-2.6757	H	-2.3444	-2.9943	-2.5323
H	-0.0321	-2.1983	3.2105	H	-1.9946	-4.0253	-3.2388	H	-1.2495	-4.2474	-3.1271
C	1.6756	-3.9133	-2.1972	C	-1.9578	-3.3085	2.6455	C	-0.8139	-2.7874	2.6093
H	2.2563	-3.0350	-2.4923	H	-2.7499	-2.5607	2.6970	H	-1.7073	-2.1614	2.6301
H	2.3882	-4.6802	-1.8854	H	-2.3892	-4.2591	2.9728	H	-1.0724	-3.7237	3.1126
H	1.1378	-4.2735	-3.0756	H	-1.1801	-3.0353	3.3612	H	-0.0279	-2.3036	3.1904
O	-3.3360	3.3341	2.8228	O	5.5497	3.2549	0.3041	O	4.3995	3.4931	-1.7833
O	-6.1478	-2.8944	1.1958	O	4.5293	-2.4613	-2.9126	O	5.0166	-2.6330	-3.6603
O	-5.3745	-2.6999	-3.4853	O	5.1947	-3.6891	1.5341	O	6.0830	-2.9038	1.0685
O	-4.3670	3.4185	-1.9154	O	3.9906	1.9039	4.4766	O	3.2963	3.2452	2.9329
C	-4.4883	-2.5864	-4.5961	C	4.7806	-4.3258	2.7409	C	7.4885	-2.8768	0.8343
H	-3.8813	-3.4912	-4.7095	H	4.3632	-5.3165	2.5355	H	7.7882	-1.9683	0.3038
H	-5.1284	-2.4695	-5.4680	H	5.6823	-4.4254	3.3414	H	7.9501	-2.8907	1.8197
H	-3.8379	-1.7139	-4.5003	H	4.0539	-3.7174	3.2855	H	7.8105	-3.7573	0.2693
C	-5.7555	3.6404	-1.7036	C	2.9522	1.6157	5.4105	C	4.6244	3.5487	3.3558
H	-6.2207	3.5634	-2.6833	H	3.4586	1.3128	6.3240	H	4.6069	3.4967	4.4424
H	-6.1960	2.8874	-1.0434	H	2.3419	2.5036	5.6040	H	5.3453	2.8172	2.9821
H	-5.9337	4.6349	-1.2816	H	2.3193	0.7955	5.0634	H	4.9124	4.5558	3.0390
C	-7.5557	-2.8264	0.9792	C	3.7943	-2.5918	-4.1257	C	6.3810	-2.5022	-4.0446
H	-8.0042	-2.8010	1.9703	H	4.4684	-2.2621	-4.9142	H	6.3710	-2.4109	-5.1286
H	-7.8300	-1.9225	0.4274	H	3.5056	-3.6338	-4.2953	H	6.8301	-1.6040	-3.6125
H	-7.9130	-3.7079	0.4380	H	2.9022	-1.9609	-4.1331	H	6.9591	-3.3881	-3.7600
C	-4.6749	3.6316	3.2149	C	5.3802	3.9223	-0.9415	C	5.7820	3.7166	-1.5412
H	-5.3794	2.8785	2.8531	H	4.9763	4.9290	-0.7935	H	6.2181	2.9409	-0.9043
H	-4.6743	3.6137	4.3027	H	6.3723	3.9764	-1.3828	H	6.2628	3.6826	-2.5159
H	-4.9718	4.6240	2.8622	H	4.7231	3.3615	-1.6125	H	5.9467	4.6946	-1.0770
C	-5.3470	0.1854	0.8504	C	-0.7725	-0.2316	0.2075	C	5.3432	0.1842	0.8852
C	-5.0931	0.2212	-0.5091	C	-0.1920	-0.1809	1.4605	C	5.0983	0.2559	-0.4752
C	-3.8069	0.2131	-1.0326	C	1.1814	-0.2476	1.6485	C	3.8164	0.2486	-1.0124
C	-2.7470	0.2027	-0.1397	C	1.9893	-0.2115	0.5248	C	2.7506	0.2058	-0.1280
C	-2.9573	0.2100	1.2632	C	1.4421	-0.1346	-0.7831	C	2.9506	0.1739	1.2769
C	-4.2714	0.1520	1.7261	C	0.0545	-0.2638	-0.9077	C	4.2616	0.1158	1.7498
H	-6.3579	0.1590	1.2244	H	-1.8423	-0.2317	0.0936	H	6.3512	0.1537	1.2665
H	-3.6820	0.2390	-2.1014	H	1.5745	-0.2716	2.6493	H	3.6990	0.2899	-2.0822
C	-0.6456	0.2933	0.9166	C	3.7500	0.2239	-0.9609	C	0.6415	0.2450	0.9160
C	0.6924	0.4609	1.2411	C	4.9520	0.5643	-1.5624	C	-0.6997	0.4000	1.2354
C	0.9844	0.7481	2.5712	C	4.9181	0.8848	-2.9145	C	-1.0021	0.6543	2.5693
C	0.0161	0.8567	3.5587	C	3.7489	0.8834	-3.6635	C	-0.0416	0.7396	3.5673
C	-1.3178	0.6204	3.2403	C	2.5453	0.5352	-3.0567	C	1.2949	0.5160	3.2528
C	-1.6490	0.3419	1.9214	C	2.5410	0.1872	-1.7097	C	1.6371	0.2740	1.9289
H	1.4893	0.4144	0.5145	H	5.8866	0.6173	-1.0272	H	-1.4911	0.3697	0.5022

H	0.3154	1.1124	4.5654	H	3.7945	1.1463	-4.7105	H	-0.3492	0.9696	4.5776
H	-2.0695	0.6759	4.0097	H	1.6386	0.5170	-3.6395	H	2.0407	0.5557	4.0289
C	-1.2971	0.1569	-0.3939	C	3.4531	-0.1204	0.4344	C	1.3026	0.1541	-0.3935
C	-0.7028	-0.0403	-1.6037	C	4.3445	-0.3920	1.4295	C	0.7171	-0.0038	-1.6127
C	0.6885	-0.2588	-1.7969	C	5.7548	-0.3370	1.2436	C	-0.6718	-0.2359	-1.8110
C	-1.4261	-0.0924	-2.8300	C	3.9339	-0.8535	2.7116	C	1.4438	0.0187	-2.8380
N	1.7856	-0.4819	-2.0775	N	6.9005	-0.2993	1.1072	N	-1.7698	-0.4649	-2.0823
N	-1.9634	-0.1265	-3.8519	N	3.6002	-1.2572	3.7409	N	1.9849	0.0602	-3.8578
N	-6.2334	0.2619	-1.4401	N	-1.0555	-0.0526	2.6419	N	6.2438	0.3137	-1.3911
O	-5.9887	0.3282	-2.6326	O	-0.5071	0.0691	3.7314	O	6.0108	0.4365	-2.5842
O	-7.3599	0.2456	-0.9528	O	-2.2646	-0.0571	2.4648	O	7.3684	0.2371	-0.9051
N	-4.6030	-0.0130	3.1467	N	-0.6116	-0.4633	-2.2004	N	4.5838	-0.0889	3.1674
O	-5.6453	0.4888	3.5461	O	-1.8102	-0.2335	-2.2674	O	5.6193	0.4088	3.5894
O	-3.8344	-0.6737	3.8316	O	0.0627	-0.8980	-3.1279	O	3.8159	-0.7759	3.8264
N	2.3871	0.9339	2.9688	N	6.1792	1.2667	-3.5790	N	-2.4080	0.8343	2.9588
O	3.2222	0.2382	2.4099	O	6.1842	1.2960	-4.8022	O	-2.6452	1.6444	3.8462
O	2.6191	1.7653	3.8377	O	7.1337	1.5365	-2.8637	O	-3.2404	0.1567	2.3745
